# The [M_6_(S_2_C_2_Ph_2_)_6_] (M = Ni, Pd, Pt) Series: Multielectron Reservoirs That Sustain Ligand-Based Oxidations and Metal-Based Reductions

**DOI:** 10.1021/acs.inorgchem.5c03536

**Published:** 2025-11-24

**Authors:** Titir Das Gupta, Brian Wuille Bille, Olaf Ruediger, Xiaodong Zhang, Joel T. Mague, Serena DeBeer, James P. Donahue

**Affiliations:** § Department of Chemistry, 5783 Tulane University, 6400 Freret Street, New Orleans, Louisiana 70118, United States; † Max-Planck-Institut für Chemische Energiekonversion, Stiftstrasse 34-36, D-45470 Mülheim an der Ruhr, Germany

## Abstract

A complete [M_6_(S_2_C_2_R_2_)_6_] series (M = Ni (**1**), Pd (**2**), Pt (**3**); R = Ph), the rarest variety among homoleptic dithiolene transition-metal compounds, has been prepared by reaction between [M­(S_2_C_2_Ph_2_)_2_] and a M^0^ source. The platinum member of this set is the first of its type. Diffraction-quality crystals, grown with high reproducibility by evaporation from PhNO_2_ solutions, reveal fully reduced [Ph_2_C_2_S_2_]^2–^ dianions and an octahedral M_6_ core that is reduced to *C*_2_ symmetry by the fusion of a mononuclear *D*_2*h*_ [M­(S_2_C_2_Ph_2_)_2_] fragment upon a *C*_4_-symmetric base. The [Ni_6_(S_2_C_2_Ph_2_)_2_]^1–^ monoanion, prepared by Cp*_2_Co reduction, shows only modest structural differences from its neutral counterpart. In CH_2_Cl_2_, **1** and **2** can undergo two reductions and an oxidation, while **3** sustains two reductions and two oxidations. In benzonitrile, **1** sustains three reversible oxidations at potentials that are shifted appreciably to less positive values. The cathodic processes are shown by density functional theory (DFT) calculations to involve an MO largely of metal–sulfur composition that has contributions throughout the *C*_4_-symmetric pentametallic base of the assembly, while the oxidations are largely ligand-based and confined to the monometallic [M­(S_2_C_2_Ph_2_)_2_] cap. The absorption spectra are marked by multiple overlapping bands that produce a continuous, tapering absorption profile of unresolved shoulders and swells.

## Graphical abstract

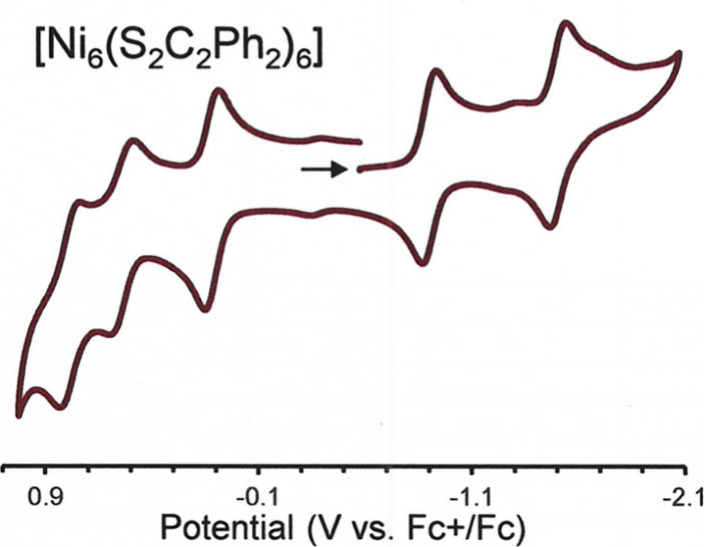

## Introduction

A capacity to deliver or accept multiple reducing equivalents at the same—or similar—mild potential, often coupled with the transfer of H^+^, is a critical feature for the optimal functioning of redox enzymes such as assimilatory sulfite reductase and the Ni-FeS carbon monoxide dehydrogenase. In the former, multiple flavoprotein subunits mediate electron transfer to the siroheme-(μ-SCys)-Fe_4_S_4_ active site,^1^ while in the latter, a pathway involving a series of Fe_4_S_4_ clusters channels the electrons associated with CO oxidation to an exogenous acceptor.^2^ These and other multielectron redox enzymes suggest that the incorporation of multielectron reservoirs proximal to a catalytic site is a general design principle for the creation of new systems for multielectron redox catalysis. Molecular systems that support multiple, reversible electron transfers elicit interest also for applications in charge storage^3^ and redox flow batteries,^4^ switching devices in molecular electronics,^5^ and stable mixed-valency that supports low-energy optical absorption^6^ and electrical conduction in the solid state.^7^

An ability to sustain successive, highly reversible electron transfer processes involving highly delocalized ligand-based or mixed metal–ligand MOs is a defining characteristic of homoleptic transition-metal dithiolene complexes. In the now extensive corpus of synthetic and structural chemistry on metallodithiolene complexes, the preponderating coordination geometry motifs are the mononuclear trigonal prism,^8^ square plane^9^ and the dinuclear square pyramid^10^ (Figures 1a–c, respectively). Unique to the Group 10 elements, however, is a hexametallic, cagelike structure in which the metal-to-ligand ratio is 1:1. Although it was misformulated as a tetrameric species, the first documented observation of a [Ni_6_(S_2_C_2_R_2_)_6_] cage was offered by Schrauzer, who noted the appearance of [Ni_6_(S_2_C_2_Ph_2_)_6_] in the extrusion of a phenyldithiolene ligand from [Ni­(S_2_C_2_Ph_2_)_2_] upon reaction with 1 equiv of an alkyne and also as an outcome from treating [Ni­(S_2_C_2_Ph_2_)_2_] with Ni powder or [Ni­(CO)_4_].^11^ Recently, Morris and co-workers reported [Ni_6_(S_2_C_2_Ph_2_)_6_] as a byproduct from dithiolene ligand transfer from [Ni­(S_2_C_2_Ph_2_)_2_] to [Fe­(CO)_5_] and identified it structurally as a *C*_2_-symmetric cage compound. While they observed the occurrence of two reversible reductions in the cyclic voltammogram of [Ni_6_(S_2_C_2_Ph_2_)_6_], anodic scanning revealed multiple features of a more complex description.^12^ Without noting its connection to Schrauzer’s work, Stiefel and co-workers had somewhat earlier reported a deliberate synthesis of [Pd_6_(S_2_C_2_(CO_2_Me)_2_)_6_] by a transmetalation reaction between [PdCl_2_(MeCN)_2_] and [(tmeda)­Zn­(S_2_C_2_(CO_2_Me)_2_)] (where tmeda = tetramethylethylene diamine), which revealed a striking series of four highly reversible reductions in its cyclic voltammogram.^13^ The fully reduced ene-1,2-dithiolate redox state that was indicated for the dithiolene ligands by the intraligand S–C and C–C_chelate_ bond lengths implicated these reductions as metal-based processes, in intriguing contrast to the ligand-based redox chemistry that is typical of metallodithiolene complexes. Since Stiefel’s report, [Pd_6_(S_2_C_6_H_2_-4,5-(OMe)_2_)_6_][Bibr ref14] and [Pd_6_(S_2_C_2_(CF_3_)_2_)_6_][Bibr ref15] have appeared as additional examples of hexapalladium dithiolene cages. The prospect of both metal-based reductive chemistry and of ligand-based oxidative processes, in the context of the larger significance of multielectron reservoirs and of the mixed-valence systems noted above, has prompted an intentional study of the [M_6_(S_2_C_2_R_2_)_6_] complexes both as the metal atom identity is varied and as other redox states are accessed. In this report, we detail the syntheses and structures of [M_6_(S_2_C_2_Ph_2_)_6_]^*n*^ (M = Ni, Pd, Pt and *n* = 0; M = Ni, *n* = −1) and reveal the variation in electrochemical and spectroscopic behavior as a function of metal.

**Figure 1:**
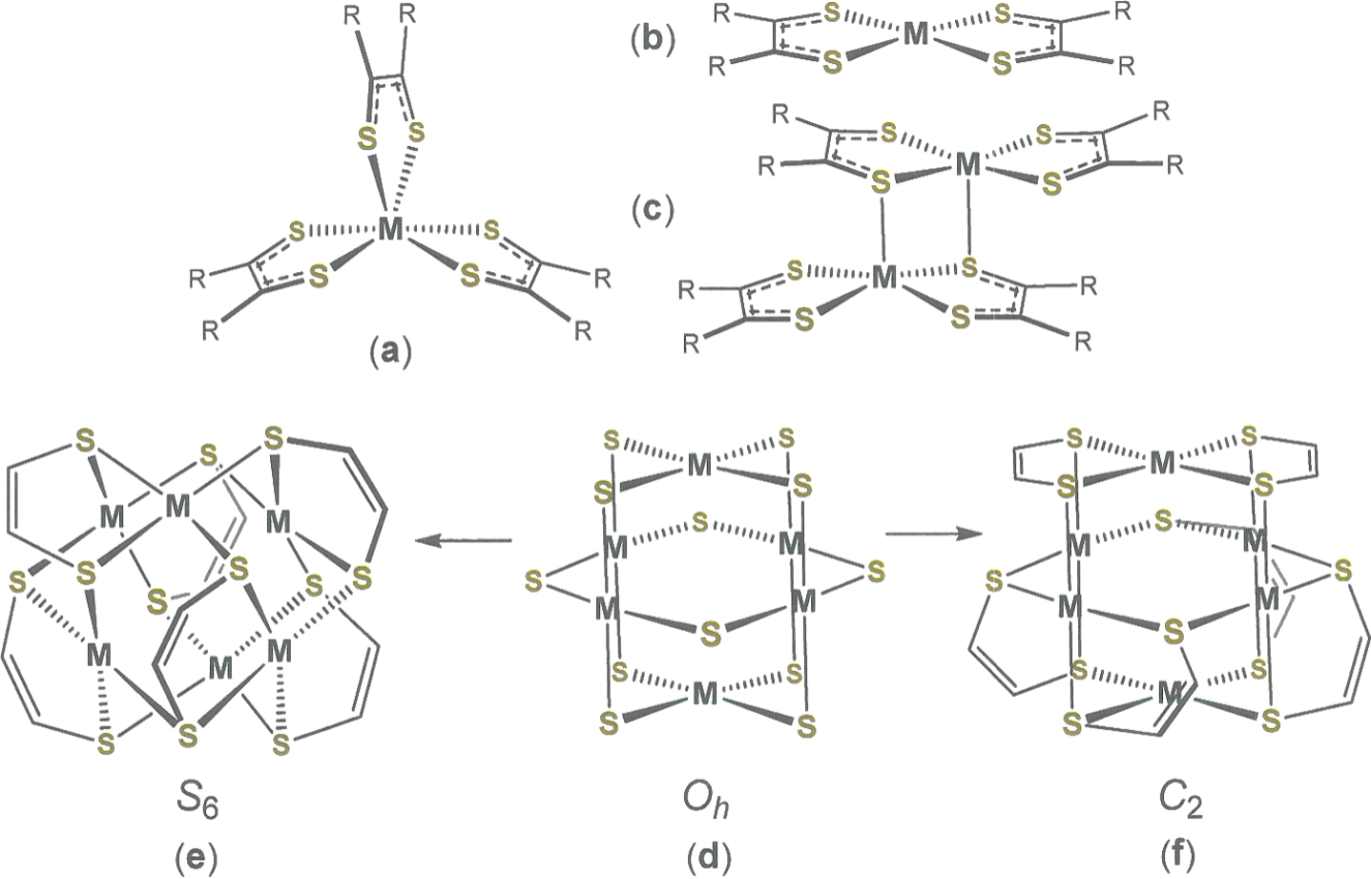
(a–c) Common structural motifs among homoleptic transition metal dithiolene complexes; (d) core structure of the octahedral hexametallic cage complexes and (e) and (f) the two possible point groups to which the symmetry is reduced upon imposition of the olefinic backbone,.

## Experimental Section

### Physical Methods and General Considerations

The ^1^H and ^13^C NMR spectra were recorded at 25 °C with a Bruker Ascend spectrometer operating at 400 and 100 MHz, respectively, and were referenced to the solvent residual. The UV-vis spectra were acquired at ambient temperature with an Agilent Cary 60 spectrophotometer, while the mass spectra were obtained either with a Bruker ultraflextreme MALDI-TOF-MS instrument or with a Bruker micrOTOF II mass spectrometer operating with an Agilent Technologies 1200 Series LC liquid chromatography system. Electrochemical measurements were performed using a CHI 620C electrochemical analyzer workstation with a Ag/AgCl reference electrode, Pt disk working electrode, Pt wire as an auxiliary electrode, and [^*n*^Bu_4_N]­[PF_6_] as the supporting electrolyte with CH_2_Cl_2_ or benzonitrile as the solvent. Under these conditions, the Cp_2_Fe^+^/Cp_2_Fe couple occurred at approximately +0.60 V, relative to Ag/AgCl. The elemental analysis was performed by the Kolbe Microanalytical Laboratory of Oberhausen, Germany. Procedural details regarding crystal growth, X-ray diffraction (XRD) data collection, data processing, and structure solution and refinement are deferred to the Supporting Information (SI), as is a description of the computational methods that have been employed.

### Syntheses

No uncommon hazards were noted in this work. Literature procedures were followed for the preparation of [PdCl_2_(MeCN)_2_],^16^ dibenzylideneacetone (dba),^17^ Pt_2_(dba)_3_,^18^ and [^*n*^Bu_2_Sn­(S_2_C_2_Ph_2_)].^19^ Solvents were dried with a system of drying columns from the Glass Contour Company (CH_2_Cl_2_, THF, Et_2_O, toluene, and *n*-pentane) or were purchased in anhydrous form from commercial suppliers (MeCN). All solvents used in the column chromatograph purifications, the nitrobenzene used as crystallization solvent, and all other reagents were used as supplied from commercial vendors without further measures to purify or dry them.

### [Ni_6_(S_2_C_2_Ph_2_)_6_] (**1**)

Under an atmosphere of N_2_, [Ni­(S_2_C_2_Ph_2_)_2_] (0.6534 g, 1.202 mmol) and powdered Ni (0.7043 g, 12.00 mmol) were combined in a 100 mL Schlenk flask with acetonitrile (30 mL) and refluxed at 90 °C for 72 h, during which time the reaction mixture transitioned from a dark green to a dark brown color and a dark solid precipitated. After this mixture was cooled to ambient temperature, the solid precipitate was collected by filtration and washed with MeCN (3 × 10 mL), followed by Et_2_O (3 × 10 mL). This crude solid was redissolved in a minimal volume of dry CH_2_Cl_2_, filtered, and evaporated onto ∼2 g of silica. This coated silica was then dry loaded onto a column packed as a slurry in petroleum ether. Elution with 1:5 CH_2_Cl_2_:petroleum ether first moved [Ni­(C_2_S_2_Ph_2_)_2_] from the column as the leading green band. Continued elution with 3:7 CH_2_Cl_2_:hexanes subsequently brought down **1** as a brown fraction. Following the collection of **1** and the removal of all volatiles, **1** was redissolved in a minimal volume of nitrobenzene and filtered through packed Celite. The Celite was washed with a small portion of fresh nitrobenzene. Brown, block-shaped crystals of **1**·7PhNO_2_ formed by slow evaporation of this nitrobenzene solution at ambient pressure and temperature from a scintillation vial. Yield: 0.27 g, 0.15 mmol, 37%. *R*_f_ = 0.38 (2:3 CH_2_Cl_2_:petroleum ether). ^1^H NMR (δ, ppm in CD_2_Cl_2_): 7.48 (d, 4 H, phenyl C–*H*), 7.32–7.27 (m, 9 H, phenyl C–*H*), 7.25–7.18 (m, 13 H, phenyl C–*H*), 7.16–7.08 (m, 26 H, phenyl C–*H*), 7.03–6.95 (m, 8 H, phenyl C–*H*). ^13^C NMR (CD_2_Cl_2_): δ 145.98, 145.11, 142.30, 142.06, 141.65, 140.34, 139.40, 138.98, 138.92, 138.58, 138.42, 137.79, 130.35, 130.13, 130.07, 129.99, 129.69, 129.25, 128.68, 128.56, 128.50, 128.40, 128.29, 128.27, 128.24, 128.09, 128.03. UV–vis [CH_2_Cl_2_, λ_max_, nm (*ε*_M_, M^–1^ cm^–1^)]: 270 (12100), 350 (7300), 445 (2900). MS (ESI^+^) Calcd for C_84_H_60_S_12_Ni_6_: *m*/*z* 1805.7377; Observed: *m*/*z* 1805.6855; Error (δ) 28.92 ppm. Cyclic voltammetry (CH_2_Cl_2_, [^*n*^Bu_4_N]­[PF_6_] supporting electrolyte, [Cp_2_Fe]^+^/Cp_2_Fe reference): **1** – e^–^ → [**1**]^+^, +0.385 V; **1** + e^–^ → [**1**]^−^, −1.127 V; [**1**]^−^ + e^–^ → [**1**]^2–^, −1.679 V. Cyclic voltammetry (PhCN, [^*n*^Bu_4_N]­[PF_6_] supporting electrolyte, [Cp_2_Fe]^+^/Cp_2_Fe reference): **1** – e^–^ → [**1**]^+^, +0.118 V; [**1**]^+^ – e^–^ → [**1**]^2+^, +0.543 V; [**1**]^2+^ – e^–^ → [**1**]^3+^, +0.783 V; **1** + e^–^ → [**1**]^−^, −0.905 V; [**1**]^−^ + e^–^ → [**1**]^2–^, −1.506 V. Anal. Calcd for C_84_H_60_S_12_Ni_6_: C, 55.85; H, 3.50; S, 21.21. Found: C, 55.61; H, 3.41; S, 21.21. The identity of this compound was corroborated by X-ray crystallographic characterization (JPD Data Set 1236).

### [Cp*_2_Co]­[Ni_6_(S_2_C_2_Ph_2_)_6_], [Cp*_2_Co] (**1**)

This reaction and all subsequent manipulations were conducted under an atmosphere of dry N_2_. An oven-dried 50 mL Schlenk flask with stir bar was charged with [Ni_6_(S_2_C_2_Ph_2_)_6_] (0.0516 g, 0.0286 mmol), decamethylcobaltocene (0.020 g, 0.0607 mmol), and THF (20 mL) and the resulting reaction mixture was stirred for 2 h at ambient temperature. All volatiles were removed under reduced pressure, and the solid residue was washed with *n*-pentane (20 mL). The brown solid that remained was dried under vacuum, redissolved in MeCN (5 mL) and filtered through packed Celite. Diffusion of Et_2_O vapor into the MeCN filtrate produced plate-shaped black crystals of [Cp*_2_Co]­[**1**]·MeCN·3/4Et_2_O. The formulation of this compound was established by X-ray crystallography (JPD Data Set JPD1483).

### [Pd_6_(S_2_C_2_Ph_2_)_6_] (**2**), Method A

In an oven-dried 100 mL Schlenk flask equipped with a stir bar, [Pd­(S_2_C_2_Ph_2_)_2_] (0.072 g, 0.122 mmol), Pd_2_(dba)_3_ (0.2055 g, 0.224 mmol), and toluene (30 mL) were combined and heated to 90 °C for 72 h under a N_2_ atmosphere. The reaction mixture was then cooled, and the solvent was removed under reduced pressure. The crude solid was redissolved in a minimal volume of dry CH_2_Cl_2_, filtered, and evaporated onto ∼2 g of silica. This coated silica was then dry-loaded onto a column packed as a slurry in hexanes. Elution of the column with 1:5 CH_2_Cl_2_:petroleum ether moved [Pd­(C_2_S_2_Ph_2_)_2_] as a leading blue band, and continued elution with 3:7 CH_2_Cl_2_:hexanes enabled collection of **2** as a light green band. All volatiles were removed under reduced pressure, and the solid residue of **2** was redissolved in a minimal volume of nitrobenzene. This concentrated solution was filtered through packed Celite, and the filtrate was evaporated at ambient temperature and pressure to afford black crystals of **2**·PhNO_2_. Yield: 0.015 g, 0.00677 mmol, 17%. An X-ray crystal structure confirmed the hexameric structure of **2** and its composition as a monosolvate of PhNO_2_ (Data set JPD1216).

### [Pd_6_(S_2_C_2_Ph_2_)_6_] (**2**), Method B

An oven-dried 100 mL Schlenk flask with a stir bar was charged with solid [^*n*^Bu_2_Sn­(S_2_C_2_Ph_2_)] (0.300 g, 0.631 mmol) and [PdCl_2_(MeCN)_2_], (0.1637 g, 0.631 mmol) under an outward flow of N_2_. Anhydrous MeCN (30 mL) was added via a syringe, and the resulting solution assumed a very dark color. The reaction mixture was left to reflux under N_2_ for 24 h, after which time it was cooled. The solid precipitate that formed was collected by filtration, dissolved in a minimal volume of CH_2_Cl_2_, evaporated onto ∼2 g of silica, and then dry loaded onto a silica column packed as a slurry in hexanes. Elution with 3:7 CH_2_Cl_2_:hexanes moved a green band overlapped with a red band, which were collected in small test tube fractions. Over the course of a slow evaporation over 2 days, black crystals formed on the walls of the test tubes. Following identification of these crystals as **2**·2^1^/_2_CH_2_Cl_2_ by X-ray crystallography (JPD Data Set 1395), they were collected, washed with copious amounts of Et_2_O and vacuum-dried. Yield: 0.0418 g, 19%. *R*_f_ = 0.58 (2:3 CH_2_Cl_2_:petroleum ether). ^1^H NMR (δ, ppm in CD_2_Cl_2_): 7.49–7.47 (dm, 4 H, phenyl C–*H*), 7.32–7.26 (m, 9 H, phenyl C–*H*), 7.25–7.20 (m, 11 H, phenyl C–*H*), 7.19–7.15 (m, 4 H, phenyl C–*H*), 7.15–7.08 (m, 24 H, phenyl C–*H*), 7.03–6.95 (m, 8 H, phenyl C–*H*). ^13^C NMR (δ, ppm in CD_2_Cl_2_): 152.33, 148.98, 148.51, 144.81, 144.69, 140.79, 139.72, 139.55, 139.45, 139.33, 138.87, 138.71, 138.54, 138.16, 137.89, 130.39, 130.32, 130.08, 130.02, 129.93, 129.42, 128.52, 128.43, 128.26, 128.12, 128.02, 127.99, 127.95, 127.93, 127.86, 127.84, 127.67, 127.62, 120.47. UV-vis [CH_2_Cl_2_, λ_max_, nm (ε_M_, M^–1^ cm^–1^)]: 230 (20400), 250 (19700), 370 (6800), ∼445 (sh, ∼3000). MS (MALDI^+^) Calcd for C_84_H_60_S_12_Pd_6_: *m*/*z* 2092.6200; Observed: *m*/*z* 2092.6477; Error (δ) 13.24 ppm. Cyclic voltammetry (CH_2_Cl_2_, [^*n*^Bu_4_N]­[PF_6_] supporting electrolyte, [Cp_2_Fe]^+^/Cp_2_Fe reference): **2** – e^–^ → [**2**]^+^, +0.835 V; **2** + e^–^ → [**2**]^−^, −1.137 V; [**2**]^−^ + e^–^ → [**2**]^2–^, −1.589 V.

### [Pt_6_(S_2_C_2_Ph_2_)_6_] (**3**)

An oven-dried 100 mL Schlenk flask with a stir bar was charged with [Pt­(S_2_C_2_Ph_2_)_2_] (0.3361 g, 0.494 mmol), Pt_2_(dba)_3_ (0.9757 g, 0.893 mmol), and toluene (30 mL) under an atmosphere of N_2_. This mixture reaction was heated at 90 °C for 72 h, at which point it was cooled to ambient temperature and taken to dryness under reduced pressure. This crude solid was redissolved in CH_2_Cl_2_, evaporated onto ∼2 g of silica, and dry loaded onto a silica column packed as a slurry in hexanes. Elution with 3:7 CH_2_Cl_2_:hexanes moved **3** as the leading orange band, which was collected and reduced to dryness. This solid was dissolved in a minimal volume of nitrobenzene, passed through a packed Celite pad, and slowly evaporated to form red-black crystals of **3**·7PhNO_2_, the formulation of which was established by an X-ray crystal structure (JPD Data set 1322). Yield: 0.0368 g, 0.0140 mmol, 8.5%. *R*_f_ = 0.57 (2:3 CH_2_Cl_2_:petroleum ether). ^1^H NMR (CD_2_Cl_2_): δ 7.54–7.52 (m, 4 H, phenyl C–*H*), 7.41–7.39 (m, 8 H, phenyl C–*H*), 7.32–7.26 (m, 5 H, phenyl C–*H*), 7.25–7.18 (m, 18 H, phenyl C–*H*), 7.16–7.10 (m, 17 H, phenyl C–*H*), 7.04–6.99 (m, 8 H, phenyl C–*H*). UV–vis [CH_2_Cl_2_, λ_max_, nm (ε_M_, M^–1^ cm^–1^)]: 255 (13100), 305 (7600), 350 (3400). MS (MALDI+) Calcd for C_84_H_60_S_12_Pt_6_: *m*/*z* 2624.6226; Observed: *m*/*z* 2624.9820; Error (δ): 137 ppm. Cyclic voltammetry (CH_2_Cl_2_, [^*n*^Bu_4_N]­[PF_6_] supporting electrolyte, [Cp_2_Fe]^+^/Cp_2_Fe reference): **3** – e^–^ → [**3**]^+^, +0.738 V; [**3**]^**+**^ – e^–^ → [**3**]^2+^, +1.076 V; **3** + e^–^ → [**3**]^–^, −1.662 V; [**3**]^–^ + e^–^ → [**3**]^2–^, −2.055 V. At temperatures above 50 °C in air, thermal decomposition of **3** was observed.

## Results and Discussion

### Syntheses and Structures

The synthesis of the [M_6_(S_2_C_2_Ph_2_)_6_] cage compounds may be executed by any one of the several distinct approaches illustrated in Scheme 1. Method A, as a redox formalism, entails the partitioning of a [M­(S_2_C_2_Ph_2_)_2_] complex into [(Ph_2_C_2_S_2_^2–^)­M^2+^] and α-dithione fragments, the former of which aggregate to produce [M_6_(S_2_C_2_Ph_2_)_6_] and the latter of which oxidatively adds to a zerovalent metal source such as [Mo­(CO)_3_(MeCN)_3_] or Fe­(CO)_5_. Although it does not conform to the specific, narrow criteria that are usually associated with simple oxidative addition reactions, e.g., an increase of two in the coordination number of a monometallic complex,^20^ Method B involves an oxidative association of 3 equiv of zerovalent M with 3 [M­(S_2_C_2_Ph_2_)_2_] such that all metal atoms become divalent ions. The six electrons extracted from M^0^ to produce M^2+^ are delivered to the dithiolene radical monoanions in [M­(S_2_C_2_Ph_2_)_2_] (Scheme 1), thereby transforming them to fully reduced ene-1,2-dithiolates. Powdered metal, employed in excess, is effective for nickel, but for Pd and Pt, the soluble dimetal tris­(dibenzylideneacetone) complexes (M_2_(dba)_3_) are more viable, intrinsically reactive sources of the zerovalent metal. For M = Pd, exchange of chloride for dithiolene via the agency of a dialkyl tin dithiolene reagent transiently produces [(Ph_2_C_2_S_2_)­Pd­(MeCN)_2_] (Method C), which presumably self-assembles into the compact hexametallic structure because the thiolate-type sulfur, even in a bridging mode, is a more strongly binding ligand than acetonitrile. Yields are modest and progressively diminish as Group 10 is descended: 37% for **1**, 17–19% for **2**, 9% for **3**.

**Scheme 1:**
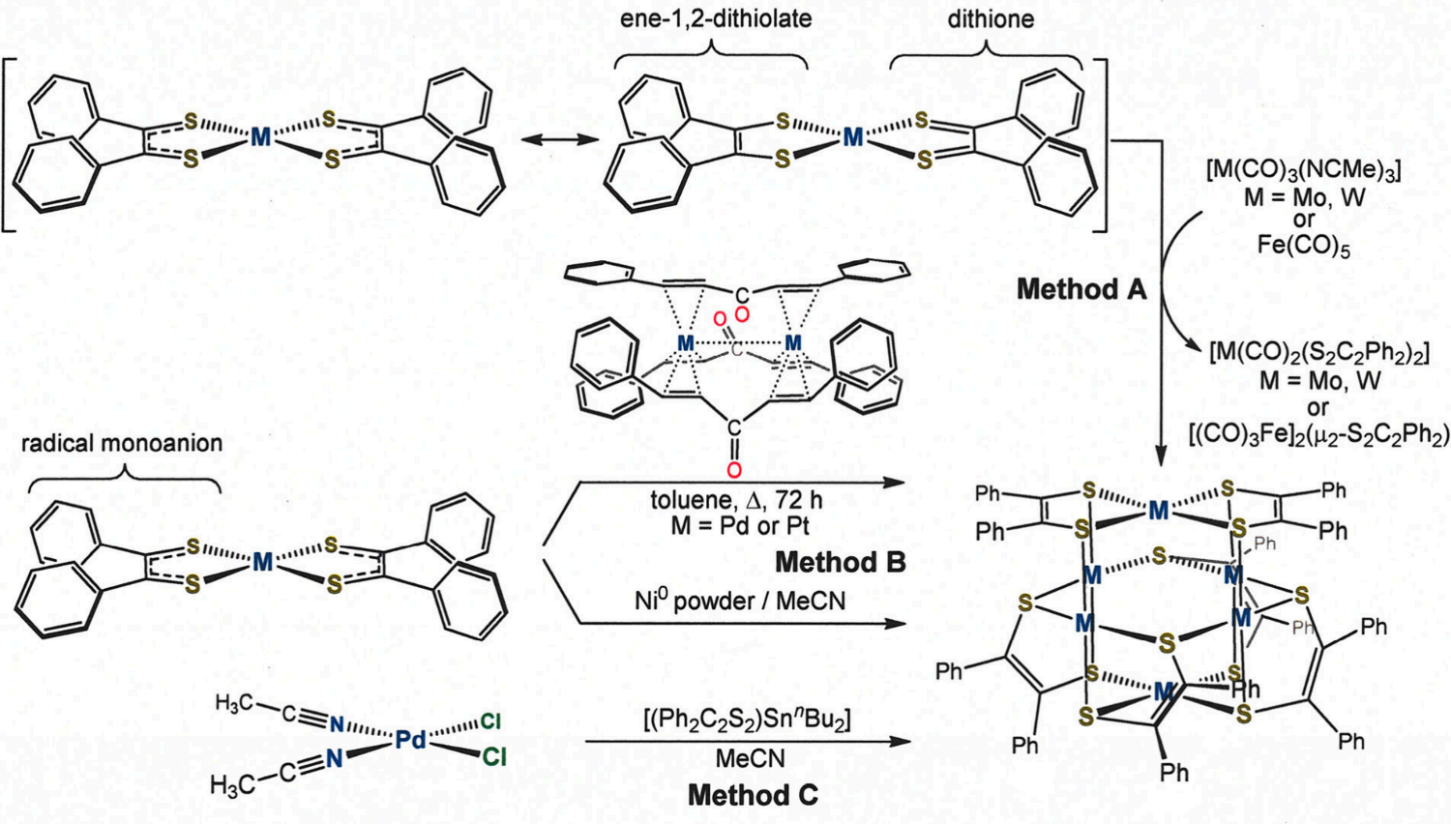
Synthetic Approaches to [M_6_(S_2_C_2_Ph_2_)_6_] Cage Compounds

The greatest reproducibility in crystallizing compounds **1**–**3** was found by slow evaporation from the PhNO_2_ solutions. Closeness in the dimensions of two unit-cell axes (Table 1) initially led to the assumption of a tetragonal crystal system in the search for the space group. While no tractable solution was attainable in a tetragonal space group, solutions and satisfactory refinements in all cases were achieved in orthorhombic *Pbca* (No. 61), even though differing numbers of interstitial PhNO_2_ molecules (seven for **1** and **3**, one for **2**) co-crystallized with these metallodithiolene cages. In the case of **3**·7PhNO_2_, data of appreciably better quality were obtained at 248 K rather than at the more typical 150 K, due to an apparent phase change that was either incomplete or too quickly induced. Unit-cell data, refinement statistics, and other selected crystallographic data are summarized in Table 1.

**Table 1:** Unit-Cell and Refinement Data for [M_6_(S_2_C_2_Ph_2_)_6_]^*n*^ (M = Ni, Pd, Pt, *n* = 0; M = Ni, *n* = −1)

compound	[Ni_6_(S_2_C_2_Ph_2_)_6_]	[Pd_6_(S_2_C_2_Ph_2_)_6_]	[Pt_6_(S_2_C_2_Ph_2_)_6_]	[Cp*_2_Co] [Ni_6_(S_2_C_2_Ph_2_)_6_]
formula	C_126_H_95_N_7_Ni_6_O_14_S_12_	C_86.5_H_65_Cl_5_Pd_6_S_12_	C_126_H_95_N_7_O_14_Pt_6_S_12_	C_109_H_100.50_CoNNi_6_O_0.75_S_12_
fw	2668.06	2304.75	3486.30	2232.31
temperature, K	125	150	248	159
wavelength, Å	0.71073	0.71073	0.71073	0.71073
2θ range, deg	3.650–56.874	4.352–52.964	3.908–48.568	3.852–66.576
crystal system	orthorhombic	orthorhombic	orthorhombic	monoclinic
space group	*Pbca*	*Pbca*	*Pbca*	*C*2/*c*
*a*, Å	26.373(2)	25.3863(17)	26.818(3)	25.9512(8)
*b*, Å	26.580(2)	26.3255(17)	26.851(3)	18.3809(6)
*c*, Å	32.760(3)	26.6307(17)	33.139(3)	43.0630(12)
α, deg	90	90	90	90
β, deg	90	90	90	94.065(1)
γ, deg	90	90	90	90
volume, Å^3^	22965(3)	17797(2)	23863(4)	20489.6(11)
*Z*	8	8	8	8
crystal size	0.154 × 0.212 × 0.316	0.087 × 0.127 × 0.308	0.030 × 0.031 × 0.481	0.055 × 0.172 × 0.347
color, habit	brown block	black block	brown-black plate	black plate
indep. data	28 827	18 353	19 245	39 394
param. refnd	1482	996	1454	1186
goodness of fit, GooF^a^	1.105	1.082	1.187	1.035
*R*_1_,^b^ ^,^ ^c^ *wR*_2_ ^d^ ^,^ ^c^	0.0768, 0.2056	0.0626, 0.1644	0.0318, 0.0621	0.0458, 0.1027
*R*_1_,^b^ ^,^ ^e^ *wR*_2_ ^d^ ^,^ ^e^	0.1255, 0.2445	0.1201, 0.2099	0.0484, 0.0727	0.0861, 0.1218

a GooF = {∑[*w*(*F*_0_^2^ – *F*_c_^2^)^2^]/(*n* – *p*)}^1/2^, where *n* = number of reflections and *p* is the total number of parameters refined.

b *R*_1_ = ∑||*F*_0_| – |*F*_c_||/∑|*F*_0_|.

c *R* indices for data cut off at *I* > 2σ­(*I*).

d *wR*_2_ = {∑[*w*(*F*_0_^2^ – *F*_c_^2^)^2^]/∑[*w*(*F*_0_^2^)^2^]}^1/2^; *w* = 1/[σ^2^(*F*_0_^2^) + (*xP*)^2^ + *yP*], where *P* = (2*F*_c_^2^ + Max­(*F*_0_^2^,0)/3.

e *R* indices for all data.

The structures of **1**–**3** feature an octahedral arrangement of six divalent Group 10 metal ions with the four coordinate square planar geometry around each ion that is typical of the d^8^ electron configuration. Each of the 12 edges of the M_6_ octahedron is bridged by a dithiolene sulfur atom (Figure 1d). As demonstrated rigorously by Fekl and co-workers,^15^ the joining of pairs of adjacent sulfur atoms by the chelating olefinic backbone of a dithiolene ligand reduces the core *O*_*h*_ symmetry to either idealized centrosymmetric *S*_6_ symmetry (Figure 1e) or chiral *C*_2_ symmetry (Figure 1f). The higher symmetry *S*_6_ structure is distinguished by the requirement that all six M^2+^ ions be in identical environments such that each can be exchanged with all others by successive executions of the *S*_6_ operation. This condition is possible only if each M^2+^ ion has a single chelating [Ph_2_C_2_S_2_]^2–^ ligand, with which it forms a near planar MS_2_C_2_ ring, and bridging interactions with two different dithiolene ligands, whose S_2_C_2_ mean planes each approach orthogonality to the MS_4_ interior plane. The lower symmetry *C*_2_ structure is marked by its incorporation of one M^2+^ ion that is fully chelated by two dithiolene ligands (vide infra) and one M^2+^ ion that has no five atom MS_2_C_2_ chelate ring.

The *C*_2_ isomer is the sole isomer observed in this study, irrespective of the use of polar (MeCN) or nonpolar solvent (toluene) in the synthesis, suggesting that solvent polarity is not a property of the reaction medium that influences the outcome. The computed dipole moment for **1** that accompanies its tight geometry optimization is a somewhat surprising 5.1 debye, but the greater contribution to this value is likely the physical separation between charges rather than the magnitude of the charges. The calculated electrostatic potential map for **1** (Figure 2a) reveals no clear charge separation beyond what occurs between the center and the perimeter of the individual phenyl rings. If the phenyl rings of **1** are substituted with H atoms, structural optimization produces a dipole moment that has lesser magnitude (3.6 debye) but is much more discernible in an electrostatic potential map (Figure 2b). The Ph groups around the periphery of **1** greatly obscure the intrinsic dipole moment that it actually possesses.

**Figure 2:**
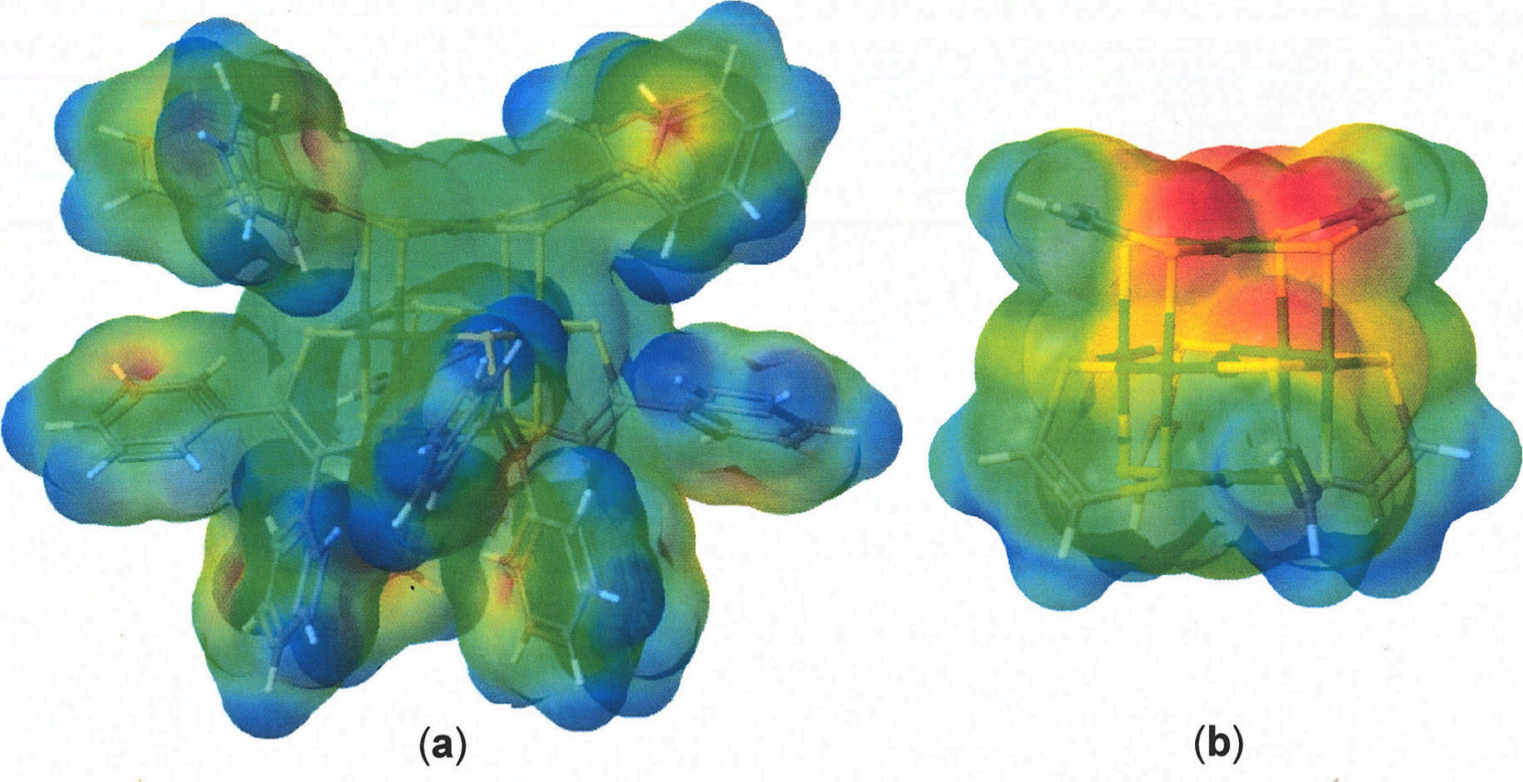
(a) Calculated electrostatic potential map for **1** viewed with the same orientation as the structures in Figure 3. The isosurface data range is −0.1176 to 0.1054. (b) Calculated electrostatic potential map for *C*_2_-symmetric [Ni_6_(S_2_C_2_H_2_)_6_] with isosurface data ranging from 0.2814 to 0.4157.

A tight geometry optimization of **2** as the *S*_6_ isomer readily proceeds when substituting the −C­(O)­OCH_3_ groups for Ph in the crystallographic coordinates for [Pd_6_(S_2_C_2_(C­(O)­OCH_3_)_2_)_6_]. A marginal 0.40 kcal/mol difference favors the *S*_6_ isomer of **2**, indicating that the isomers are effectively isoenergetic. A modestly greater 1.60 kcal/mol energy difference appears between the *C*_2_ and *S*_6_ isomers of [Pd_6_(S_2_C_2_(C­(O)­OH)_2_)_6_], which was used as a surrogate for [Pd_6_(S_2_C_2_(C­(O)­OCH_3_)_2_)_6_], because of its greater tractability in converging to energy minima. Again, the *S*_6_ structure, the isomer observed by Stiefel for [Pd_6_(S_2_C_2_(C­(O)­OCH_3_)_2_)_6_], is computed as the lower energy species. Assuming these computations are qualitatively correct, the final energy separation between isomers is still too slight to decisively preclude the formation of a mixture.

The consistent observation of the *C*_2_ forms of **1**–**3** in this work has its probable origin in a statistical bias rather than thermodynamic favorability. As detailed in the analysis by Fekl,^15^ a beginning point of a single chelated “M­(S_2_C_2_)” fragment and systemic mapping of all possible pathways reveals *C*_2_:*S*_6_ outcomes in a 3:1 ratio. A further amplification of the *C*_2_ isomer in the product distribution could also arise from the particular method of synthesis. The choice of square planar [M­(S_2_C_2_Ph_2_)_2_] as a starting point (Methods A and B, Scheme 1) may template the structural assembly toward the *C*_2_ isomer, which incorporates an intact square planar [M­(S_2_C_2_Ph_2_)_2_] fragment. Conversely, the transmetalation approach (Method C, Scheme 1) through the agency of either a Sn or Zn dithiolene complex, presumably forms a transient “M­(S_2_C_2_Ph_2_)” fragment which then faces, at 25% probability, its best chance of ending in the *S*_6_ isomer.

The foregoing interpretation of the synthetic outcome is indirectly corroborated by an NMR spectroscopic evaluation of the crude product for **1**. Following column chromatography purification and isolation of **1** as a single band, but prior to crystallization, both ^1^H and ^13^C NMR spectral analyses reveal well-resolved minor signals in addition to those attributable to the pure *C*_2_ isomer when single crystals are used to compose the sample solution (Figures S38–S41). The *S*_6_ isomer is therefore likely present in minor amounts, unresolved from the *C*_2_ isomer by chromatography but excluded during crystal growth.

Deconstruction of the *C*_2_-symmetric structures of **1**–**3** shows they are comprised of an idealized *D*_2*h*_ symmetric “planar” M­(S_2_C_2_Ph_2_)_2_ top (blue, in Figure 3a) set upon a chiral *C*_4_ base formed by a group of four M­(S_2_C_2_Ph_2_) fragments (red and green, Figure 3a) that twist around a single divalent M^2+^ ion at the very bottom of the assembly (green, Figure 3a). While the metal ion at the top of the structure (blue, in Figure 3a) is fully chelated by two dithiolene ligands, the M^2+^ ion at the base distinctly differs from the others in having no chelating dithiolene ligand but only dithiolene sulfur atoms bridged to it from the central belt of four M­(S_2_C_2_Ph_2_) groups. The thermal ellipsoid plot of **3**, presented in Figure 3b, is representative of the set.

**Figure 3:**
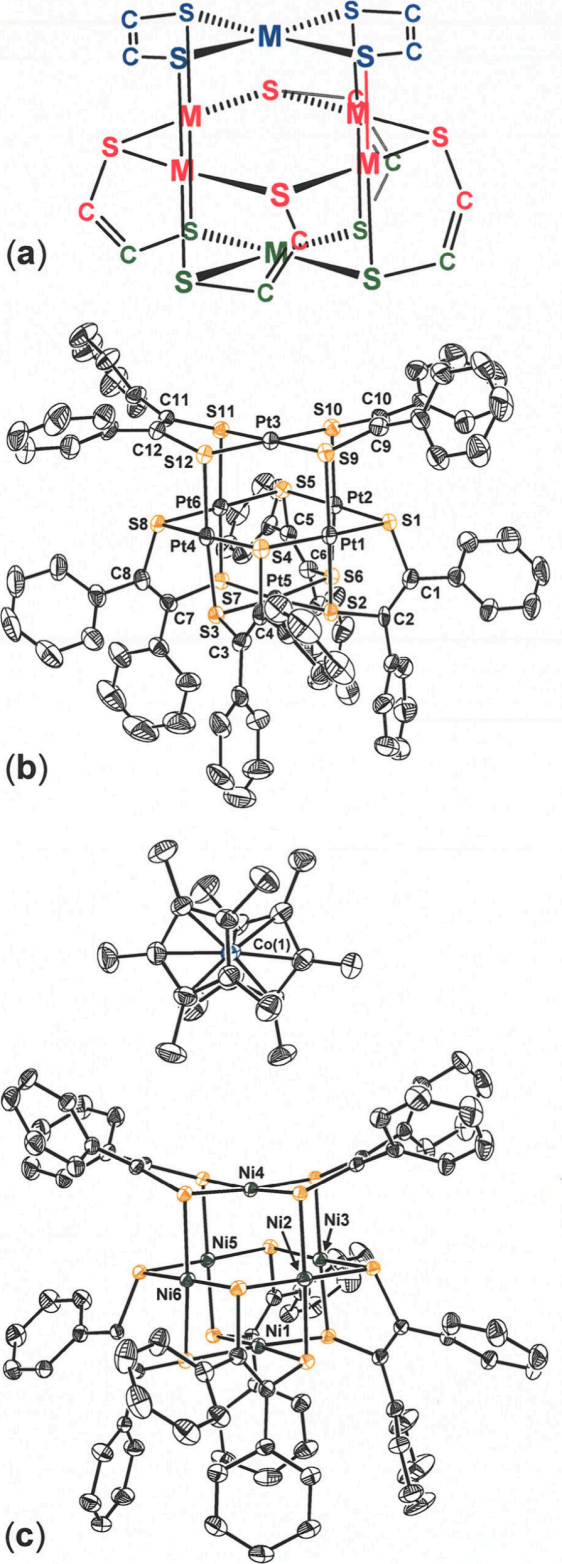
(a) Color coding of fragments of [M_6_(S_2_C_2_)_6_] for reference to the interatomic distances and angles in Table 2 and (b, c) thermal ellipsoid plots of [Pt_6_(S_2_C_2_Ph_2_)_6_] (panel (b)) and [Cp*_2_Co]­[Ni_6_(S_2_C_2_Ph_2_)_6_] (panel (c)) at the 50% probability level. All H atoms have been omitted for the sake of clarity.

Despite the lack of symmetry relating the top portion of the assembly to the bottom, nonbonding intermetal distances vary only modestly from those of a core M_6_ octahedron. Opposite M**···**M distances are quite similar at ∼4.38 Å (Table 2); adjacent M**···**M distances in the central belt (red) alternate as ∼3.01 and ∼3.19 Å. Metal–sulfur bond lengths are ∼0.05–0.06 Å shorter for the chelating interactions (e.g., Pt3–S9, Figure 3b, Table 2) as compared to the bridging interactions (e.g., Pt2–S1, Figure 3b, Table 2). The S–C and C–C intrachelate bond lengths (∼1.78 and ∼1.35 Å, respectively) are consistent with a fully reduced ene-1,2-dithiolate dianion. An upward fold along the intraligand S**···**S axis of each dithiolene ligand in the capping M­(S_2_C_2_Ph_2_)_2_ fragment (blue), such that the angle between S_2_C_2_ planes is ∼40°, confers a saddlelike character to this fragment of the structure. Additional interatomic distances and angles are summarized, as averages where possible, in Table S2.

**Table 2:** Selected Interatomic Distances (Å) and Angles (deg) for [M_6_(S_2_C_2_Ph_2_)_6_]^*n*^ ^a^ ^,^ ^b^

	M = Ni, *n* = 0	M = Ni, *n* = −1	M = Pd, *n* = 0^c^	M = Pt, *n* = 0
M_blue_–S_blue_	2.1847[8]	2.1855[3]	2.305[1]	2.2999[9]
M_red_–S_red,long_	2.2504[8]	2.2684[3]	2.369[1]	2.3631[9[
M_red_–S_red,short_	2.1767[8]	2.1874[3]	2.297[1]	2.2901[9]
M_green_–S_green_	2.2366[8]	2.3111[3]	2.351[1]	2.3472[9]
M_red_–S_blue_	2.2442[8]	2.2579[3]	2.362[1]	2.3597[9]
M_red_–S_green_	2.1808[8]	2.1773[3]	2.290[1]	2.2939[9]
S_blue_–C_blue_	1.775[3]	1.775[1]	1.790[5]	1.795[4]
S_red_–C_red_	1.779[3]	1.774[1]	1.787[5]	1.792[4]
S_green_–C_green_	1.780[3]	1.767[1]	1.775[4]	1.795[4]
C_blue_=C_blue_	1.358[6]	1.349[2]	1.348[9]	1.353[7]
C_red_=C_green_	1.359[4]	1.351[2]	1.353[6]	1.336[5]
M_blue_···M_green_	4.3811(11)	4.2763(4)	4.7049(10)	4.7652(6)
M_blue_···M_red_	3.0858[6]	3.0762[2]	3.2965[5]	3.3588[2]
M_red_···M_green_	3.1125[5]	3.0612[2]	3.3345[5]	3.3731[2]
M_red_···M_red,cis,short_	3.0100[7]	3.0452[3]	3.2375[6]	3.2630[3]
M_red_···M_red,cis,long_	3.1883[7]	3.1384[3]	3.3693[6]	3.4592[4]
M_red_···M_red,trans_	4.3846[8]	4.3708[3]	4.6725[6]	4.7552[4]
δ_M,blue_ ^d^	0.056	0.074	0.0285	0.0113
δ_M,red_	0.026	0.042	0.0002	0.0437
δ_M,green_	0.015	0.048	0.0739	0.0837
θ^e^	43.57(14)	47.10(6)	37.8(2)	38.5(2)

a Chemically equivalent interatomic distances and angles are averaged. For averaged values, uncertainties are determined according to the general formula for error propagation, as described by Taylor,^21^ and are enclosed in square brackets.

b The subscripted colors following atoms M, S, and C in the leftmost column designate specific interatomic distances or displacements as defined in Figure 3a.

c Values are for [Pd_6_(S_2_C_2_Ph_2_)_6_]·2^1^/_2_CH_2_Cl_2_ rather than [Pd_6_(S_2_C_2_Ph_2_)_6_]·PhNO_2_.

d δ = Absolute value of displacement (Å) of M from S_4_M mean plane.

e θ = angle between the S_2_C_2_ mean planes in blue fragment (Figure 3a).

Electron transfer to **1** by the outer sphere reductant [Cp*_2_Co] provides [Cp*_2_Co]­[**1**], which is readily crystallized from MeCN/Et_2_O. As suggested by the highly reversible nature of the cathodic waves in the cyclic voltammogram (vide infra), [Cp*_2_Co]­[**1**] is isostructural with **1** and differs chiefly by moderate lengthening (∼0.07 Å) of the Ni–S bond lengths at the base of the *C*_4_ fragment (green, Table 2), a lessening in the magnitude of alternation of Ni**···**Ni distances in the central belt (0.093 Å in [**1**]^1–^ vs 0.178 Å in **1**), and increased displacement of the Ni ions from the NiS_4_ mean planes (Table 2). A crystal structure of [Ph_4_P]­[Pd_6_(S_2_C_2_(CO_2_Me)_2_)_6_], deposited in the CSD without preparative detail or commentary on its differences with its neutral counterpart, is the sole other example of characterization of the anion type.^22^

### Electrochemistry, Electronic Structure, and Spectroscopy

Cyclic voltammograms for **1**–**3** in CH_2_Cl_2_ are presented in Figure 4, with *E*_1/2_ potential values collected in Table 3. From a starting rest potential of ∼0.5 V vs [Cp_2_Fe]^+^/Cp_2_Fe, cathodic scanning reveals two reversible reductions for each compound that correspond to the generation of monoanionic and then dianionic [M_6_(S_2_C_2_Ph_2_)_6_]^*n*−^. The potentials at which these reductions occur are quite similar for **1** and **2** (Table 3), but potentials that are appreciably more negative by ∼0.5 V must be applied to induce the same electron-transfer reactions for **3**. A ∼0.25 eV greater HOMO–LUMO gap that is calculated for **3** vs **1** is consistent with this observation (cf. Figure 5, as well as Figures S42 and S43). In the anodic direction, **1** and **2** share the common denominator of a reversible oxidation to the monocation, but a potential that is substantially more positive by +0.44 V is demanded for **2**. In the potential window that is accessible in CH_2_Cl_2_, **3** supports two reversible oxidations in relatively close succession at +0.74 and +1.08 V. The wide potential window separating the first oxidation and first reduction in these compounds, a window which exceeds 2 V in the case of **3**, underscores the intrinsic redox stability of charge neutral **1**–**3**. The differences among **1**–**3**, both in the number of reversible processes and in the pronounced shifts in potentials for analogous processes, contrast with the [M­(S_2_C_2_Ph_2_)_2_] (M = Ni, Pd, Pt) series (Figure S35, Table 3), the members of which are much more similar than different.

**Figure 4:**
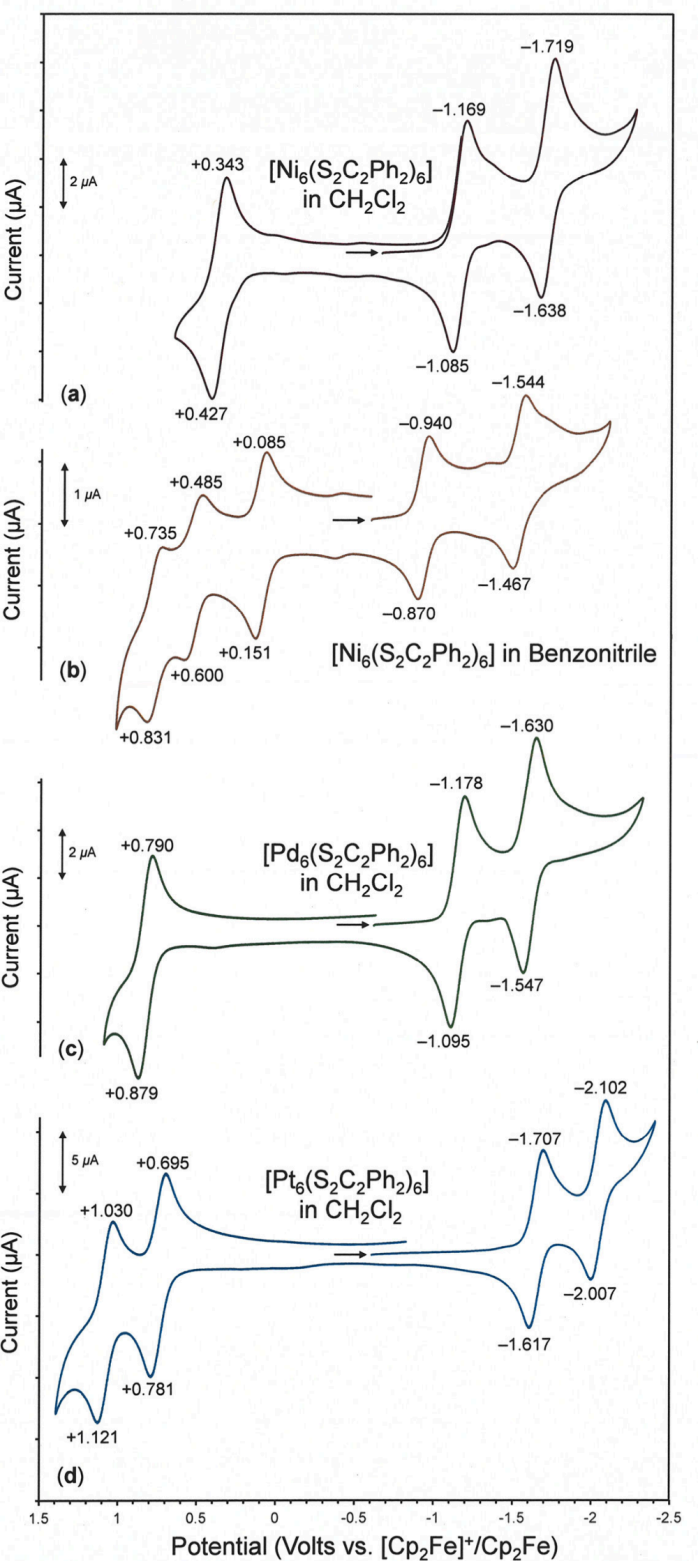
Cyclic voltammetry (CV) of (a) [Ni_6_(S_2_C_2_Ph_2_)_6_], **1**, (b) [Pd_6_(S_2_C_2_Ph_2_)_6_], **2**, (c) and [Pt_6_(S_2_C_2_Ph_2_)_6_], **3**, (**d**) in CH_2_Cl_2_ with [^*n*^Bu_4_N]­[PF_6_] supporting electrolyte and Pt disk working electrode. The CV of [Ni_6_(S_2_C_2_Ph_2_)_6_] in benzonitrile is shown in panel (**b**). Anodic and cathodic current maxima are indicated in volts, and the initial potential for scanning is marked by the right arrow.

**Table 3:** Potential (V vs [Cp_2_Fe]^+^/Cp_2_Fe) for Electron Transfers^a^ by **1**–**3** and [M­(S_2_C_2_Ph_2_)_2_] (M = Ni, Pd, Pt)

	[M_6_]^2+^ – e^–^ → [M_6_]^3+^	[M_6_]^1+^ – e^–^ → [M_6_]^2+^	[M_6_] – e^–^ → [M_6_]^1+^	[M_6_] + e^–^ → [M_6_]^−^	[M_6_]^−^ + e^–^ → [M_6_]^2–^
**1** ^b^	–	–	+0.39	–1.13	–1.68
**1** ^c^	+0.78	+0.54	+0.12	–0.91	–1.51
**2** ^b^	–	–	+0.83	–1.14	–1.59
**3** ^b^	–	+1.08	+0.74	–1.66	–2.05

	[M(pdt)_2_] – e^–^ → [M(pdt)_2_]^+^	[M(pdt)_2_] + e^–^ → [M(pdt)_2_]^−^	[M(pdt)_2_]^−^ + e^–^ → [M(pdt)_2_]^2–^
[Ni(pdt)_2_]^b^ ^,^ ^d^	+0.75^e^	–0.43	–1.25
[Pd(pdt)_2_]^b^	+0.66^e^	–0.43	–1.08
[Pt(pdt)_2_]^b^	+0.74	–0.50	–1.24

a *E*_c_ and *E*_a_ values for each wave are shown in Figure 4; data were obtained with [^*n*^Bu_4_N]­[PF_6_] supporting electrolyte and Pt disk working electrode.

b Measurement in CH_2_Cl_2_.

c Measurement in benzonitrile with [^*n*^Bu_4_N]­[PF_6_] supporting electrolyte and Pt disk working electrode.

d pdt = phenyldithiolene = [S_2_C_2_Ph_2_]^*n*^.

e Quasi­reversible.

**Figure 5:**
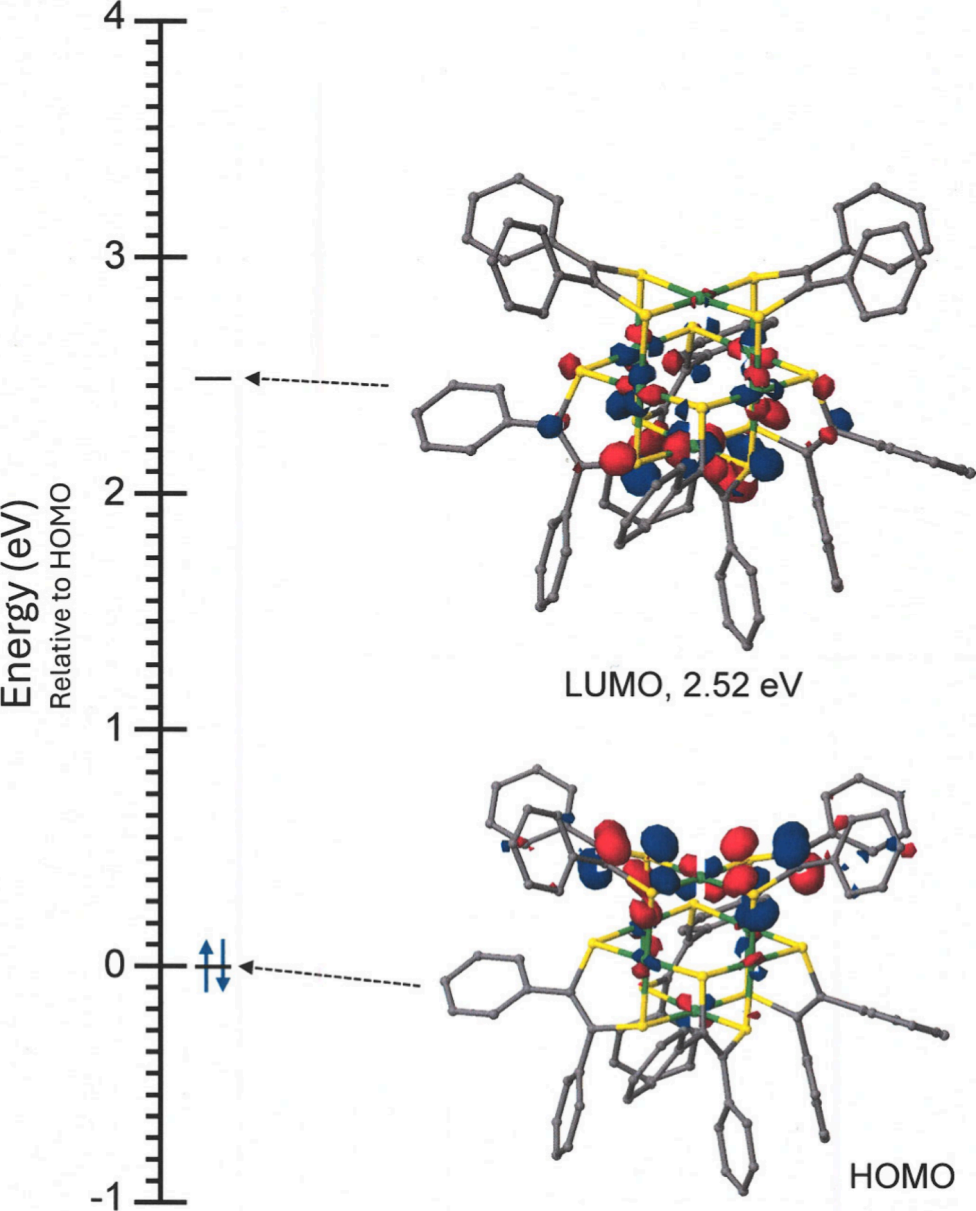
MO energy level diagram for [Ni_6_(S_2_C_2_Ph_2_)_6_]. The orbital images are rendered at the 0.05 contour level.

Considering both the fully reduced character of the six Ph_2_C_2_S_2_^2–^ ligands in **1**–**3** and the well-known description of the electron-transfer reactions that interrelate [Ni­(S_2_C_2_R_2_)_2_]^2–^ complexes with their neutral form as being ene-1,2-dithiolate-to-radical-monoanion processes,^23^ a greater number of reversible oxidations were anticipated for **1** and **2** than the single one observed. Solvent effects, however, can be significant in their ability to shift redox potentials or stabilize an otherwise irreversible process. In benzonitrile, chosen initially for its capacity to support oxidative scanning to a potential ∼0.5 V more positive than is feasible in CH_2_Cl_2_, **1** reveals *three* reversible—or nearly reversible—oxidations in close succession, the first of which occurs at a potential that is 0.27 V less positive than the single oxidation observed in CH_2_Cl_2_ (Figure 4b, Table 3). The composition of the HOMO for **1** (Figure 5), which is qualitatively quite similar to the HOMO for [Ni­(S_2_C_2_Ph_2_)_2_]^2–^, is largely of dithiolene C_2_S_2_ p π character at the [Ni­(S_2_C_2_Ph_2_)_2_] fragment (blue fragment, Figure 3a) that caps the *C*_4_ base (red and green, Figure 3a). The planar and exposed nature of this MO intuitively suggests that it would be stabilized by π–π interactions with an arene solvent. However, under the same conditions, neither **2** nor **3** revealed the reversible oxidations in benzonitrile that are found for **1**.

The UV-vis spectrum for **1** reveals a broad maximum at 445 nm, a rather narrower one at 350 nm and a shoulder at 270 nm, but the absorption profile is marked by multiple, unresolved swells and shoulders occurring between these features and tapering down to ∼700 nm, which intimate the presence of numerous electronic excitations along this energy continuum (Figure 6). The high density of both occupied and unoccupied states that is implied by this profile is typical of metal clusters and other multimetal compounds and contrasts with the relative simplicity of the electronic absorption spectra of related monometallic compounds. The spectra for **2** and **3** are qualitative similar to that of **1** but are shifted progressively to higher energy such that **3** reveals no resolved maximum along its more compressed window of visible absorption. This pattern in isostructural compounds for electronic absorptions to shift to higher energy as the group is traversed to heavier transition elements is general^24^ and attributed to an increasing strength of metal ligand bonding that produces bonding interactions of lower energy and antibonding interactions, which typically constitute the acceptor MOs, that are pushed to intrinsically higher energy.

**Figure 6:**
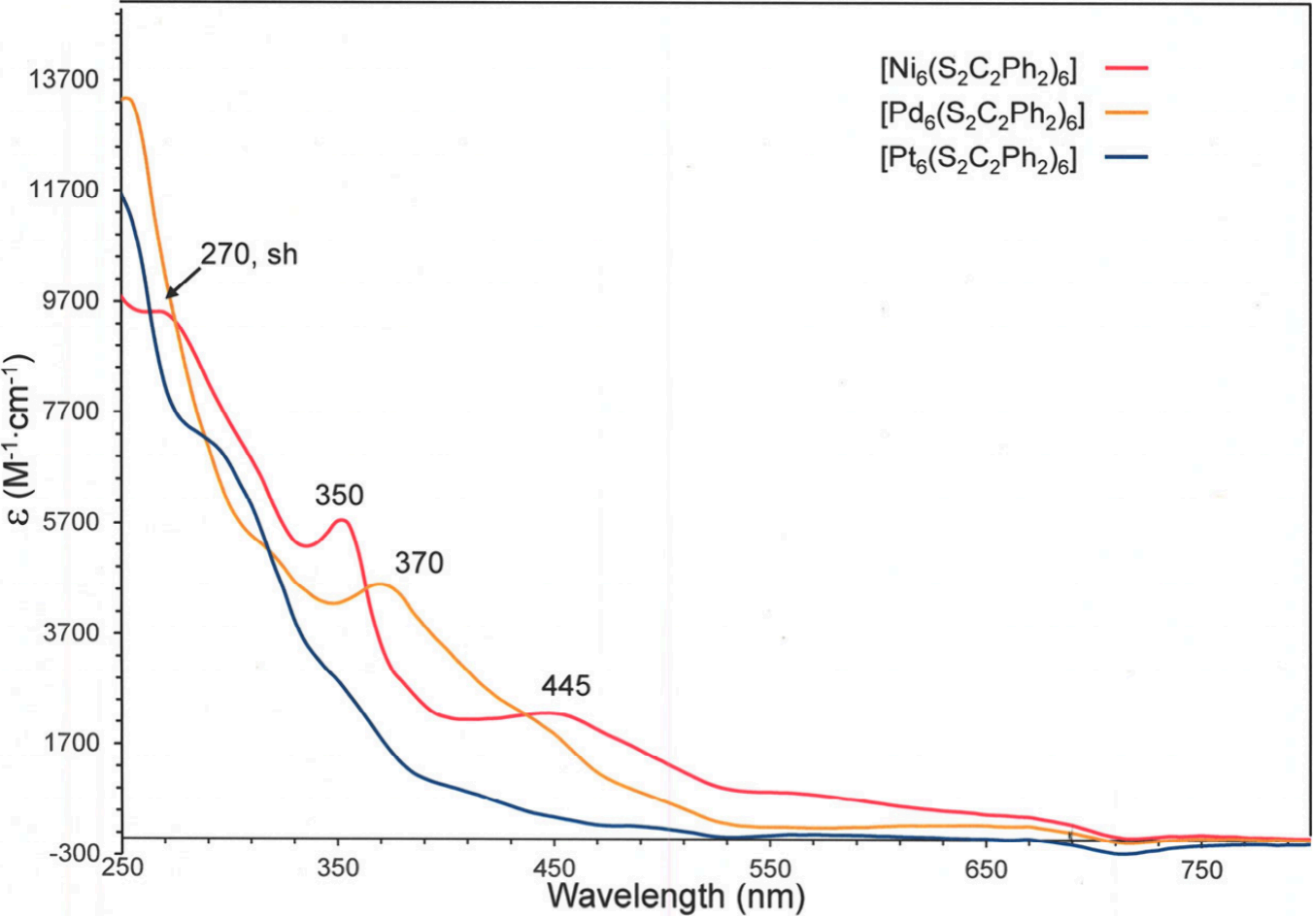
Overlaid UV-vis spectra (CH_2_Cl_2_) for [Ni_6_(S_2_C_2_Ph_2_)_6_] (red trace), [Pd_6_(S_2_C_2_Ph_2_)_6_] (orange trace), and [Pt_6_(S_2_C_2_Ph_2_)_6_] (blue trace).

Simulation of the UV–vis spectra for **1**–**3** in CH_2_Cl_2_ reveals a multitude of excitations contributing to the observed absorption profiles (Tables S9–S11). Taking **2** as representative of the set, all members of whom have general qualitative similarity, the most significant excitations on the basis of oscillator strength are the HOMO → LUMO, HOMO–1 → LUMO+1, and HOMO–1 → LUMO+2 transitions (Figure S44). These excitations share an essentially similar character as mixed metal–ligand-to-metal charge-transfer transitions.

Spectroscopic monitoring of **1** in an OTTLE-type cell poised at −1.2 V (versus Ag^+^/Ag) reveals a decrease in intensity of the absorption maxima at 445 and 350 nm accompanied by a comparable increase in intensity between these features. The effect of these changes, which are fully reversible upon application of a −0.4 V potential to the electrode, is a smoothing of the absorption profile to a featureless slope (Figure S45). The EPR spectrum of [**1**]^1–^ (Figure S46) reveals the broad isotropic signal that is anticipated for a spin delocalized around the Ni_5_(S_2_C_2_)_4_ core that comprises the *C*_4_ base of the assembly (cf. Figure 5). On the anodic side, oxidation of **1** at a poised potential of +1.2 V was attended by a reversible diminution of the intensity of the absorption at 350 nm but an irreversible shift to higher intensity of the baseline at the higher energy side of the 350 nm feature (Figure S47).

Spectroelectrochemical changes similar to, but somewhat more modest than, those of **1** attend the reduction of **2** when it is held at a potential of −0.94 V. Diminishing intensity for the maximum at 370 nm and increasing absorption intensity in the ∼475–550 nm region combine to produce a near-featureless spectrum (Figure S48). Little further change occurs when [**2**]^2–^ is generated at −1.3 V vs Ag^+^/Ag beyond a modest increase in absorption in the ∼550–625 nm window (Figure S49). The in-situ generation of [**2**]^+^ at 1.7 V is marked by no discernible change at all in the 300–800 nm region (Figure S50). The trend toward lessened spectroelectrochemical change continues with **3**, which reveals only marginal change upon generation of [**3**]^1–^ and no perceptible differences when any of its other redox states are accessed at the appropriate potentials (Figures S51–S54). Although shifts in the energies of the frontier MOs necessarily occur upon change in charge state, the multitude of overlapping transitions, both before and after, appear to make the absorption profile of **3** insensitive to the redox state.

## Conclusion/Summary

This report provides a complete series of hexametallic [M_6_(S_2_C_2_Ph_2_)_6_] (M = Ni^2+^, Pd^2+^, Pt^2+^) dithiolene complexes, a class of metallodithiolene complexes that is unique to the Group 10 transition metals and distinguished by a 1:1 ratio of ligand-to-metal. Our principal findings are as follows:

(1) The air- and moisture-stable [M_6_(S_2_C_2_Ph_2_)_6_] compounds are accessible through several distinct routes: Reaction of 3 equiv of a M^0^ source with 3 equiv [M­(S_2_C_2_Ph_2_)_2_], aggregation of “M­(S_2_C_2_Ph_2_)” formed by dithioketone transfer from [M­(S_2_C_2_Ph_2_)_2_], and condensation of a presumed [(Ph_2_C_2_S_2_)­M­(MeCN)_2_] intermediate formed by dithiolene transfer to [Cl_2_M­(MeCN)_2_].
(2) X-ray crystallographic characterization of crystalline samples obtained from slowly evaporated PhNO_2_ solutions reveals a common, *C*_2_-symmetric cage-like structure formed by an octahedron of nonbonding M^2+^ ions, each of the 12 edges of which are bridged by a dithiolene sulfur atom. The crystal structures confirm that the ene-1,2-dithiolate dianion is the correct redox description dithiolene ligands.
(3) Both reversible oxidations and reversible reductions are accessible by cyclic voltammetry in CH_2_Cl_2_, the latter of which are necessarily metal-based processes in view of the fully reduced state of the ligands. The oxidations are ligand-based and localized to a unique M­(S_2_C_2_Ph_2_)_2_ fragment that caps the assembly. In benzonitrile, **1** supports three-reversible oxidations with an appreciable cathodic shift in contrast to the single oxidation that is accessible in CH_2_Cl_2_.
(4) The UV-vis spectra are complex and, as a set, are chiefly marked by (i) unresolved shoulders and ridges along the absorption profile and (ii) a shift to higher energy in moving from Ni to Pt.

Although the attainable quantities of **2** and **3** are limited, the better yield for **1** is substantial enough to permit investigation of such further questions as its suitability to serve as a precursor to new heteroleptic dithiolene compounds not accessible by other routes and the degree to which the magnitude of the HOMO–LUMO gap may be tailored by an appropriate choice of aryl substituent.

## Supplementary Material



## Notes

Procedures for crystal growth, X-ray diffraction data collection, structure solution and refinement, and DFT-computational work; table summaries of unit-cell and refinement data (Table S1) and of selected averaged interatomic distances and angles (Table S2); thermal ellipsoid plots with complete atom labeling (Figures S1–S18); spectroscopic, spectrometric, and analytical data; MO energy level diagrams for **2** and **3** (Figures S19–S54); final atomic coordinates for geometry-optimized structures (Tables S3–S8); calculated electronic transitions (Tables S9–S11) (PDF)
