# Supplementary material for: The [M6(S2C2Ph2)6] (M = Ni, Pd, Pt) Series: Multielectron Reservoirs That Sustain Ligand-Based Oxidations and Metal-Based Reductions
Source: Inorg Chem. 2025 Nov 24;64(48):23446–56. doi: 10.1021/acs.inorgchem.5c03536 (PMC12690577; doi:10.1021/acs.inorgchem.5c03536)
Supplement: Supplementary file 1 [file ic5c03536_si_001.pdf]

Supporting Information

The $[M_6(S_2C_2Ph_2)_6]$ (M = Ni, Pd, Pt) Series: Multi-Electron Reservoirs that Sustain Ligand-Based Oxidations and Metal-Based Reductions

by

Titir Das Gupta,[§] Brian Wuille-Bille,[†] Olaf Ruediger,[†] Xiaodong Zhang,[§] Joel T. Mague,[§] Serena DeBeer,[†] and James P. Donahue[§,*]

[§]Department of Chemistry, Tulane University, 6400 Freret St., New Orleans, Louisiana 70118, United States.
[†]Max-Planck-Institut für Chemische Energiekonversion, Stiftstrasse 34-36, D-45470 Mülheim an der Ruhr, Germany.
[*]Author to whom correspondence may be addressed: donahue@tulane.edu.

Table of Contents

Procedures for Crystal Growth, Collection and Processing of Diffraction Data, and Solving and Refining of Structures S4-S5
Other Physical Methods S5
Computational Details S5
References S6
Table S1. Structure and refinement data for $[Ni_6(S_2C_2Ph_2)_6]\cdot7PhNO_2$, $[Pd_6(S_2C_2Ph_2)_6]\cdot PhNO_2$, $[Pd_6(S_2C_2Ph_2)_6]\cdot2\frac{1}{2}CH_2Cl_2$, $[Pt_6(S_2C_2Ph_2)_6]\cdot7PhNO_2$, and $[Cp^*{}_2Co][Ni_6(S_2C_2Ph_2)_6]\cdot0.75Et_2O\cdot MeCN$. S7
Table S2. Selected interatomic distances and angles and other structural data for $[Ni_6(S_2C_2Ph_2)_6]\cdot7PhNO_2$, $[Pd_6(S_2C_2Ph_2)_6]\cdot2PhNO_2$, $[Pd_6(S_2C_2Ph_2)_6]\cdot2\frac{1}{2}CH_2Cl_2$, $[Pt_6(S_2C_2Ph_2)_6]\cdot7PhNO_2$, and $[Cp^*{}_2Co][Ni_6(S_2C_2Ph_2)_6]\cdot0.75Et_2O\cdot MeCN$. S8
Figure S1. Thermal ellipsoid plot of $[Ni_6(S_2C_2Ph_2)_6]$ with complete atom labeling. S9
Figure S2. Thermal ellipsoid plot of $[Ni_6(S_2C_2Ph_2)_6]$ with partial atom labeling. S10
Figure S3. Thermal ellipsoid plot with atom labeling of interior core of $[Ni_6(S_2C_2Ph_2)_6]$. S11
Figure S4. Thermal ellipsoid plot with atom labeling for co-crystallized $PhNO_2$ in $[Ni_6(S_2C_2Ph_2)_6]\cdot7(PhNO_2)$. S12
Figure S5. Thermal ellipsoid plot of $[Pd_6(S_2C_2Ph_2)_6]$ in $[Pd_6(S_2C_2Ph_2)_6]\cdot PhNO_2$ with complete atom labeling. S13
Figure S6. Thermal ellipsoid plot of $[Pd_6(S_2C_2Ph_2)_6]$ in $[Pd_6(S_2C_2Ph_2)_6]\cdot PhNO_2$ with partial atom labeling. S14
Figure S7. Thermal ellipsoid plot with atom labeling of interior core of $[Pd_6(S_2C_2Ph_2)_6]$. S15
Figure S8. Thermal ellipsoid plot with atom labeling for co-crystallized $PhNO_2$ in $[Pd_6(S_2C_2Ph_2)_6]\cdot(PhNO_2)$. S16
Figure S9. Thermal ellipsoid plot of $[Pd_6(S_2C_2Ph_2)_6]$ in $[Pd_6(S_2C_2Ph_2)_6]\cdot2\frac{1}{2}CH_2Cl_2$ with complete atom labeling. S17
Figure S10. Thermal ellipsoid plot with atom labeling for co-crystallized CH_2Cl_2 in $[Pd_6(S_2C_2Ph_2)_6]\cdot2\frac{1}{2}CH_2Cl_2$. S18
Figure S11. Thermal ellipsoid plot of $[Pt_6(S_2C_2Ph_2)_6]$ with complete atom labeling. S19
Figure S12. Thermal ellipsoid plot of $[Pt_6(S_2C_2Ph_2)_6]$ with partial atom labeling. S20
Figure S13. Thermal ellipsoid plot with atom labeling of interior core of $[Pt_6(S_2C_2Ph_2)_6]$. S21
Figure S14. Thermal ellipsoid plot with atom labeling for co-crystallized $PhNO_2$ in $[Pt_6(S_2C_2Ph_2)_6]\cdot7(PhNO_2)$. S22
Figure S15. Thermal ellipsoid plot of $[Ni_6(S_2C_2Ph_2)_6]^{1-}$ with full atom labeling. S23
Figure S16. Thermal ellipsoid plot of $[Ni_6(S_2C_2Ph_2)_6]^{1-}$, atom labeling for core atoms. S24
Figure S17. Thermal ellipsoid plot of $[((CH_3)_5C_5)_2Co][Ni_6(S_2C_2Ph_2)_6]$ with atom labeling for the countercation. S25
Figure S18. Thermal ellipsoid plot with atom labeling for the interstitial solvent in $[((CH_3)_5C_5)_2Co][Ni_6(S_2C_2Ph_2)_6]\cdot MeCN\cdot\frac{3}{4}Et_2O$. S26
Figure S19. ^1H NMR spectrum of $[Ni_6(S_2C_2Ph_2)_6]$ in CD_2Cl_2, 0.0 - 10.0 ppm. S27
Figure S20. ^1H NMR spectrum of $[Ni_6(S_2C_2Ph_2)_6]$ in CD_2Cl_2, 6.85 - 7.60 ppm. S28
Figure S21. ^{13}C NMR spectrum of $[Ni_6(S_2C_2Ph_2)_6]$ in CD_2Cl_2, –10.0 - 215.0 ppm. S29
Figure S22. ^{13}C NMR spectrum of $[Ni_6(S_2C_2Ph_2)_6]$ in CD_2Cl_2, 123.0 - 150.0 ppm. S30
Figure S23. ^1H NMR spectrum of $[Pd_6(S_2C_2Ph_2)_6]$ in CD_2Cl_2, 0.0 - 10.0 ppm. S31
Figure S24. ^1H NMR spectrum of $[Pd_6(S_2C_2Ph_2)_6]$ in CD_2Cl_2, 6.93 - 7.55 ppm. S32

Table of Contents, Continued

**Figure S25**.	^{13}C NMR spectrum of $[Pd_6(S_2C_2Ph_2)_6]$ in CD_2Cl_2, 0.0 - 215.0 ppm.	S33
**Figure S26**.	^{13}C NMR spectrum of $[Pd_6(S_2C_2Ph_2)_6]$ in CD_2Cl_2, 115.0 - 160.0 ppm.	S34
**Figure S27.**	^{1}H NMR spectrum of $[Pt_6(S_2C_2Ph_2)_6]$ in CD_2Cl_2, 0.0 - 10.0 ppm.	S35
**Figure S28**.	^{1}H NMR spectrum of $[Pt_6(S_2C_2Ph_2)_6]$ in CD_2Cl_2, 6.94 - 7.58 ppm.	S36
**Figure S29**.	UV-vis spectrum of $[Ni_6(S_2C_2Ph_2)_6]$ in CH_2Cl_2.	S37
**Figure S30**.	UV-vis spectrum of $[Pd_6(S_2C_2Ph_2)_6]$ in CH_2Cl_2.	S38
**Figure S31**.	UV-vis spectrum of $[Pt_6(S_2C_2Ph_2)_6]$ in CH_2Cl_2.	S39
**Figure S32**.	ESI mass spectrum for $[Ni_6(S_2C_2Ph_2)_6]$.	S40
**Figure S33**.	MALDI mass spectrum for $[Pd_6(S_2C_2Ph_2)_6]$ with simulated spectrum.	S41
**Figure S34**.	MALDI mass spectrum for $[Pt_6(S_2C_2Ph_2)_6]$ with simulated spectrum.	S42
**Figure S35.**	CV (CH_2Cl_2) for $[Ni(S_2C_2Ph_2)_2]$, $[Pd(S_2C_2Ph_2)_2]$ and $[Pt(S_2C_2Ph_2)_2]$.	S43
**Figure S36**.	Elemental analysis request form for $[Ni_6(S_2C_2Ph_2)_6]$.	S44
**Figure S37**.	Elemental analysis results for $[Ni_6(S_2C_2Ph_2)_6]$.	S45
**Figure S38**.	^{1}H NMR spectrum (CD_2Cl_2) of chromatographed but uncrystallized $[Ni_6(S_2C_2Ph_2)_6]$.	S46
**Figure S39**.	Comparison of the ^{1}H NMR spectrum (CD_2Cl_2) of column chromatographed but uncrystallized $[Ni_6(S_2C_2Ph_2)_6]$ against that of crystallized $[Ni_6(S_2C_2Ph_2)_6]$.	S47
**Figure S40**.	Comparison of the ^{13}C NMR spectrum (CD_2Cl_2) of column chromatographed but uncrystallized $[Ni_6(S_2C_2Ph_2)_6]$ against that of crystallized $[Ni_6(S_2C_2Ph_2)_6]$ in the 122-148 ppm region.	S48
**Figure S41**.	Comparison of ^{13}C NMR spectrum (CD_2Cl_2) of chromatographed but uncry-stallized $[Ni_6(S_2C_2Ph_2)_6]$ against that of crystallized sample, 125.4-131.5 ppm.	S49
**Figure S42**.	MO energy level diagram for $[Pd_6(S_2C_2Ph_2)_6]$.	S50
**Figure S43**.	MO Energy level diagram for $[Pt_6(S_2C_2Ph_2)_6]$.	S51
**Figure S44**.	Illustration of the most intense electronic excitations for $[Pd_6(S_2C_2Ph_2)_6]$.	S52
**Figure S45**.	UV-vis spectrum of $[Ni_6(S_2C_2Ph_2)_6]^{1-}$, generated spectroelectrochemically.	S53
**Figure S46**.	EPR spectrum (CH_2Cl_2) of $[Ni_6(S_2C_2Ph_2)_6]^{1-}$.	S54
**Figure S47**.	UV-vis spectrum of $[Ni_6(S_2C_2Ph_2)_6]^{1+}$, generated spectroelectrochemically.	S55
**Figure S48**.	UV-vis spectrum of $[Pd_6(S_2C_2Ph_2)_6]^{1-}$, generated spectroelectrochemically.	S56
**Figure S49**.	UV-vis spectrum of $[Pd_6(S_2C_2Ph_2)_6]^{2-}$, generated spectroelectrochemically.	S56
**Figure S50**.	UV-vis spectrum of $[Pd_6(S_2C_2Ph_2)_6]^{1+}$, generated spectroelectrochemically.	S57
**Figure S51**.	UV-vis spectrum of $[Pt_6(S_2C_2Ph_2)_6]^{1-}$, generated spectroelectrochemically.	S58
**Figure S52**.	UV-vis spectrum of $[Pt_6(S_2C_2Ph_2)_6]^{2-}$, generated spectroelectrochemically.	S58
**Figure S53**.	UV-vis spectrum of $[Pt_6(S_2C_2Ph_2)_6]^{1+}$, generated spectroelectrochemically.	S59
**Figure S54**.	UV-vis spectrum of $[Pt_6(S_2C_2Ph_2)_6]^{2+}$, generated spectroelectrochemically.	S59
**Table S3**.	Final Atomic Coordinates in Geometry-Optimized C_2 $[Ni_6(S_2C_2Ph_2)_6]$.	S60-S63
**Table S4**.	Final Atomic Coordinates in Geometry-Optimized C_2 $[Pd_6(S_2C_2Ph_2)_6]$.	S64-S67
**Table S5**.	Final Atomic Coordinates in Geometry-Optimized S_6 $[Pd_6(S_2C_2Ph_2)_6]$.	S68-S71
**Table S6**.	Final Atomic Coordinates in Geometry-Optimized C_2 $[Pt_6(S_2C_2Ph_2)_6]$.	S72-S75
**Table S7**.	Final Atomic Coordinates, Geometry-Optimized C_2 $[Pd_6(S_2C_2(CO_2H)_2)_6]$.	S76-S77
**Table S8**.	Final Atomic Coordinates, Geometry-Optimized S_6 $[Pd_6(S_2C_2(CO_2H)_2)_6]$.	S78-S79
**Table S9**.	Calculated Electronic Transitions for $[Ni_6(S_2C_2Ph_2)_6]$.	S80-S84
**Table S10**.	Calculated Electronic Transitions for $[Pd_6(S_2C_2Ph_2)_6]$.	S85-S91
**Table S11**.	Calculated Electronic Transitions for $[Pt_6(S_2C_2Ph_2)_6]$.	S92-S98

Procedures for Crystal Growth, Collection and Processing of Diffraction Data, and Solving and Refining of Structures.

The crystalline samples of $[Ni(S_2C_2Ph_2)]_6{\cdot}7PhNO_2$ (brown blocks), $[Pd(S_2C_2Ph_2)]_6{\cdot}PhNO_2$ (brown-black plates), and $[PtS_2C_2Ph_2)]_6{\cdot}7PhNO_2$ (red needles) used for the collection of X-ray diffraction data were obtained by slow evaporation of nitrobenzene solutions in the open air. Black, block-shaped crystals of $[Pd(S_2C_2Ph_2)]_6{\cdot}2½CH_2Cl_2$ were obtained by evaporation of a test-tube fraction from a silica chromatography column that was eluted with 3:7 CH_2Cl_2:hexanes, while black plate crystals of $[(Me_5C_5)_2Co][Ni(S_2C_2Ph_2)]_6{\cdot}MeCN{\cdot}0.75Et_2O$ were grown by the diffusion of Et_2O vapor into an MeCN solution.

All crystals were coated with paratone oil and mounted on the end of a nylon loop attached to the end of the goniometer. Data were collected at 125 K for $[NiS_2C_2Ph_2)]_6{\cdot}7PhNO_2$, 150 K for $[Pd(S_2C_2Ph_2)]_6{\cdot}PhNO_2$ and $[Pd(S_2C_2Ph_2)]_6{\cdot}2½CH_2Cl_2$, 248 K (for $[PtS_2C_2Ph_2)]_6{\cdot}7PhNO_2$) and 159 K for $[(Me_5C_5)_2Co][Ni(S_2C_2Ph_2)]_6{\cdot}MeCN{\cdot}0.75Et_2O$ under a dry N_2 stream supplied under the control of an Oxford Cryostream 800 attachment. The somewhat higher temperature for $[PtS_2C_2Ph_2)]_6{\cdot}7PhNO_2$ was used because, at lower temperatures, the crystals appeared to undergo a monoclinic-to-tetragonal phase change that complicated identification of the correct space group. The data collection instrument was Bruker D8 Quest Photon 3 diffractometer equipped with a Mo fine-focus sealed tube providing radiation at $\lambda = 0.71073$ nm. The numbers of frames and the frame times used for the data sets acquired for the crystal structures reported here were as follows: $[NiS_2C_2Ph_2)]_6{\cdot}7PhNO_2$: 16 sets of either 437 or 720 frames at 20 seconds/frame; $[Pd(S_2C_2Ph_2)]_6{\cdot}PhNO_2$: 5 sets of either 407 or 721 frames at 20 seconds/frame; $[Pd(S_2C_2Ph_2)]_6{\cdot}2½CH_2Cl_2$: 7 sets of 397 frames at 30 seconds/frame; $[PtS_2C_2Ph_2)]_6{\cdot}7PhNO_2$: 10 sets of either 407 or 720 frames at 15 sec/frame; $[(Me_5C_5)_2Co][Ni(S_2C_2Ph_2)]_6{\cdot}MeCN{\cdot}0.75Et_2O$: 10 sets of 440 frames at 20 sec/frame; the particular parameters of these data collections were chosen by the "strategy" routine within/*APEX*.[1]

Raw data were reduced to F^2 values using *SAINT*,[2] and a global refinement of unit cell parameters was performed using ~9100–9900 selected reflections from the full data sets. All data sets were corrected for absorption on the basis of multiple measurements of symmetry equivalent reflections or by numerical methods with the use of *SADABS*,[3] as described by Krause *et al.*[4] All structure solutions were obtained by direct methods using *SHELXT*,[5] while refinements were accomplished by full-matrix least-squares procedures using *SHELXL*.[6] The *SHELXL* program is

incorporated into both the *SHELXTL*[7] and *APEX*[1] software suites. In most respects, refinement was routine. Static disorder in two of the interstitial CH_2Cl_2 molecules of $[Pd(S_2C_2Ph_2)]_6{\cdot}2½CH_2Cl_2$ and the interstitial Et_2O molecule in $[(Me_5C_5)_2Co][Ni(S_2C_2Ph_2)]_6{\cdot}MeCN{\cdot}0.75Et_2O$ was addressed using a split atom model with floating site occupancies whose values were determined as a best fit by the refinement software. All H atoms were added in calculated positions with isotropic displacement parameters 1.2 - 1.5 times those of the carbon atoms to which they were attached. All images were created using the graphics program *XP*, which is a routine contained within *SHELXTL*. All structures were checked for overlooked symmetry and other errors by the checkCIF service provided by the International Union of Crystallography.[8]

Other Physical Methods

X-band cw EPR spectra of the reduction products for **1** were obtained using an X-band Bruker Elexsys EPR spectrometer equipped with a ER4116DM dual mode resonator and an ESR 900 He cryostat. The bulk electrolysis for **1** was performed in an H-shaped electrochemical cell separated by a porous glass frit. After current stabilization, catholyte samples were transferred to EPR tubes and frozen in liquid N_2. Their spectra were acquired at 20 K.

Computational Details

Density Functional Theory calculations were done by using the High Performance Computing facility- Cypress at Tulane University using Gaussian-09 package.[9] Geometry optimizations used the coordinates of the X-ray crystallographic data of the neutral molecules and implemented the Becke, three parameter Lee-Yang- Parr (B3LYP) level of theory.[10,11] The 6-31G(d,p) basis set was used for carbon atoms, the TZVP basis set was used for sulfur atoms, and a Gaussian split-valence (SV) basis set[12] was used for hydrogen atoms. For nickel, palladium and platinum, a triple-ζ (TZ) basis set with effective core potential (LANL2TZ ECP) was utilized.[13] The molecular orbital (MO) plots and electrostatic potential map were created using the Jmol program package.[14]

References

(**1**) (**a**) *APEX2*, Bruker-AXS, Inc., Madison, Wisconsin, USA, 2015. (**b**) *APEX3*, Bruker-AXS, Inc., Madison, Wisconsin, USA, 2020. (**c**) *APEX4*, Bruker-AXS, Inc., Madison, Wisconsin, USA, 2021.

(**2**) (a) *SAINT*, Bruker AXS, Inc., Madison, Wisconsin, 2015. (b) *SAINT*, Bruker AXS, Inc., Madison, Wisconsin, 2020. (c) *SAINT*, Bruker AXS, Inc., Madison, Wisconsin, 2021.

(**3**) *SADABS*, Bruker AXS, Inc., Madison, Wisconsin, 2016.

(**4**) Krause, L.; Herbst-Irmer, R.; Sheldrick, G.M.; Stalke; D. Comparison of Silver and Molybdenum Microfocus X-ray Sources for Single-Crystal Structure Determination. *J. Appl. Cryst.* **2015**, *48*, 3-10.

(**5**) Sheldrick, G. M. *SHELXT* – Integrated Space-Group and Crystal-Structure Determination. *Acta Crystallogr., Sect A* **2015**, *71*, 3-8.

(**6**) (**a**) Sheldrick, G. M. A Short History of *SHELX*. *Acta Crystallogr., Sect. A* **2008**, *64*, 112-122. (**b**) Sheldrick, G. M. *SHELXL-2018/1*. University of Göttingen, Göttingen, Germany, 2018.

(**7**) (**a**) *SHELXTL*, Bruker-AXS, Madison, WI, 2015. (**b**) *SHELXTL*, Bruker-AXS, Madison, WI, 2020. (**c**) *SHELXTL*, Bruker-AXS, Madison, WI, 2021.

(**8**) See http://checkcif.iucr.org/

(**9**) Frisch, M. J.; Trucks, G. W.; Schlegel, H. B.; Scuseria, G. E.; Robb, M. A.; Cheeseman, J. R.; Scalmani, G.; Barone, V.; Petersson, G. A.; Nakatsuji, H.; Li, X.; Caricato, M.; Marenich, A. V.; Bloino, J.; Janesko, B. G.; Gomperts, R.; Mennucci, B.; Hratchian, H. P.; Ortiz, J. V.; Izmaylov, A. F.; Sonnenberg, J. L.; Williams; Ding, F.; Lipparini, F.; Egidi, F.; Goings, J.; Peng, B.; Petrone, A.; Henderson, T.; Ranasinghe, D.; Zakrzewski, V. G.; Gao, J.; Rega, N.; Zheng, G.; Liang, W.; Hada, M.; Ehara, M.; Toyota, K.; Fukuda, R.; Hasegawa, J.; Ishida, M.; Nakajima, T.; Honda, Y.; Kitao, O.; Nakai, H.; Vreven, T.; Throssell, K.; Montgomery, Jr., J. A.; Peralta, J. E.; Ogliaro, F.; Bearpark, M. J.; Heyd, J. J.; Brothers, E. N.; Kudin, K. N.; Staroverov, V. N.; Keith, T. A.; Kobayashi, R.; Normand, J.; Raghavachari, K.; Rendell, A. P.; Burant, J. C.; Iyengar, S. S.; Tomasi, J.; Cossi, M.; Millam, J. M.; Klene, M.; Adamo, C.; Cammi, R.; Ochterski, J. W.; Martin, R. L.; Morokuma, K.; Farkas, O.; Foresman, J. B.; Fox, D. J. Gaussian 16 Rev. C.01, Wallingford, CT, 2016.

(**10**) Becke, A., Thermo chemistry Density-Functional III. The Role of Exact Exchange. *J. Chem. Phys.* **1993**, *98* (7), 5648-5652.

(**11**) Lee, C.; Yang, W.; Parr, R. G., Development of the Colle-Salvetti Correlation-Energy Formula into a Functional of the Electron Density. *Phys. Rev. B* **1988**, *37* (2), 785.

(**12**) Schäfer, A.; Horn, H.; Ahlrichs, R., Fully Optimized Contracted Gaussian Basis Sets for Atoms Li to Kr. *J. Chem. Phys.* **1992**, *97* (4), 2571-2577.

(**13**) https://www.basissetexchange.org/

(**14**) Jmol: an open-source Java viewer for chemical structures in 3D.

(**15**) Taylor, J. R. *An Introduction to Error Analysis*, 2nd ed.; University Science Books: Sausalito, CA, 1997; pp 73-77.

Table S1. Unit Cell and Refinement Data for $[M_6(S_2C_2Ph_2)_6]^n$ (M = Ni, Pd, Pt; $n = 0$ and M = Ni, $n = -1$).

compound	$[Ni_6(S_2C_2Ph_2)_6]$	$[Pd_6(S_2C_2Ph_2)_6]$	$[Pd_6(S_2C_2Ph_2)_6]$	$[Pt_6(S_2C_2Ph_2)_6]$	$[Cp^*_2Co]$ $[Ni_6(S_2C_2Ph_2)_6]$
struct. code	JPD1236	JPD1216	JPD1395	JPD1322	JPD1483
solvent	7 $PhNO_2$	$PhNO_2$	2½CH_2Cl_2	7 $PhNO_2$	MeCN·¾Et_2O
formula	$C_{126}H_{95}N_7Ni_6O_{14}S_{12}$	$C_{90}H_{60}NO_2Pd_6S_{12}$	$C_{86.50}H_{65}Cl_5Pd_6S_{12}$	$C_{126}H_{95}N_7O_{14}Pt_6S_{12}$	$C_{109}H_{100.50}CoNNi_6O_{0.75}S_{12}$
fw	2668.06	2210.51	2304.75	3486.30	2232.31
temperature, K	125	150	150	248	159
wavelength, Å	0.71073	0.71073	0.71073	0.71073	0.71073
2θ range, deg.	3.650 – 56.874	3.442 – 50.290	4.352 – 52.964	3.908 – 48.568	3.852 – 66.576
crystal system	orthorhombic	orthorhombic	orthorhombic	orthorhombic	monoclinic
space grp	*Pbca*	*Pbca*	*Pbca*	*Pbca*	*C*2/*c*
a, Å	26.373(2)	25.948(3)	25.3863(17)	26.818(3)	25.9512(8)
b, Å	26.580(2)	26.427(3)	26.3255(17)	26.851(3)	18.3809(6)
c, Å	32.760(3)	26.598(3)	26.6307(17)	33.139(3)	43.0630(12)
α, deg.	90	90	90	90	90
β, deg.	90	90	90	90	94.065(1)
γ, deg.	90	90	90	90	90
volume, $Å^3$	22965(3)	18239(3)	17797(2)	23863(4)	20489.6(11)
Z	8	8	8	8	8
density, g/cm^3	1.543	1.610	1.720	1.941	1.447
μ, mm^{-1}	1.251	1.478	1.662	7.284	1.530
F(000)	10976	8728	9096	13376	9236
crystal size	0.154x0.212x0.316	0.033x0.117x0.270	0.087x0.127x0.308	0.030x0.031x0.481	0.055 x 0.172 x 0.347
color, habit	brown block	brown-black plate	black block	red needle	black plate
limit. indices, *h*	$-35 \le h \le 35$	$-30 \le h \le 30$	$-31 \le h \le 31$	$-30 \le h \le 30$	$-40 \le h \le 40$
limit. indices, *k*	$-35 \le k \le 35$	$-31 \le k \le 31$	$-32 \le k \le 32$	$-30 \le k \le 30$	$-28 \le k \le 28$
limit. indices, *l*	$-43 \le l \le 43$	$-31 \le l \le 31$	$-33 \le l \le 33$	$-38 \le l \le 38$	$-66 \le l \le 66$
reflns collected	1367582	331064	498526	856735	478088
indep. data	28827	15850	18353	19245	39394
restraints	12	6	1042	2378	3
param. refnd	1482	806	996	1454	1186
GooF[a]	1.105	1.095	1.082	1.187	1.035
R1,[b,c] wR2[d,c]	0.0768, 0.2056	0.1393, 0.3716	0.0626, 0.1644	0.0318, 0.0621	0.0458, 0.1027
R1,[b,e] wR2[d,e]	0.1255, 0.2445	01842, 0.4061	0.1201, 0.2099	0.0484, 0.0727	0.0861, 0.1218
larg. peak, e·$Å^{-3}$	4.486	4.824	4.976	1.067	2.280
larg. hole, e·$Å^{-3}$	–1.219	–2.638	–1.457	–0.916	–0.788

[a]GooF = $\{\Sigma[w(F_o^2 - F_c^2)^2]/(n - p)\}^{½}$, where n= number of reflections and p is the total number of parameters refined; [b]R1 = $\Sigma||F_o| - |F_c||/\Sigma|F_o|$; [c]R indices for data cut off at I > 2σ(I); [d]wR2 = $\{\Sigma[w(F_o^2 - F_c^2)^2]/\Sigma w(F_o^2)^2\}^{1/2}$; $w = 1/[\sigma^2(F_o^2) + (xP)^2 + yP]$, where $P = (F_o^2 + 2F_c^2)/3$; [e]R indices for all data.

Table S2. Selected interatomic distances (Å) and angles (deg.) for $[M_6(S_2C_2Ph_2)_6]^n$.[a,b]

	**M = Ni, *n* = 0**	**M = Ni, *n* = 1-**	**M = Pd, *n* = 0**[c]	**M = Pd, *n* = 0**[d]	**M = Pt, *n* = 0**
M_{blue}–S_{blue}	2.1847[8]	2.1855[3]	2.301[3]	2.305[1]	2.2999[9]
M_{red}–$S_{red,long}$	2.2504[8]	2.2684[3]	2.356[3]	2.369[1]	2.3631[9[
M_{red}–$S_{red,short}$	2.1767[8]	2.1874[3]	2.299[3]	2.297[1]	2.2901[9]
M_{green}–S_{green}	2.2366[8]	2.3111[3]	2.355[3]	2.351[1]	2.3472[9]
M_{red}–S_{blue}	2.2442[8]	2.2579[3]	2.359[3]	2.362[1]	2.3597[9]
M_{red}–S_{green}	2.1808[8]	2.1773[3]	2.298[3]	2.290[1]	2.2939[9]
S_{blue}–C_{blue}	1.775[3]	1.775[1]	1.78[1]	1.790[5]	1.795[4]
S_{red}–C_{red}	1.779[3]	1.774[1]	1.81[2]	1.787[5]	1.792[4]
S_{green}–C_{green}	1.780[3]	1.767[1]	1.79[1]	1.775[4]	1.795[4]
C_{blue}=C_{blue}	1.358[6]	1.349[2]	1.32[3]	1.348[9]	1.353[7]
C_{red}=C_{green}	1.359[4]	1.351[2]	1.33[2]	1.353[6]	1.336[5]
$M_{blue}\cdots M_{green}$	4.3811(11)	4.2763(4)	4.6775(26)	4.7049(10)	4.7652(6)
$M_{blue}\cdots M_{red}$	3.0858[6]	3.0762[2]	3.2948[12]	3.2965[5]	3.3588[2]
$M_{red}\cdots M_{green}$	3.1125[5]	3.0612[2]	3.3208[12]	3.3345[5]	3.3731[2]
$M_{red}\cdots M_{red,cis,short}$	3.0100[7]	3.0452[3]	3.2180[17]	3.2375[6]	3.2630[3]
$M_{red}\cdots M_{red,cis,long}$	3.1883[7]	3.1384[3]	3.3959[16]	3.3693[6]	3.4592[4]
$M_{red}\cdots M_{red,trans}$	4.3846[8]	4.3708[3]	4.6783[16]	4.6725[6]	4.7552[4]
S_{blue}–M_{blue}–$S_{blue,chelate}$	88.95[4]	89.70[1]	87.1[1]	87.33[6]	86.74[4]
S_{red}–M_{red}–$S_{green,chelate}$	88.22[3]	88.01[1]	86.2[1]	86.85[4]	86.56[3]
S_{blue}–M_{blue}–$S_{blue.cis}$	90.93[4]	90.09[1]	93.0[1]	92.65[6]	86.74[4]
S_{blue}–M_{red}–$S_{red.cis}$	89.24[2]	90.789[7]	90.37[6]	90.56[3]	89.88[2]
S_{red}–M_{red}–$S_{green,cis}$	93.28[3]	92.84[1]	93.0[1]	92.02[4]	93.57[3]
S_{green}–M_{green}–$S_{green.cis}$	89.99[3]	89.96[1]	89.96[9]	89.91[4]	89.89[3]
S_{blue}–M_{blue}–$S_{blue,trans}$	175.52[5]	174.98[2]	177.9[1]	178.22[6]	178.27[5]
S_{blue}–M_{red}–$S_{green,trans}$	176.89[3]	175.95[2]	177.6[1]	176.92[4]	175.83[3]
S_{red}–M_{red}–$S_{red,trans}$	178.03[4]	177.48[2]	178.7[1]	178.83[4]	177.33[3]
S_{green}–M_{green}–$S_{green,trans}$	178.20[5]	177.02[2]	176.9[1]	175.41[6]	174.80[4]
$\delta_{M,blue}$[e]	0.056	0.074	0.0348	0.0285	0.0113
$\delta_{M,red}$	0.026	0.042	0.0115	0.0002	0.0437
$\delta_{M,green}$	0.015	0.048	0.0516	0.0739	0.0837
θ[f]	43.57(14)	47.10(6)	36.8(6)	37.8(2)	38.5(2)

[a]Chemically equivalent interatomic distances and angles are averaged. For averaged values, uncertainties are determined according to the general formula for error propagation, as described by Taylor,[21] and are enclosed in square brackets. [b]The subscripted colors following atoms M, S and C in the leftmost column designate specific interatomic distances, displacements or angles as defined in **Figure 3** (**a**). [c]Values are for $[Pd_6(S_2C_2Ph_2)_6]\cdot PhNO_2$. [d]Values are for $[Pd_6(S_2C_2Ph_2)_6]\cdot 2½CH_2Cl_2$. [e]δ = Absolute value of displacement (Å) of M from S_4M mean plane. [f]θ = angle between the S_2C_2 mean planes in blue fragment, **Figure 3** (**a**).

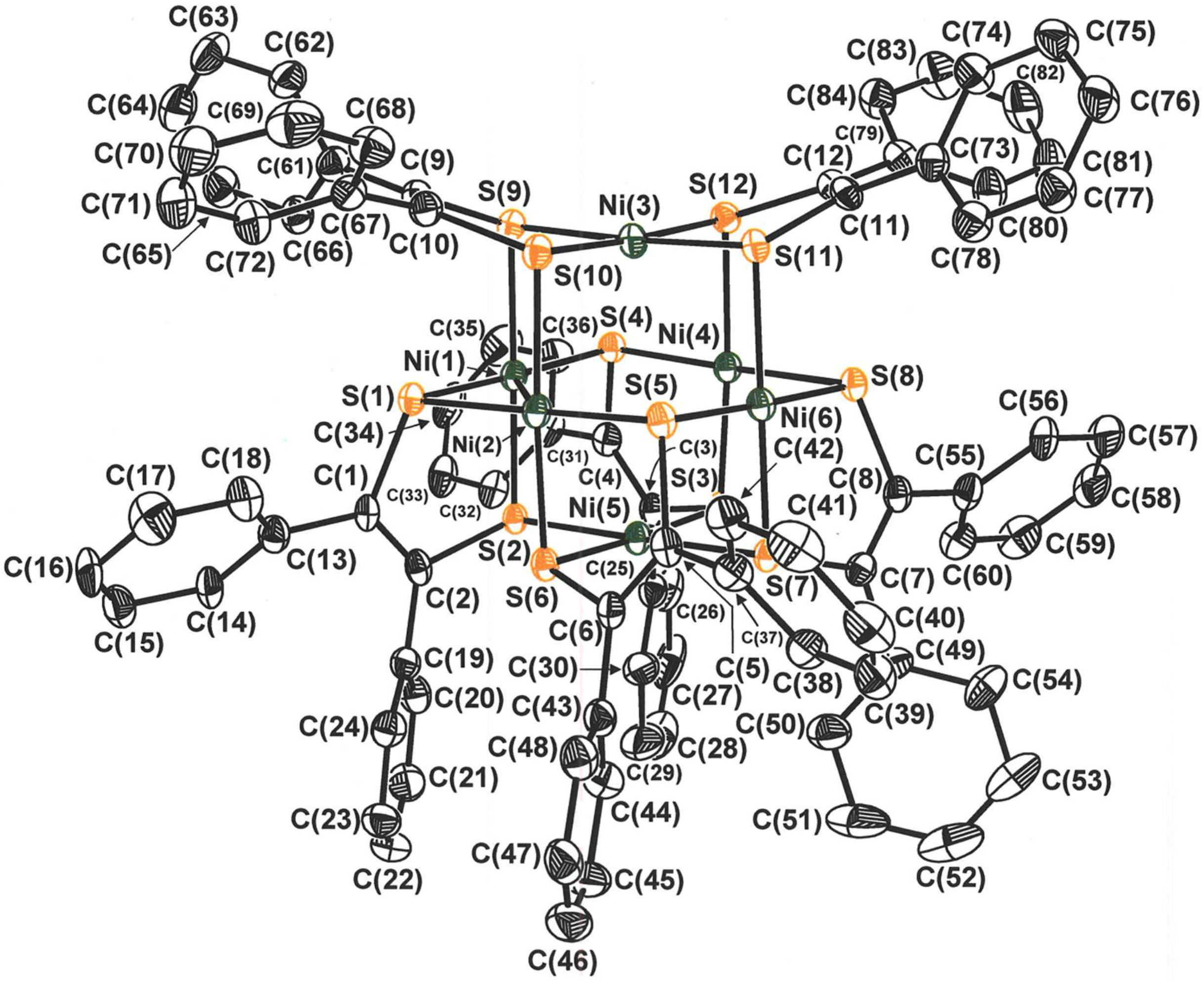

Figure S1. Thermal ellipsoid plot of $[Ni_6(S_2C_2Ph_2)_6]$ with complete atom labeling. Ellipsoids are drawn at the 50% probability level, and all H atoms are omitted for clarity.

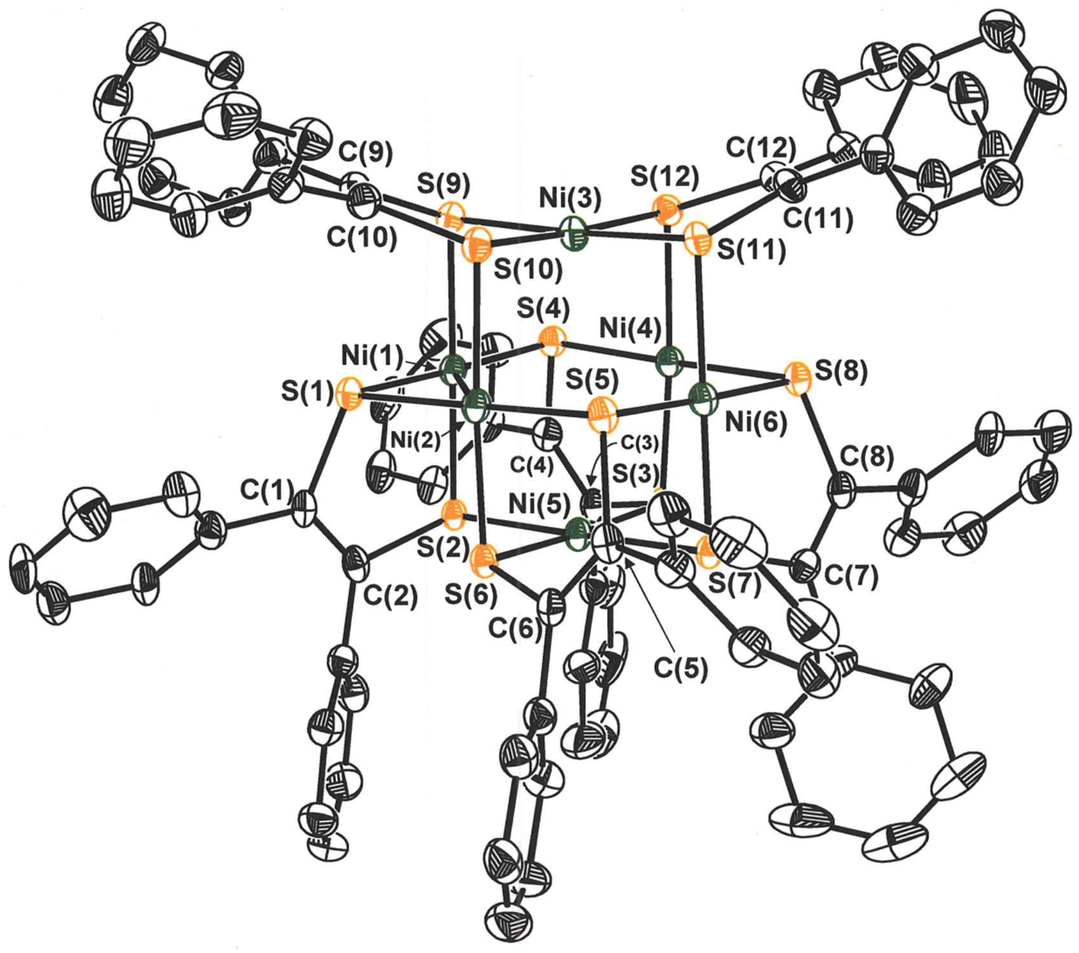

Figure S2. Thermal ellipsoid plot of $[Ni_6(S_2C_2Ph_2)_6]$ with partial atom labeling. Ellipsoids are drawn at the 50% probability level, and all H atoms are omitted for clarity.

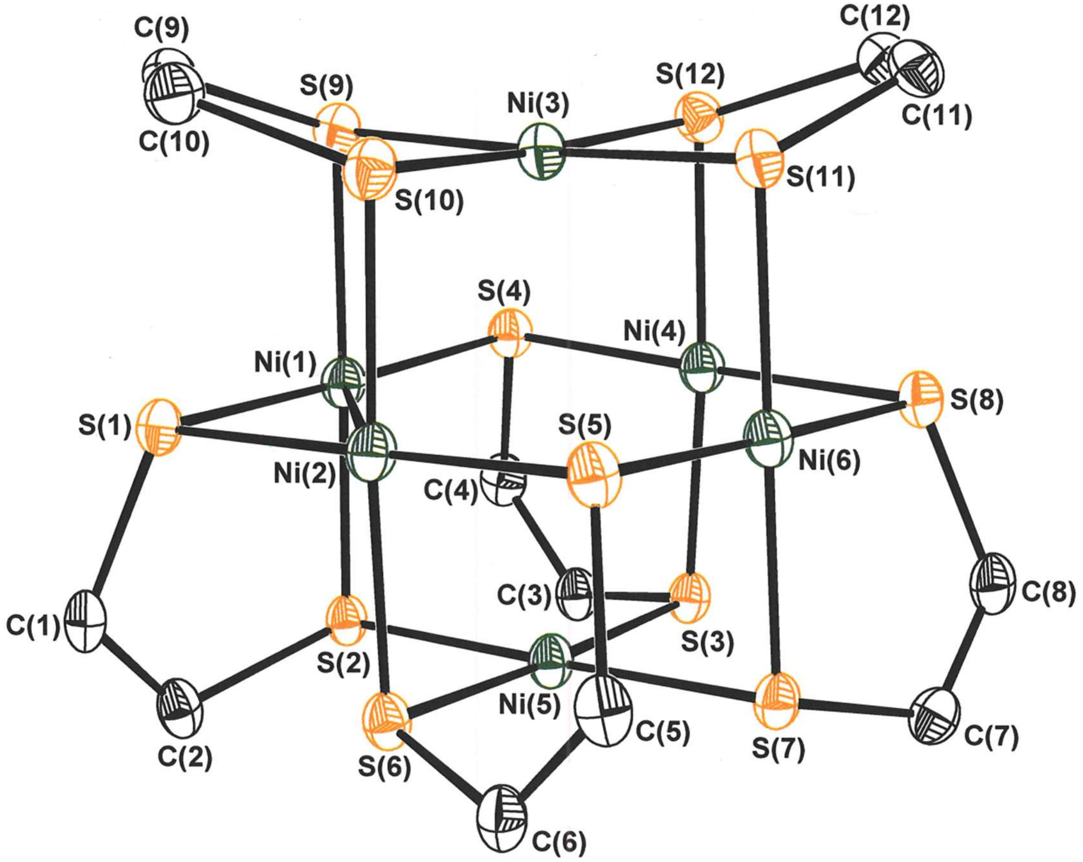

Figure S3. Thermal ellipsoid plot of the core structure of $[Ni_6(S_2C_2Ph_2)_6]$ with atom labeling. Ellipsoids are drawn at the 50% probability level. The structure is best described as a D_{2h}-symmetric square planar $Ni(S_2C_2Ph_2)_2$ group (Ni3) affixed to a C_4-symmetric fragment in which C_2S_2 groups form the blades of a 4-bladed propeller that has Ni5 as its nexus.

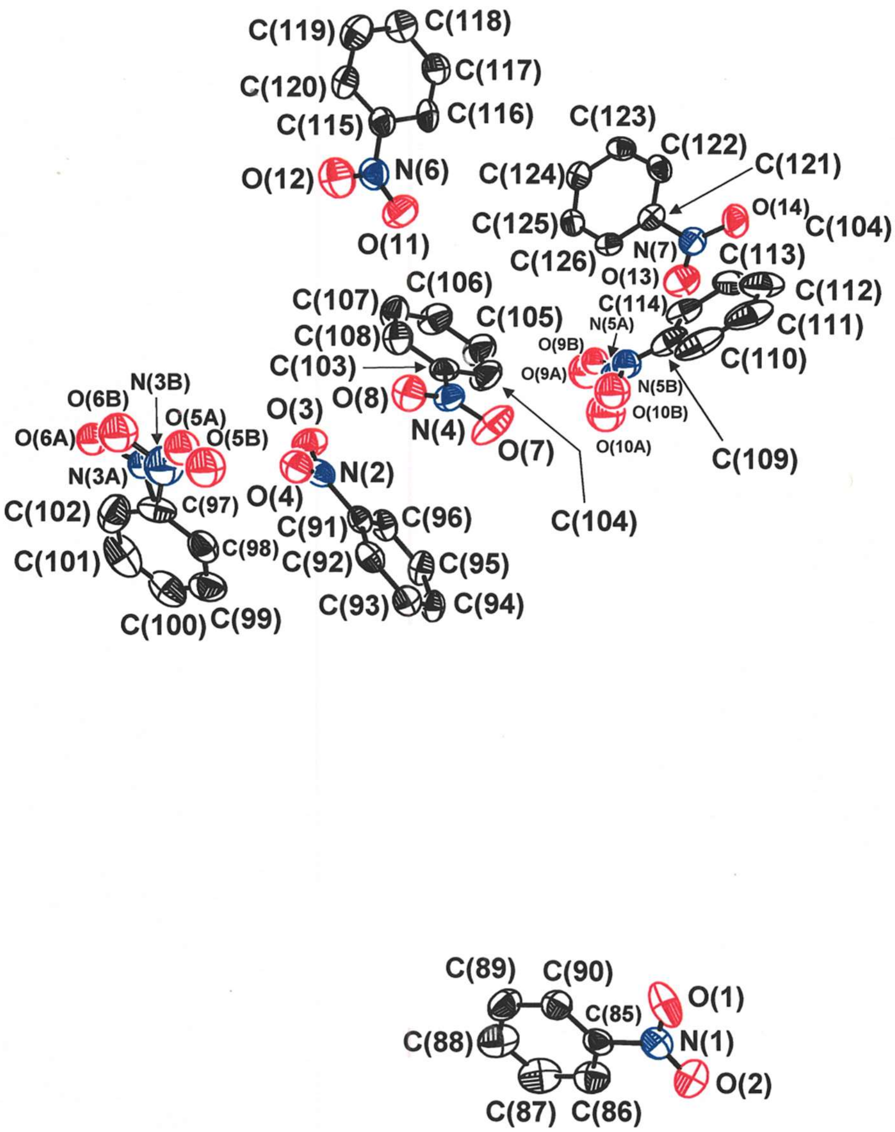

Figure S4. Thermal ellipsoid plot (50%) of interstitial solvent in $[Ni_6(S_2C_2Ph_2)_6]\cdot 7(C_6H_5NO_2)$ with complete atom labeling. All H atoms are omitted for clarity.

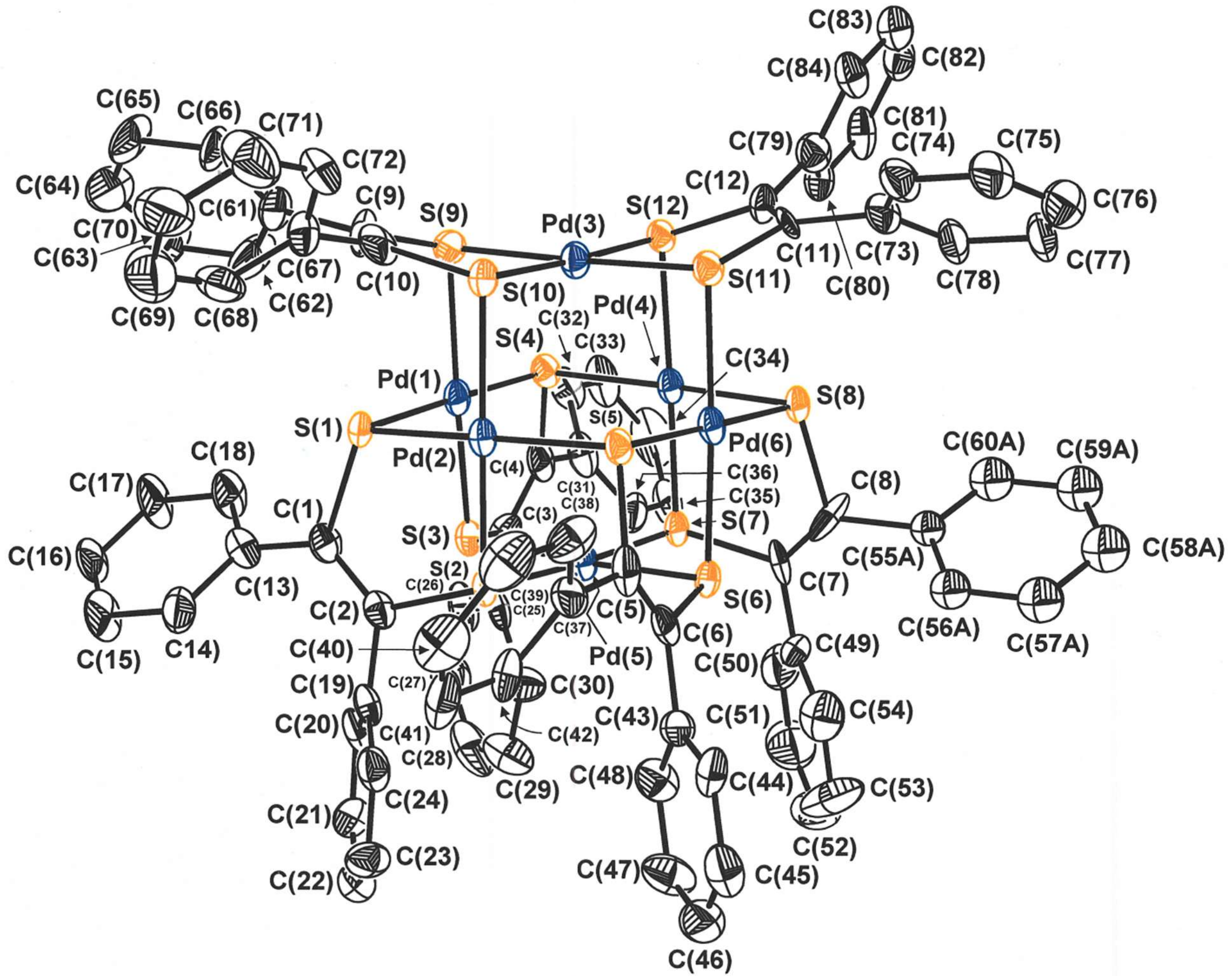

Figure S5. Thermal ellipsoid plot of $[Pd_6(S_2C_2Ph_2)_6]$ in $[Pd_6(S_2C_2Ph_2)_6]\cdot PhNO_2$ with complete atom labeling. Ellipsoids are drawn at the 50% probability level, and all H atoms are omitted for clarity.

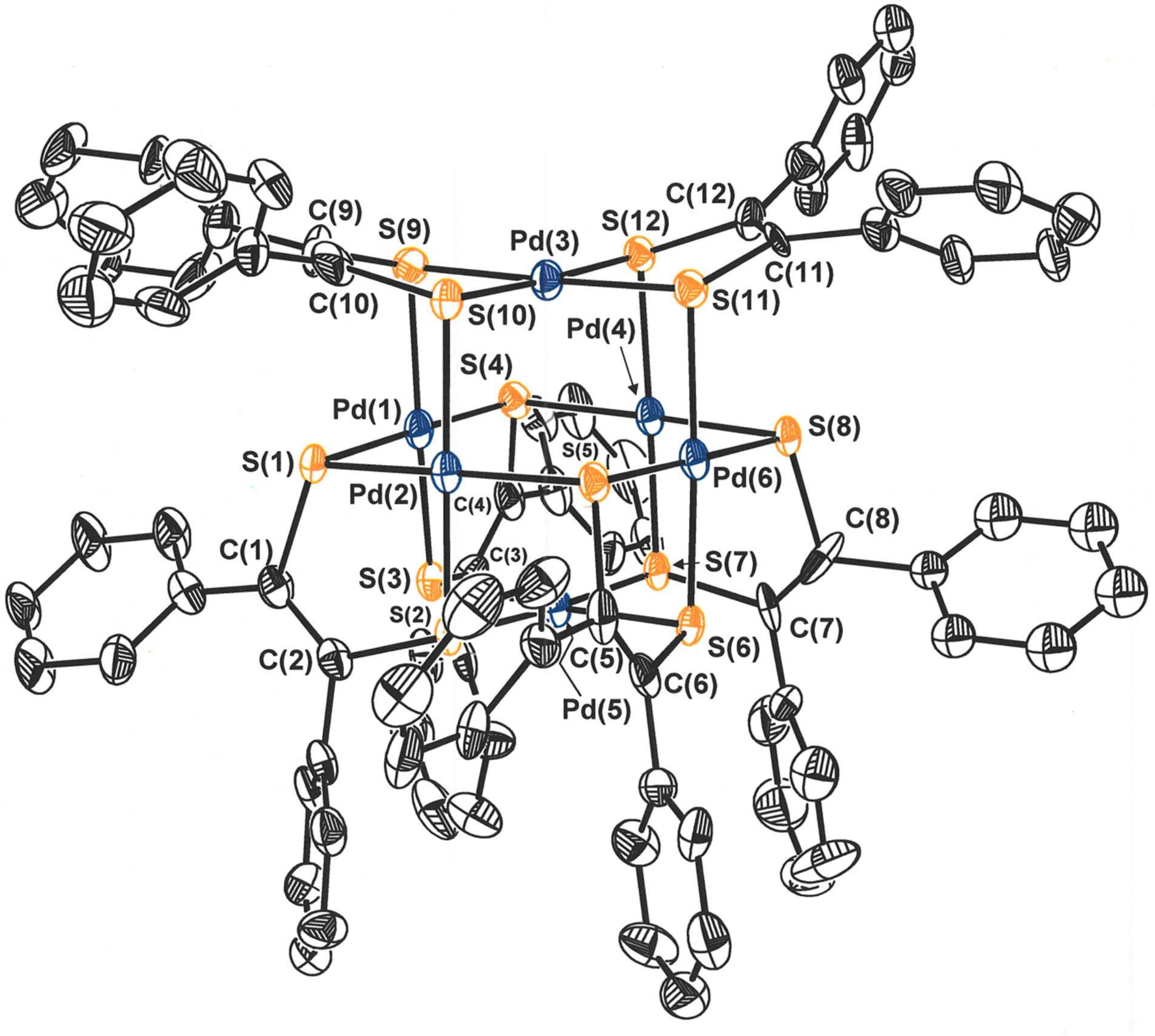

Figure S6. Thermal ellipsoid plot of $[Pd_6(S_2C_2Ph_2)_6]$ in $[Pd_6(S_2C_2Ph_2)_6]\cdot PhNO_2$ with partial atom labeling. Ellipsoids are drawn at the 50% probability level, and all H atoms are omitted for clarity.

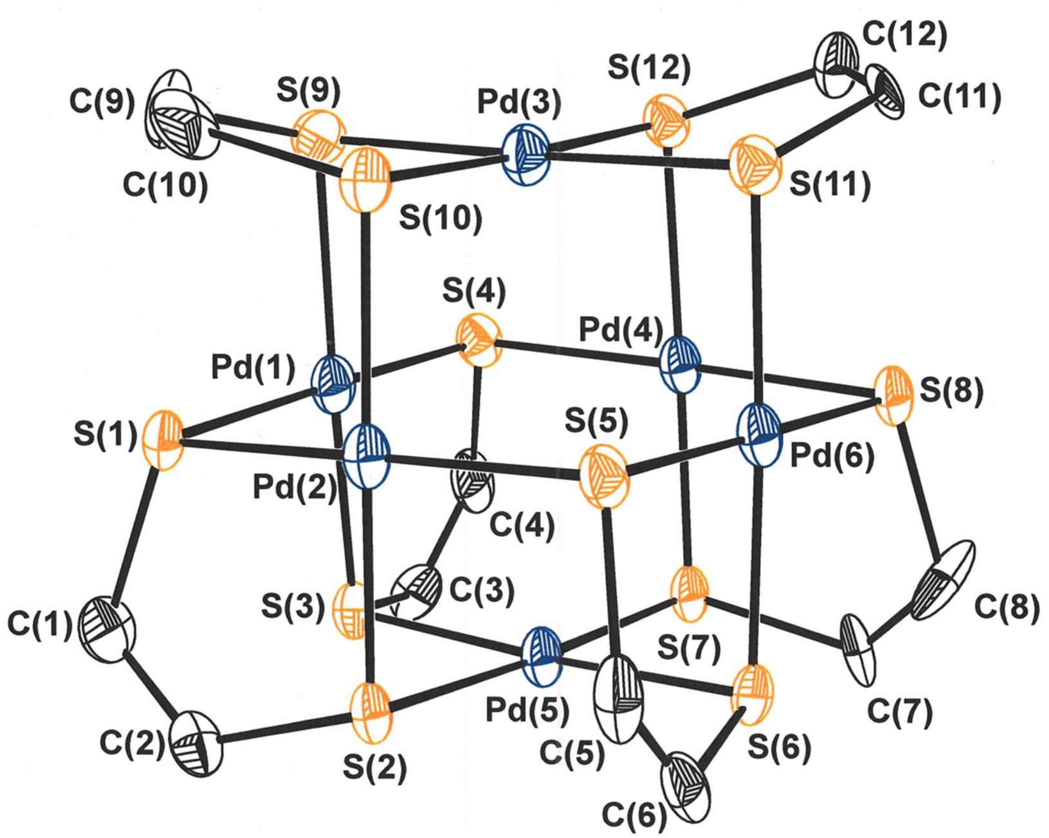

Figure S7. Thermal ellipsoid plot of the core structure of $[Pd_6(S_2C_2Ph_2)_6]$ in $[Pd_6(S_2C_2Ph_2)_6]\cdot PhNO_2$ with atom labeling. Ellipsoids are drawn at the 50% probability level. The structure is best described as a D_{2h}-symmetric square planar $Pd(S_2C_2Ph_2)_2$ group (Pd3) affixed to a C_4-symmetric fragment in which C_2S_2 groups form the blades of a 4-bladed propeller that has Pd5 as its nexus.

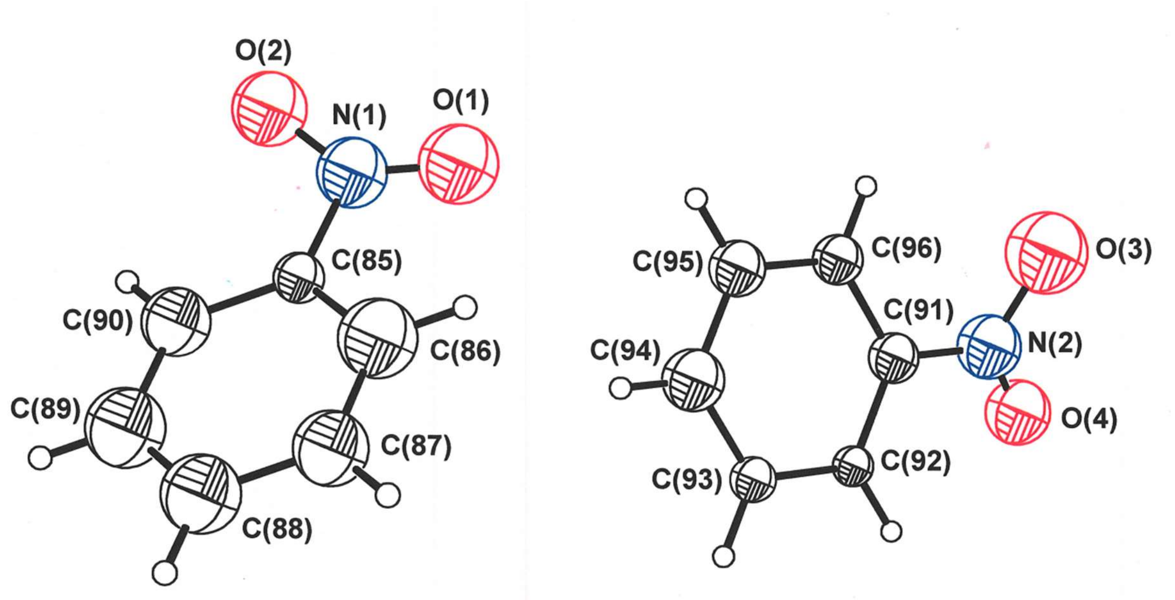

Figure S8. Thermal ellipsoid plot (30%) of interstitial solvent in $[Pd_6(S_2C_2Ph_2)_6]\cdot(C_6H_5NO_2)$ with complete atom labeling.

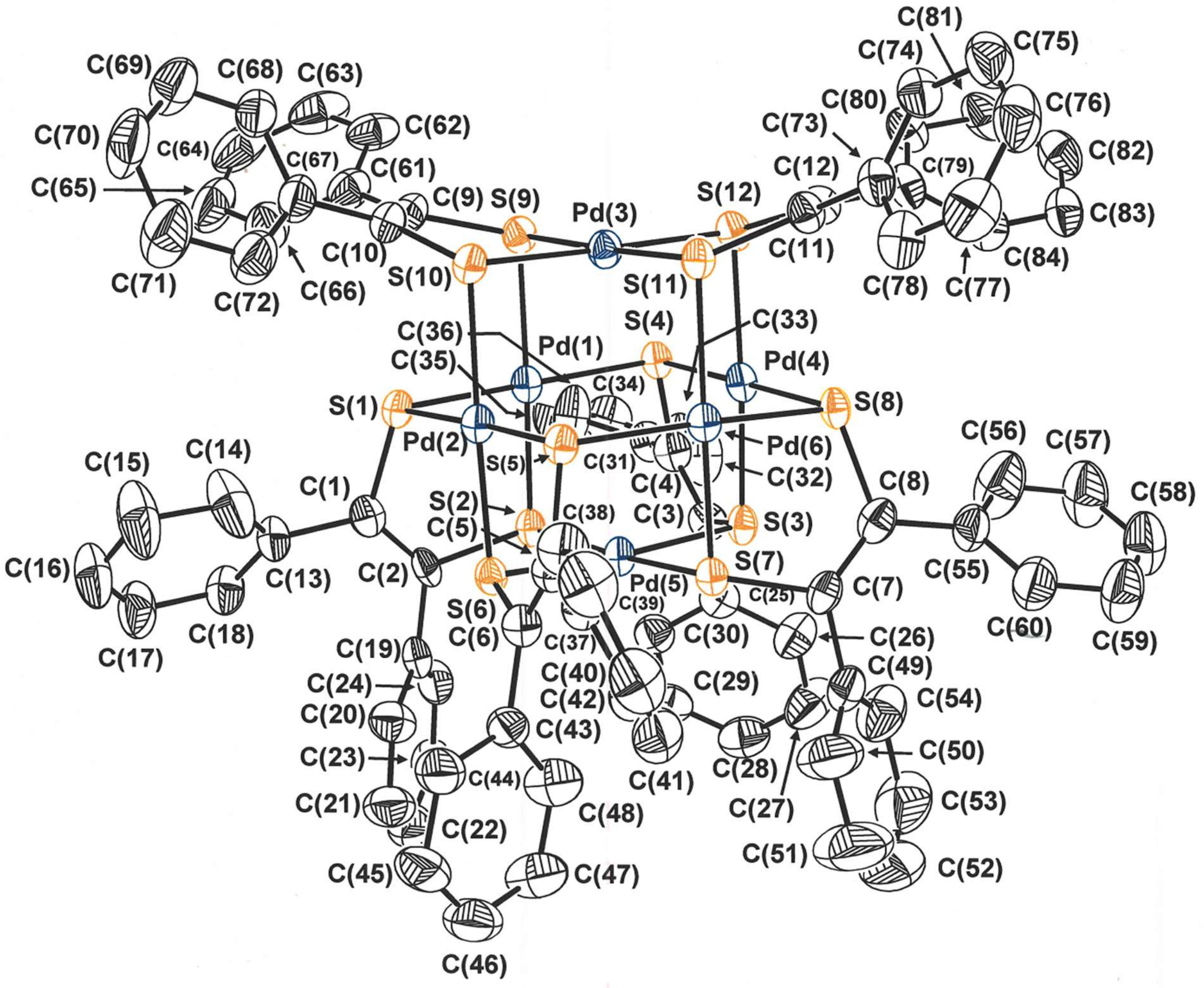

Figure S9. Thermal ellipsoid plot of $[Pd_6(S_2C_2Ph_2)_6]$ in $[Pd_6(S_2C_2Ph_2)_6]\cdot 2½CH_2Cl_2$ with complete atom labeling. Ellipsoids are drawn at the 50% probability level, and all H atoms are omitted for clarity.

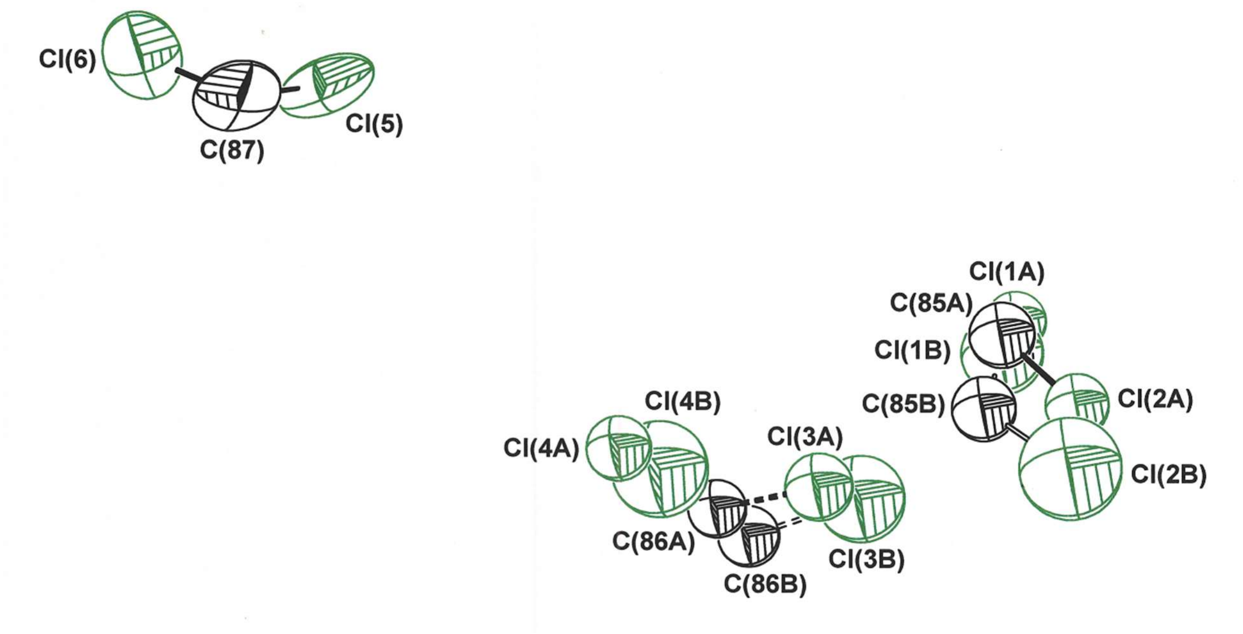

Figure S10. Thermal ellipsoid plot of interstitial CH_2Cl_2 in $[Pd_6(S_2C_2Ph_2)_6]\cdot 2\frac{1}{2}CH_2Cl_2$ with atom labeling. Ellipsoids are drawn at the 50% probability level, and all H atoms are omitted for clarity.

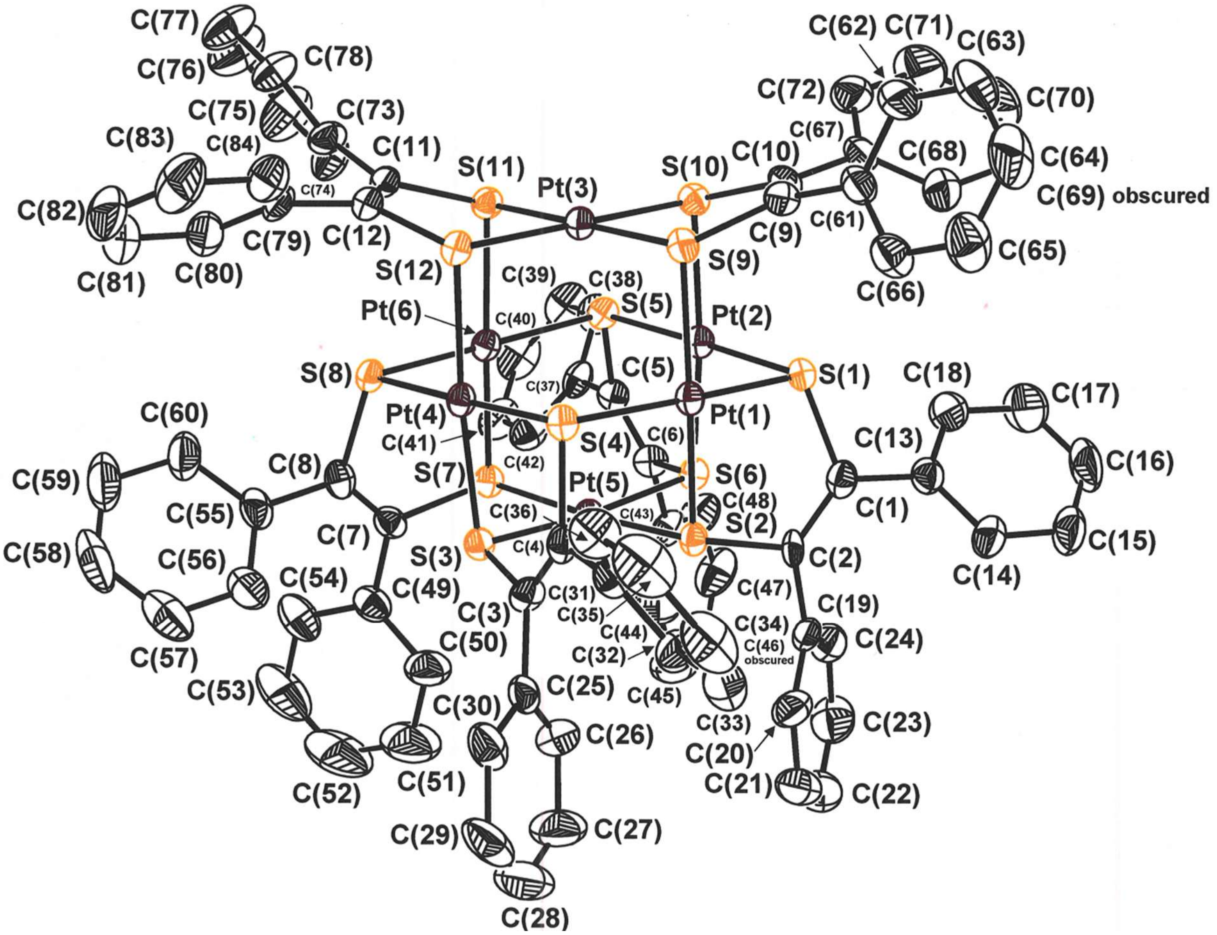

Figure S11. Thermal ellipsoid plot of $[Pt_6(S_2C_2Ph_2)_6]$ with complete atom labeling. Ellipsoids are drawn at the 50% probability level, and all H atoms are omitted for clarity.

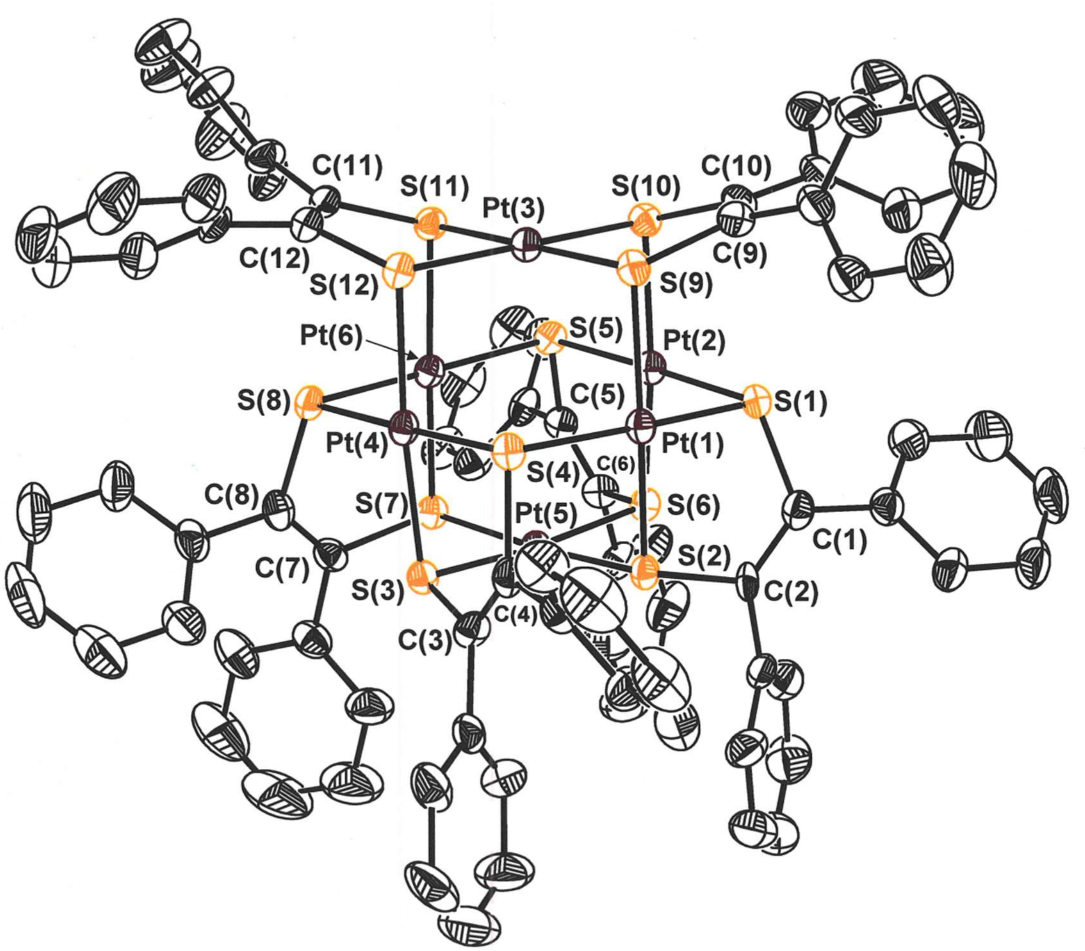

Figure S12. Thermal ellipsoid plot of $[Pt_6(S_2C_2Ph_2)_6]$ with partial atom labeling. Ellipsoids are drawn at the 50% probability level, and all H atoms are omitted for clarity.

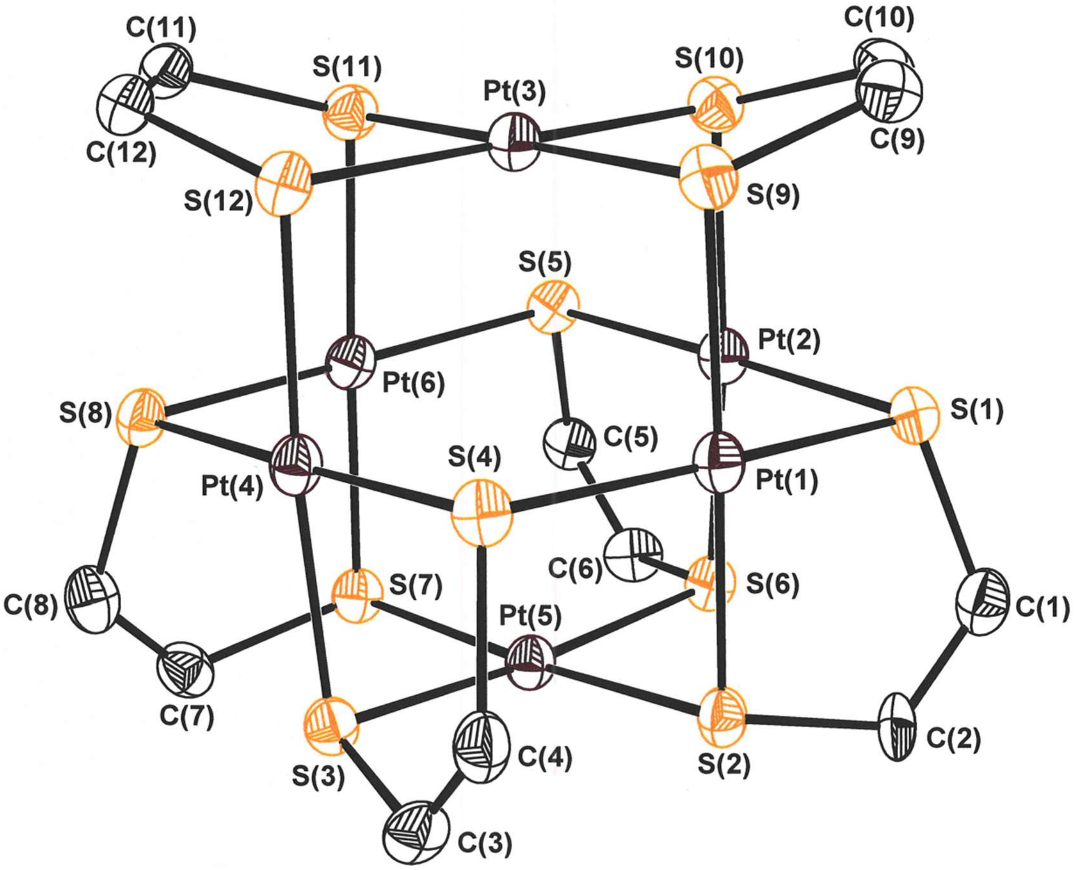

Figure S13. Thermal ellipsoid plot of the core structure of $[Pt_6(S_2C_2Ph_2)_6]$ with atom labeling. Ellipsoids are drawn at the 50% probability level. The structure is best described as a D_{2h}-symmetric square planar $Pt(S_2C_2Ph_2)_2$ group (Pt3) affixed to a C_4-symmetric fragment in which C_2S_2 groups form the blades of a 4-bladed propeller that has Pt5 as its nexus.

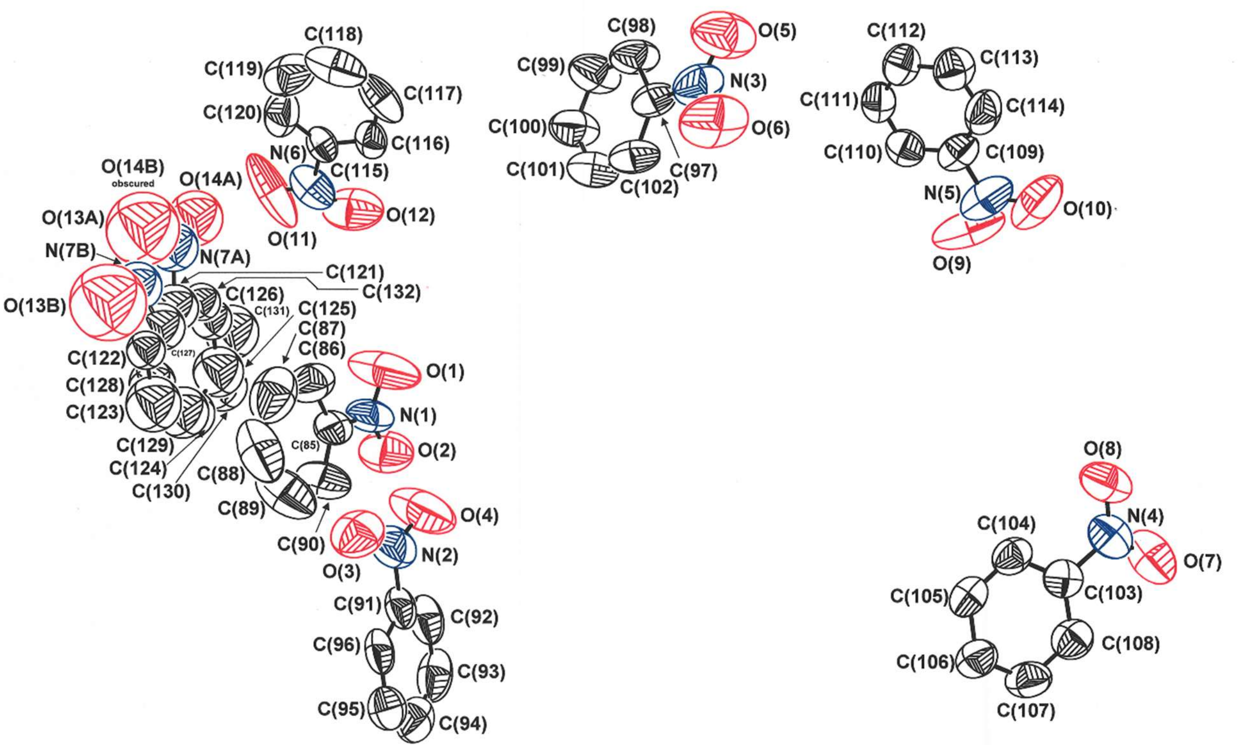

Figure S14. Thermal ellipsoid plot (50%) of interstitial solvent in $[Pt_6(S_2C_2Ph_2)_6]\cdot7(C_6H_5NO_2)$ with complete atom labeling. All H atoms are omitted for clarity.

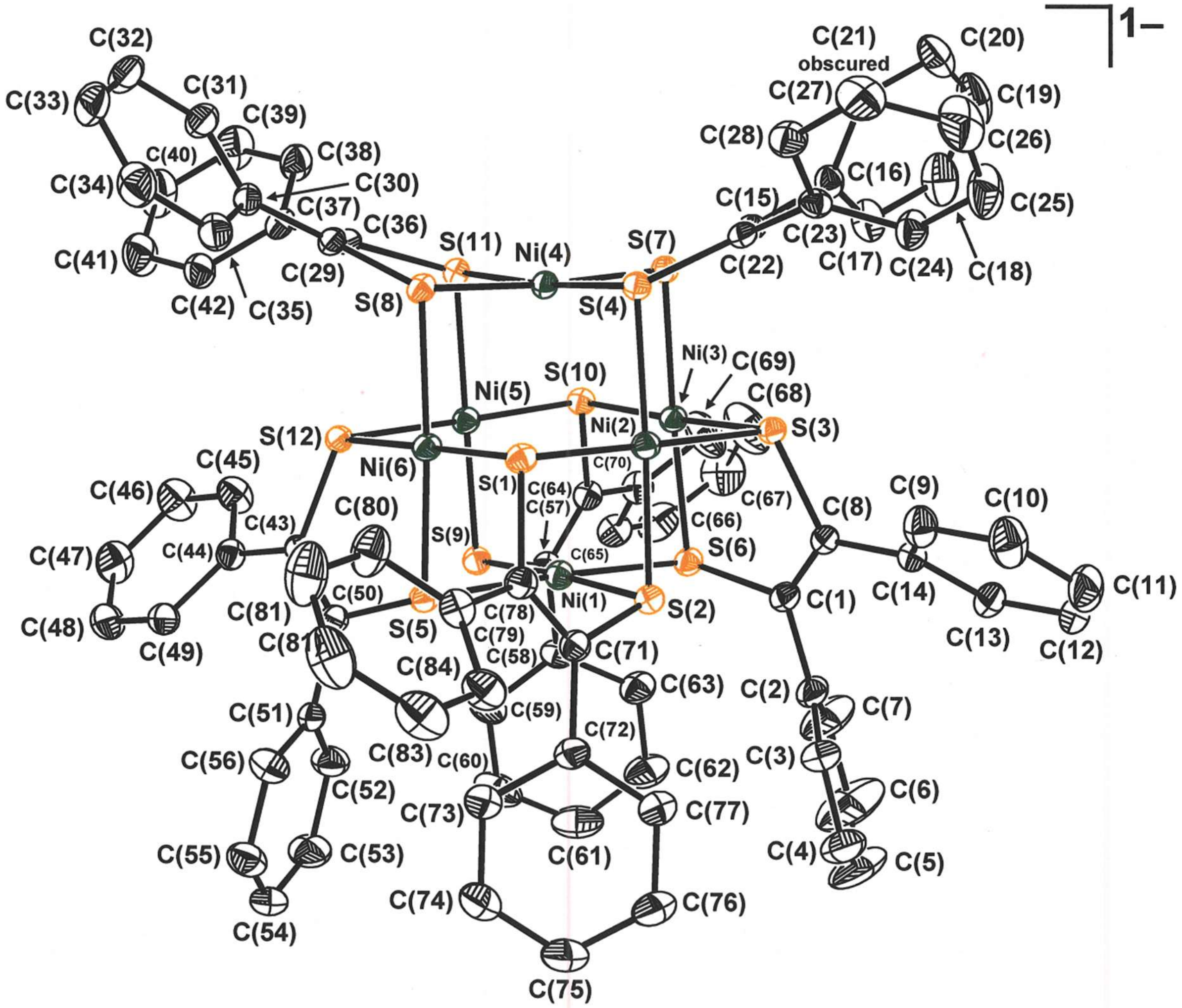

Figure S15. Thermal ellipsoid plot of $[Ni_6(S_2C_2Ph_2)_6]^{1-}$ with complete atom labeling. Ellipsoids are drawn at the 50% probability level, and all H atoms are omitted for clarity.

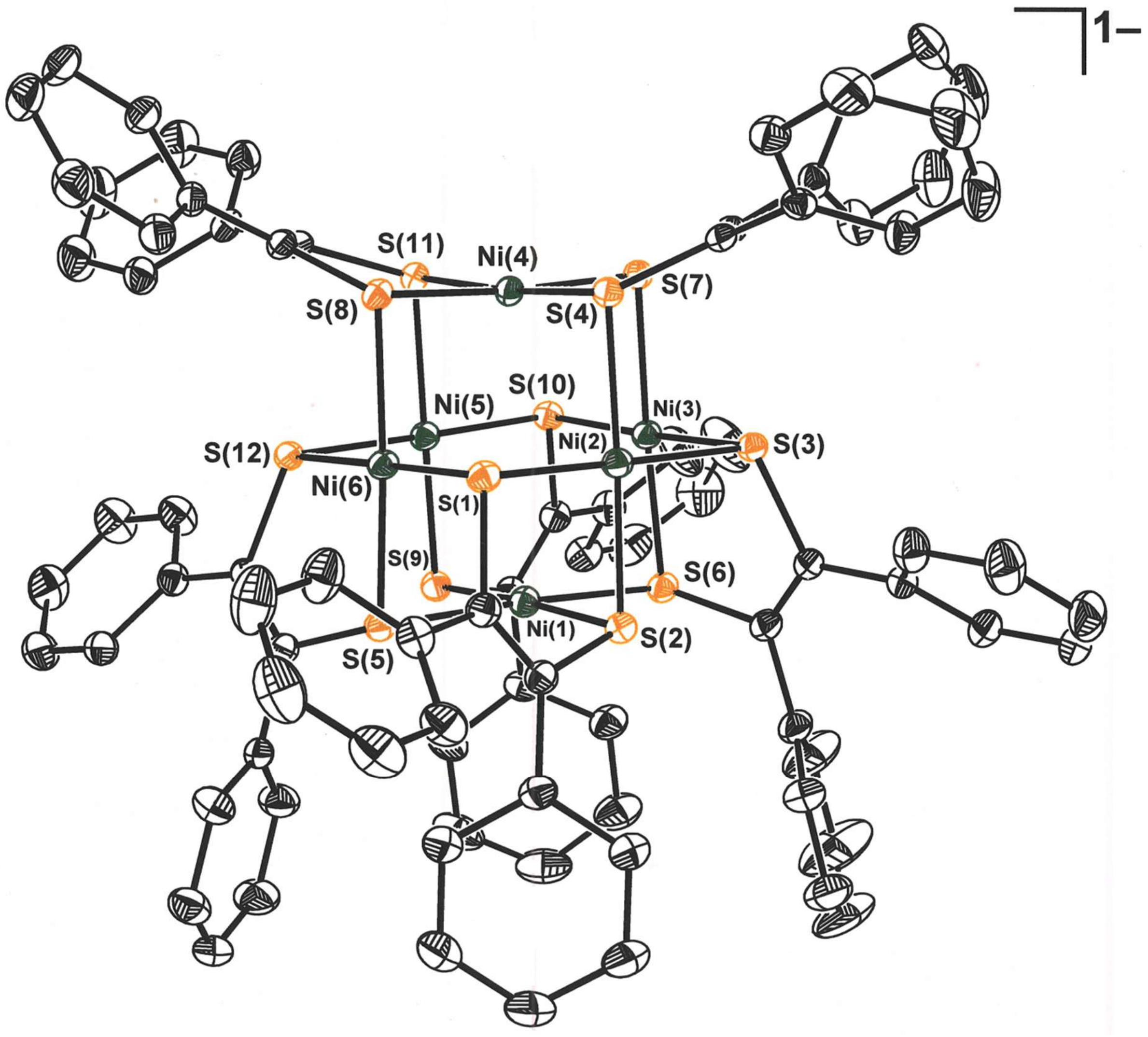

Figure S16. Thermal ellipsoid plot of $[Ni_6(S_2C_2Ph_2)_6]^{1-}$ with partial atom labeling. Ellipsoids are drawn at the 50% probability level, and all H atoms are omitted for clarity.

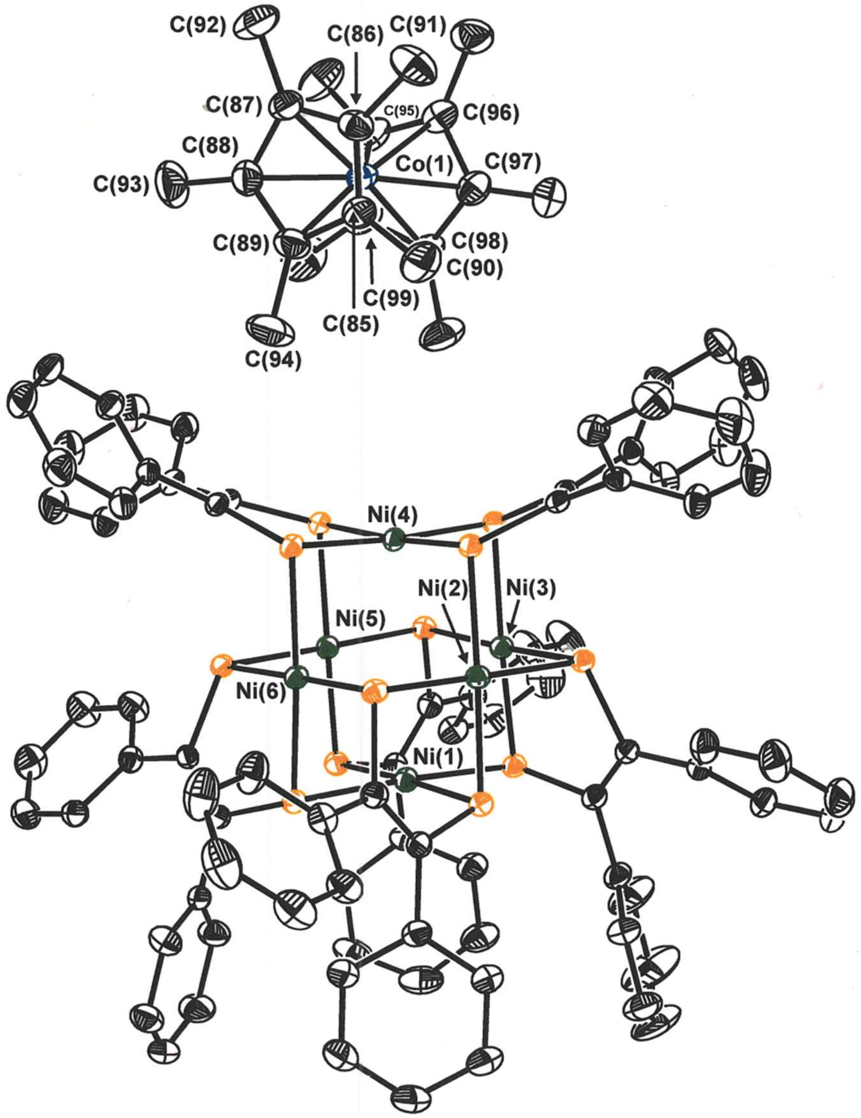

Figure S17. Thermal ellipsoid plot of $[Cp^*_2Co][Ni_6(S_2C_2Ph_2)_6]$ with atom labeling shown for the cation. Ellipsoids are drawn at the 50% probability level, and all H atoms are omitted for clarity.

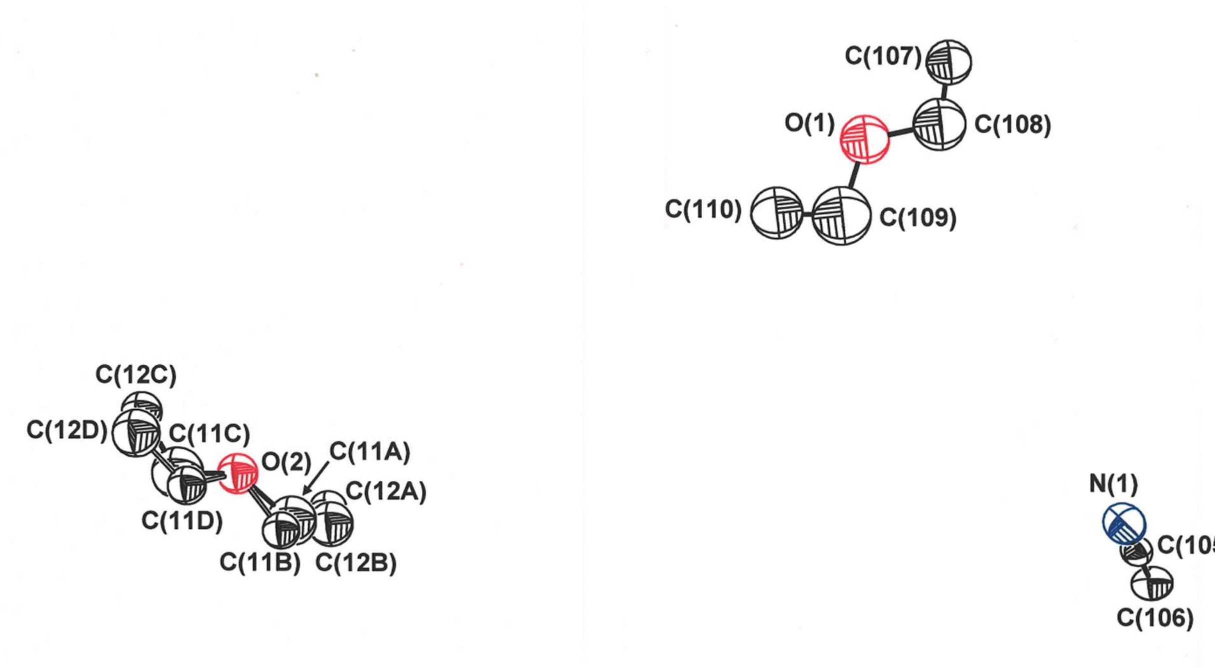

Figure S18. Thermal ellipsoid plot with atom labeling for interstitial solvent in $[Cp^*{}_2Co][Ni_6(S_2C_2Ph_2)_6]\cdot MeCN\cdot 0.75(Et_2O)$. Ellipsoids are drawn at the 50% probability level, and all H atoms are omitted for clarity.

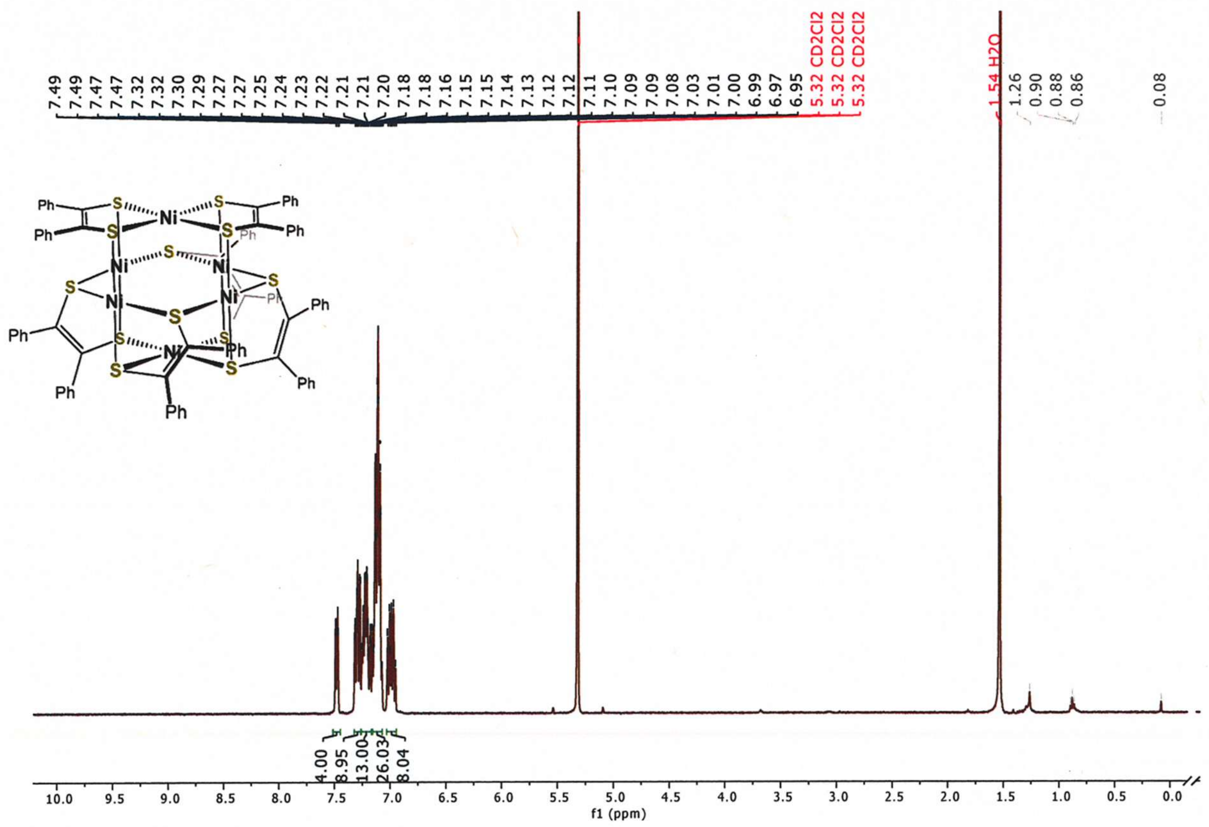

Figure S19. ^{1}H NMR spectrum of $[Ni_6(S_2C_2Ph_2)_6]$ in CD_2Cl_2, 0.0 - 10.0 ppm.

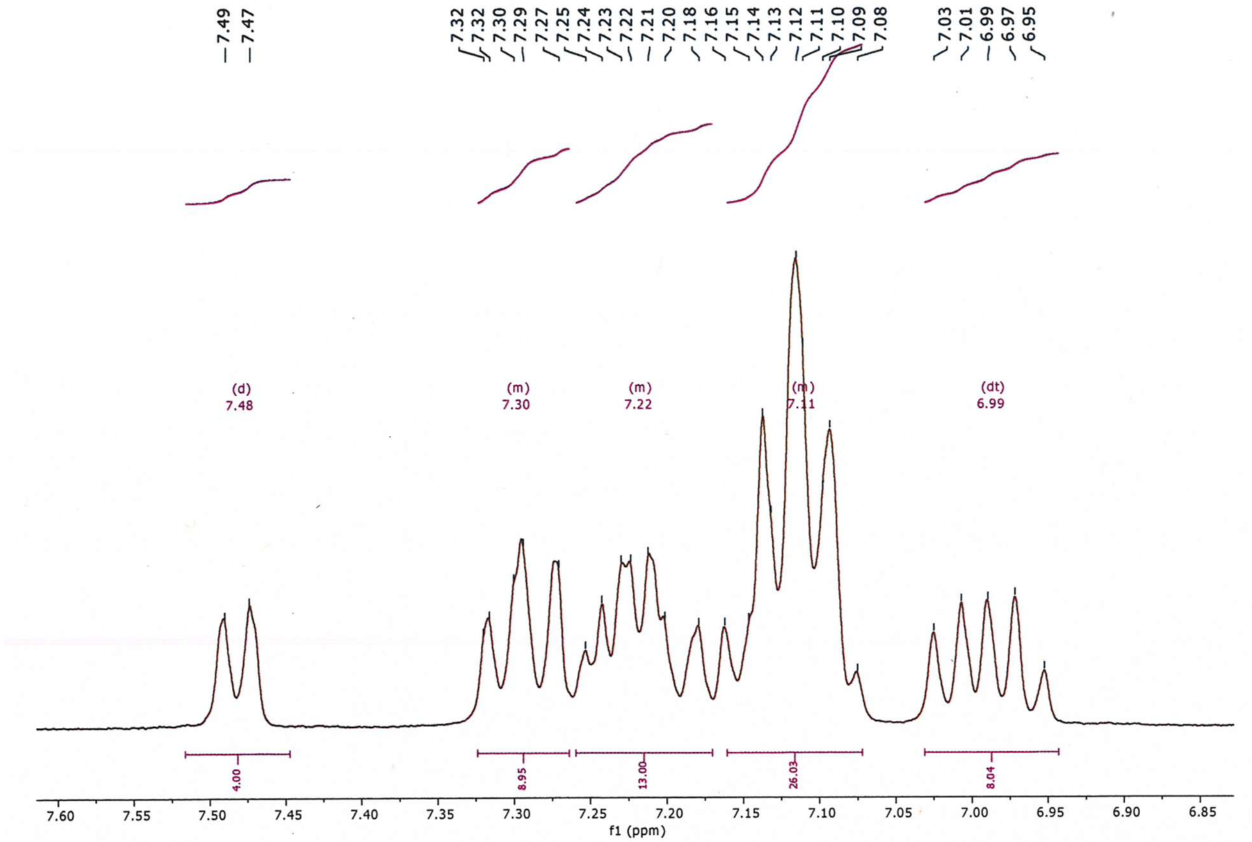

Figure S20. ^{1}H NMR spectrum of $[Ni_6(S_2C_2Ph_2)_6]$ in CD_2Cl_2, 6.85 - 7.60 ppm.

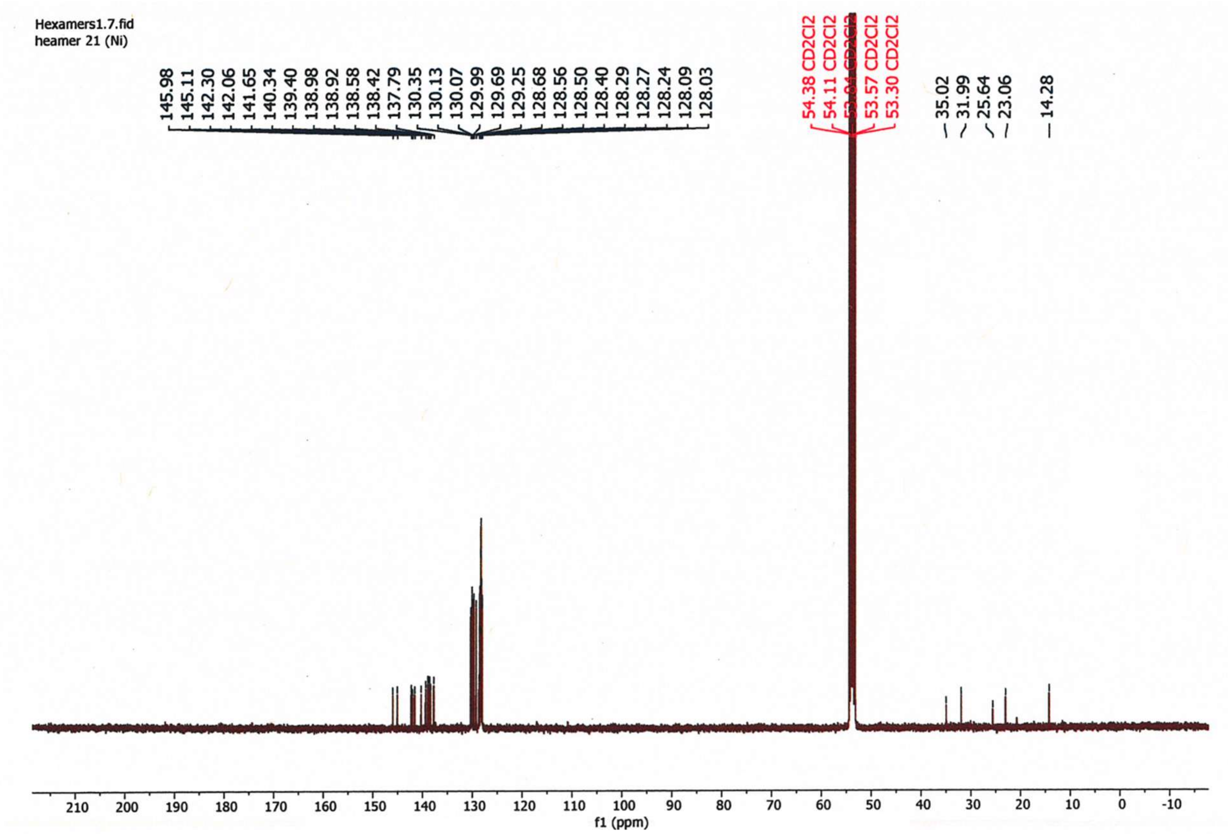

Figure S21. ^{13}C NMR spectrum of [$Ni_6(S_2C_2Ph_2)_6$] in CD_2Cl_2, –10.0 - 215.0 ppm.

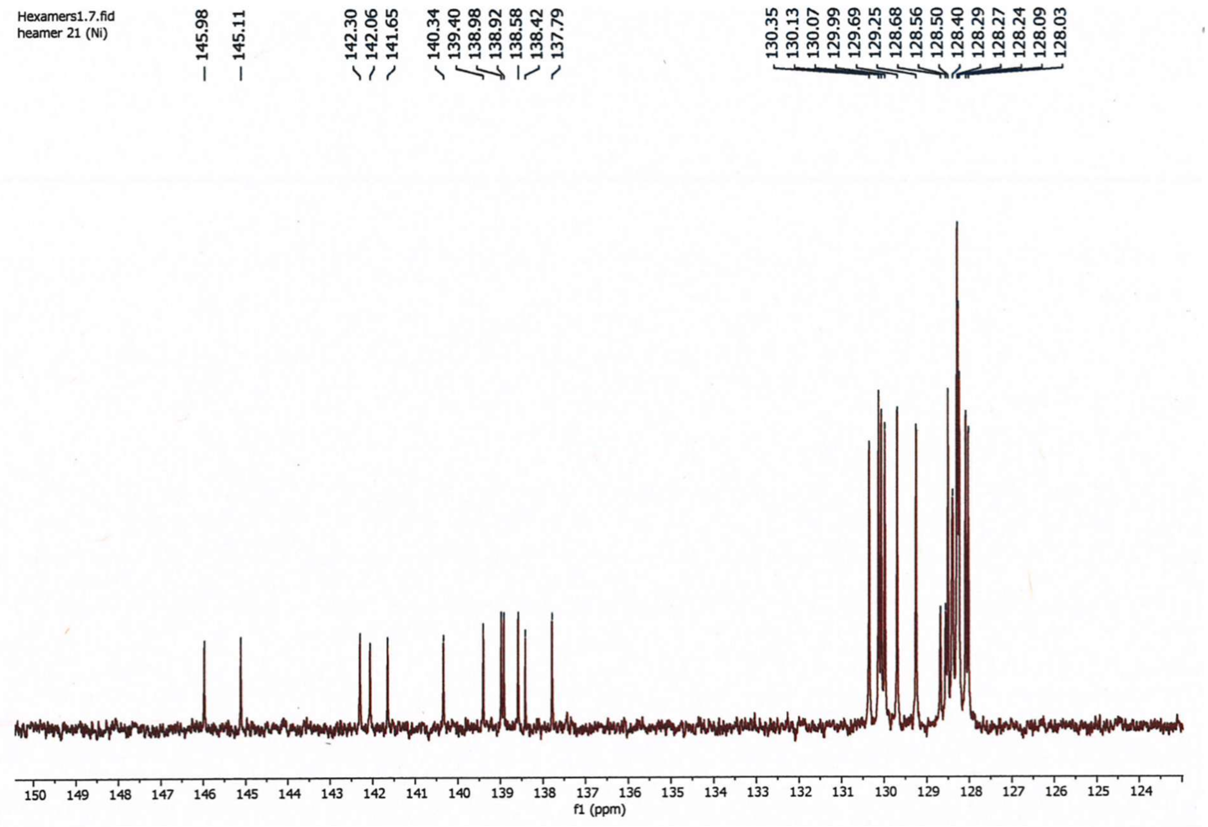

Figure S22. ^{13}C NMR spectrum of $[Ni_6(S_2C_2Ph_2)_6]$ in CD_2Cl_2, 123 - 150 ppm.

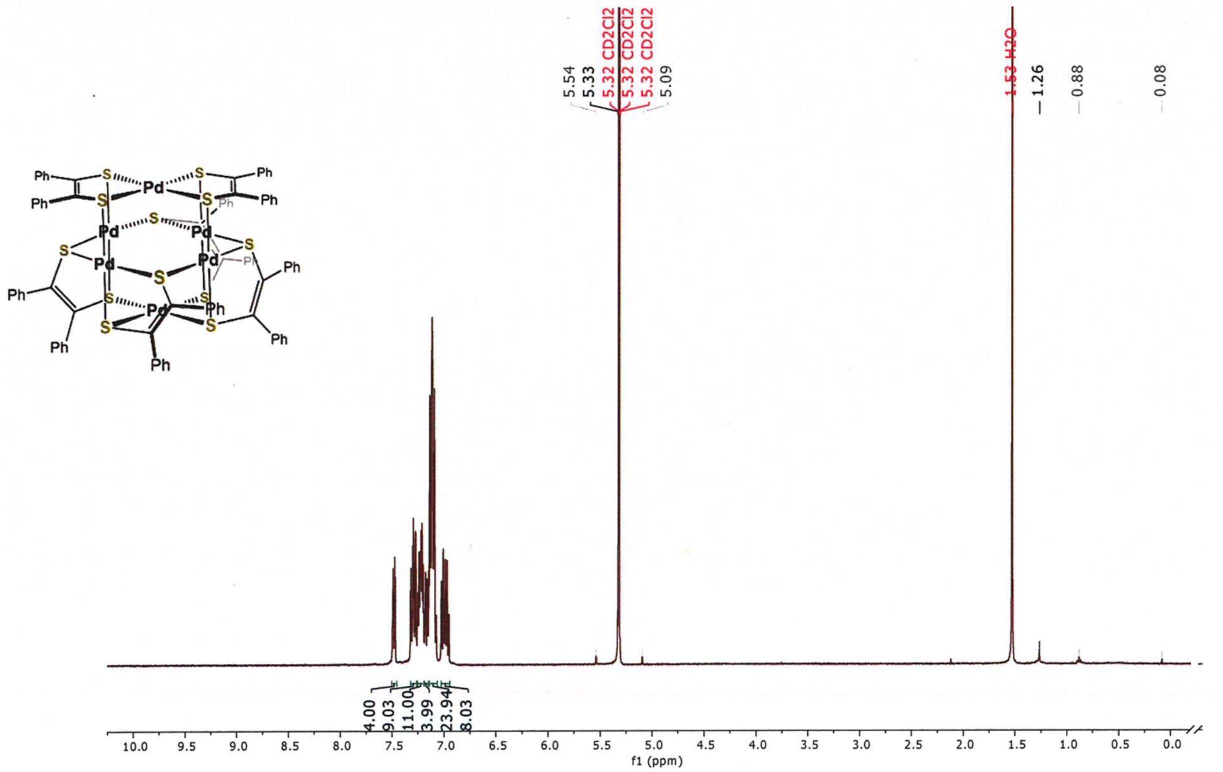

Figure S23. ^{1}H NMR spectrum of $[Pd_6(S_2C_2Ph_2)_6]$ in CD_2Cl_2, 0.0 - 10.0 ppm.

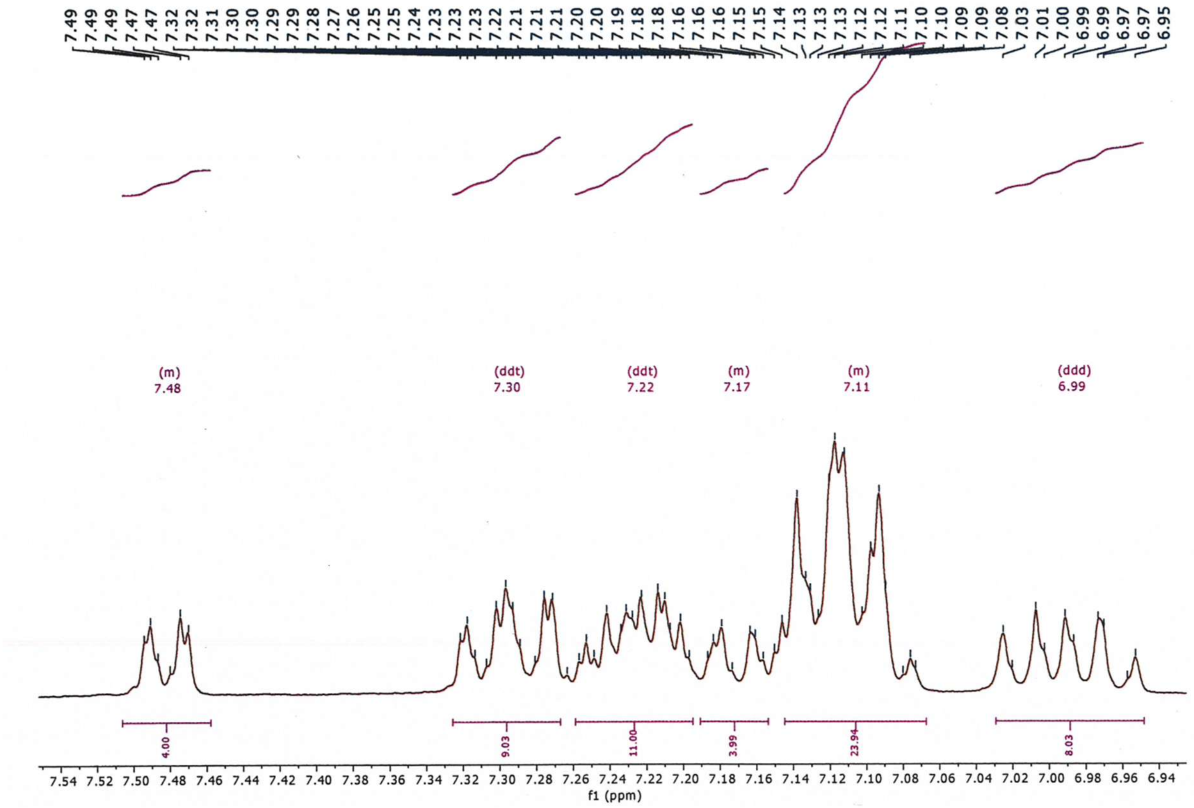

Figure S24. ^{1}H NMR spectrum of $[Pd_6(S_2C_2Ph_2)_6]$ in CD_2Cl_2, 6.93 - 7.55 ppm.

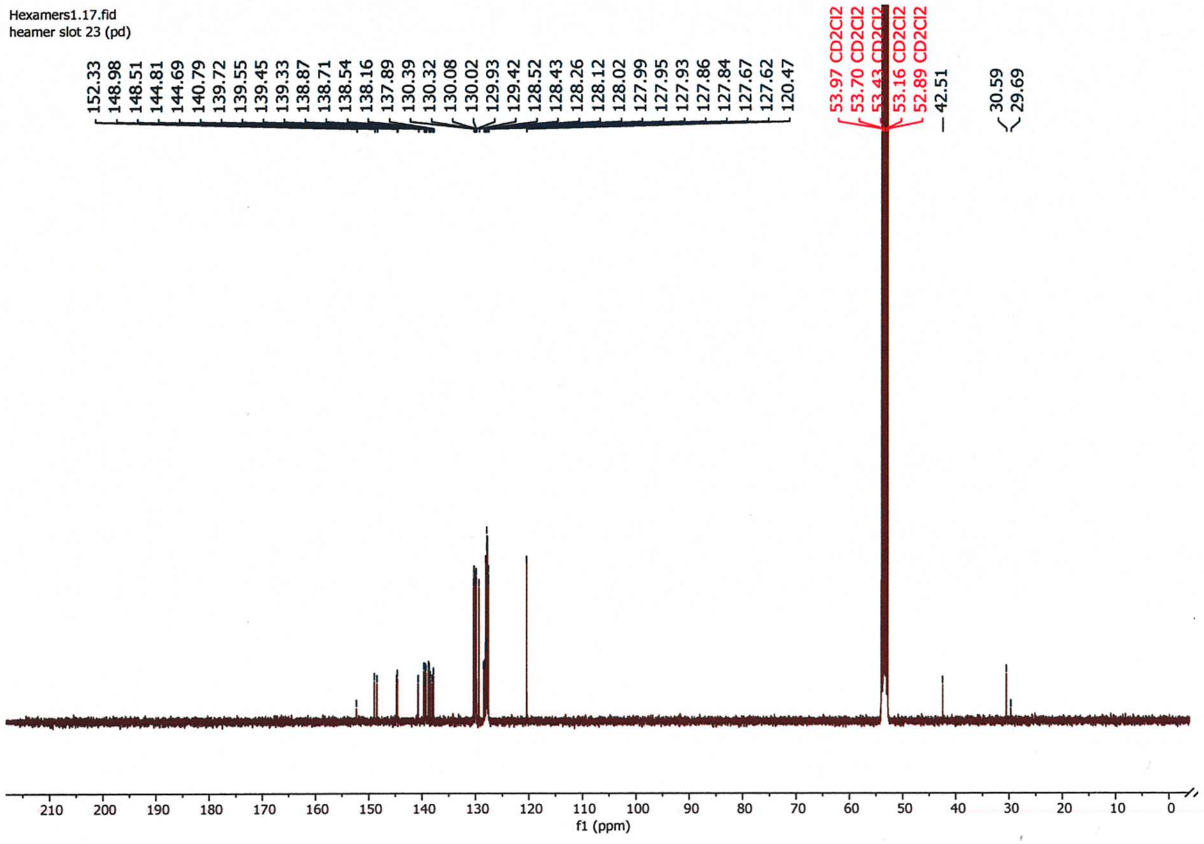

Figure S25. ^{13}C NMR spectrum of $[Pd_6(S_2C_2Ph_2)_6]$ in CD_2Cl_2, 0.0 - 215.0 ppm.

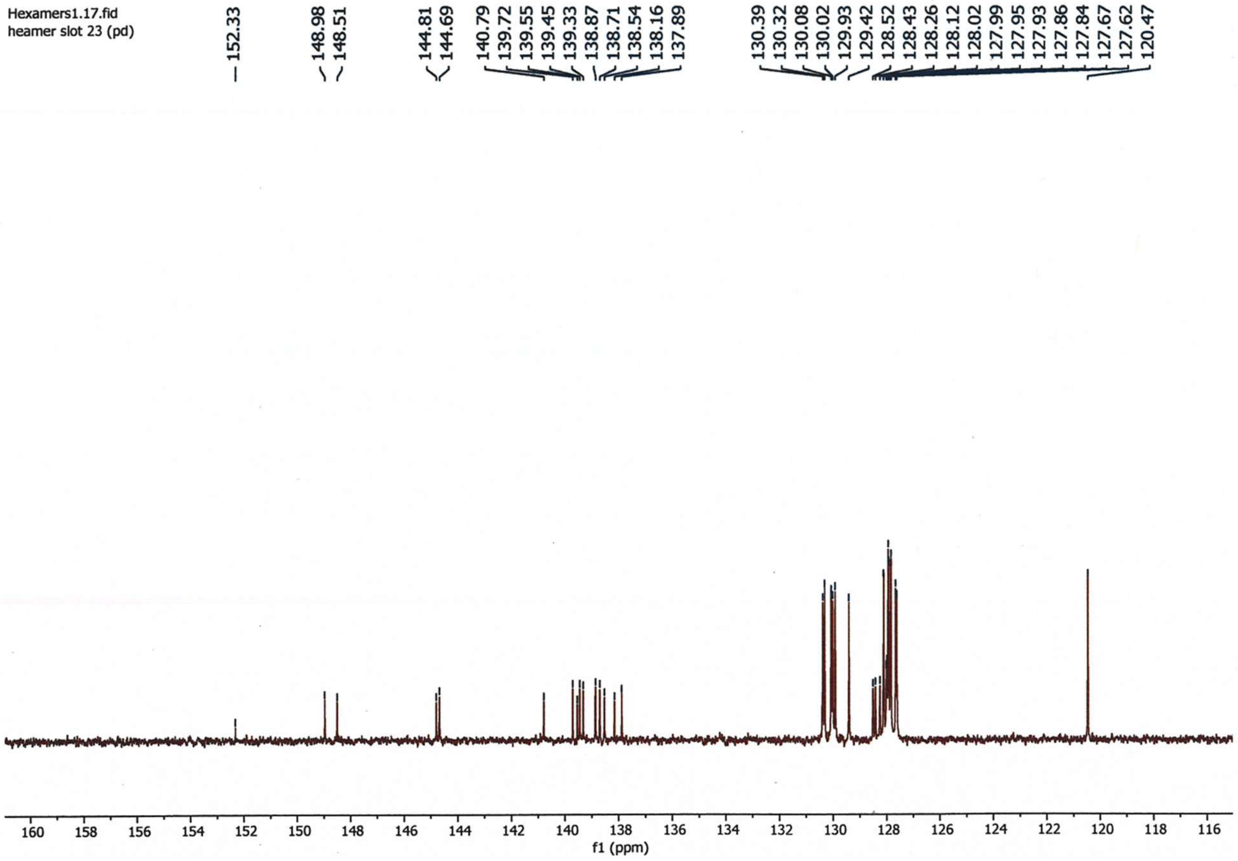

Figure S26. ^{13}C NMR spectrum of $[Pd_6(S_2C_2Ph_2)_6]$ in CD_2Cl_2, 115 - 160 ppm.

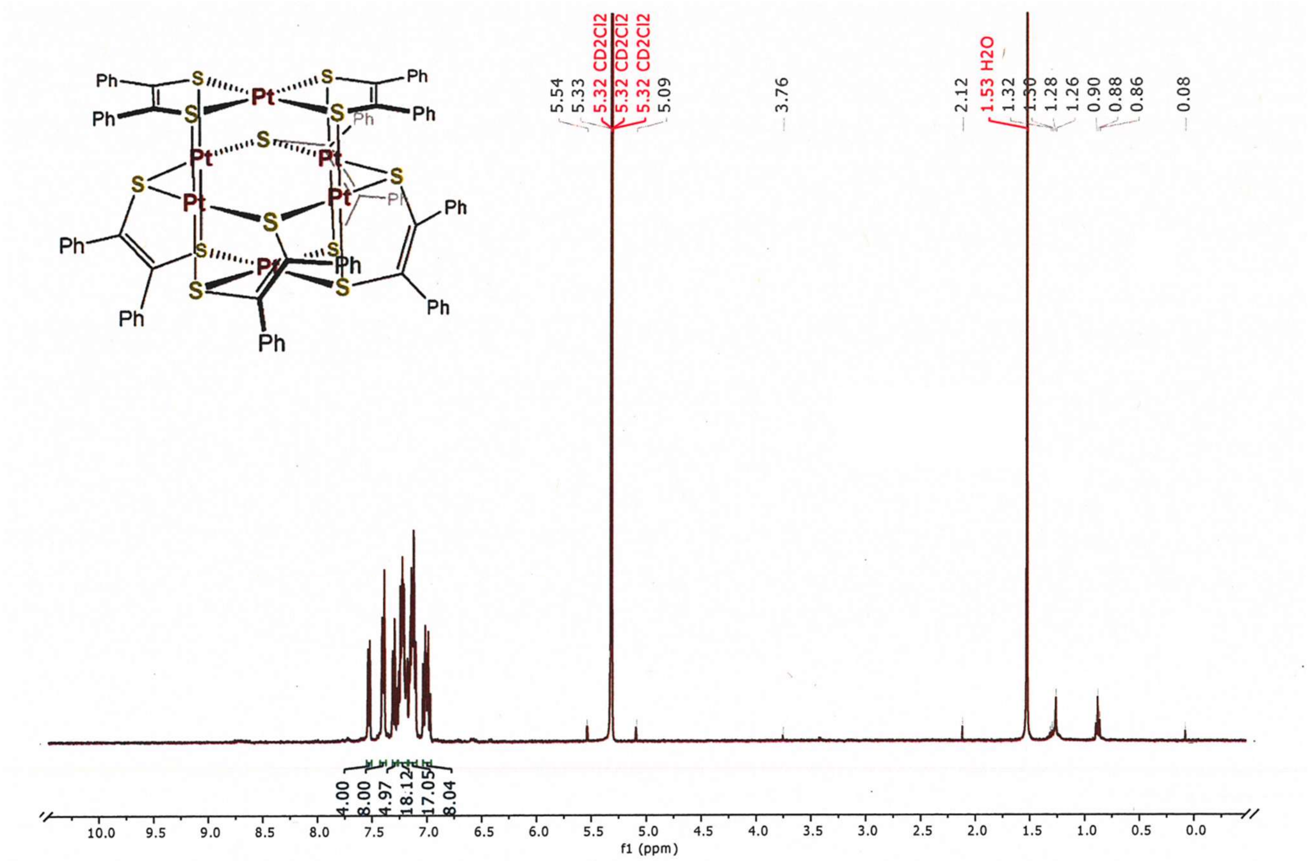

Figure S27. ^{1}H NMR spectrum of $[Pt_6(S_2C_2Ph_2)_6]$ in CD_2Cl_2, 0.0 - 10.0 ppm.

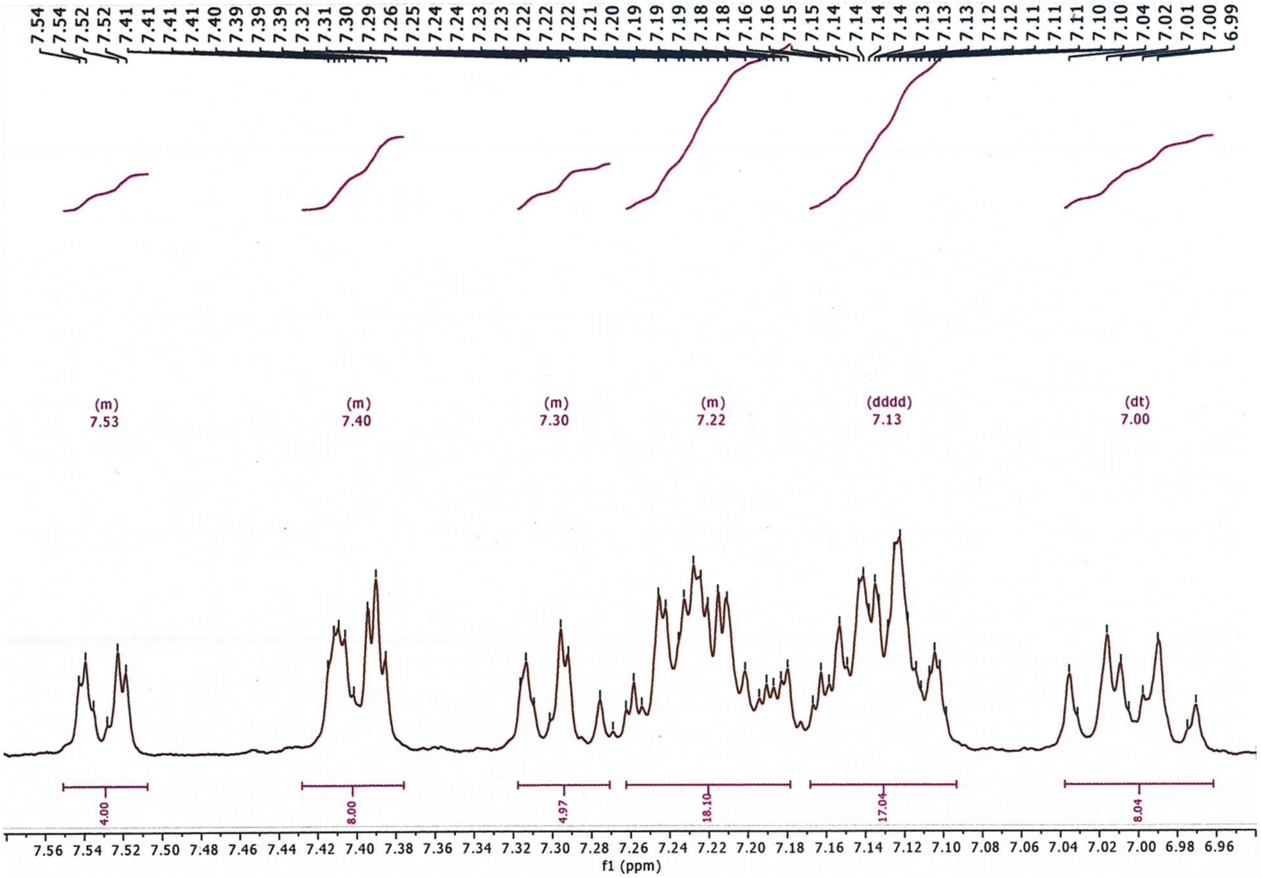

Figure S28. ^1H NMR spectrum of $[Pt_6(S_2C_2Ph_2)_6]$ in CD_2Cl_2, 6.94 - 7.58 ppm.

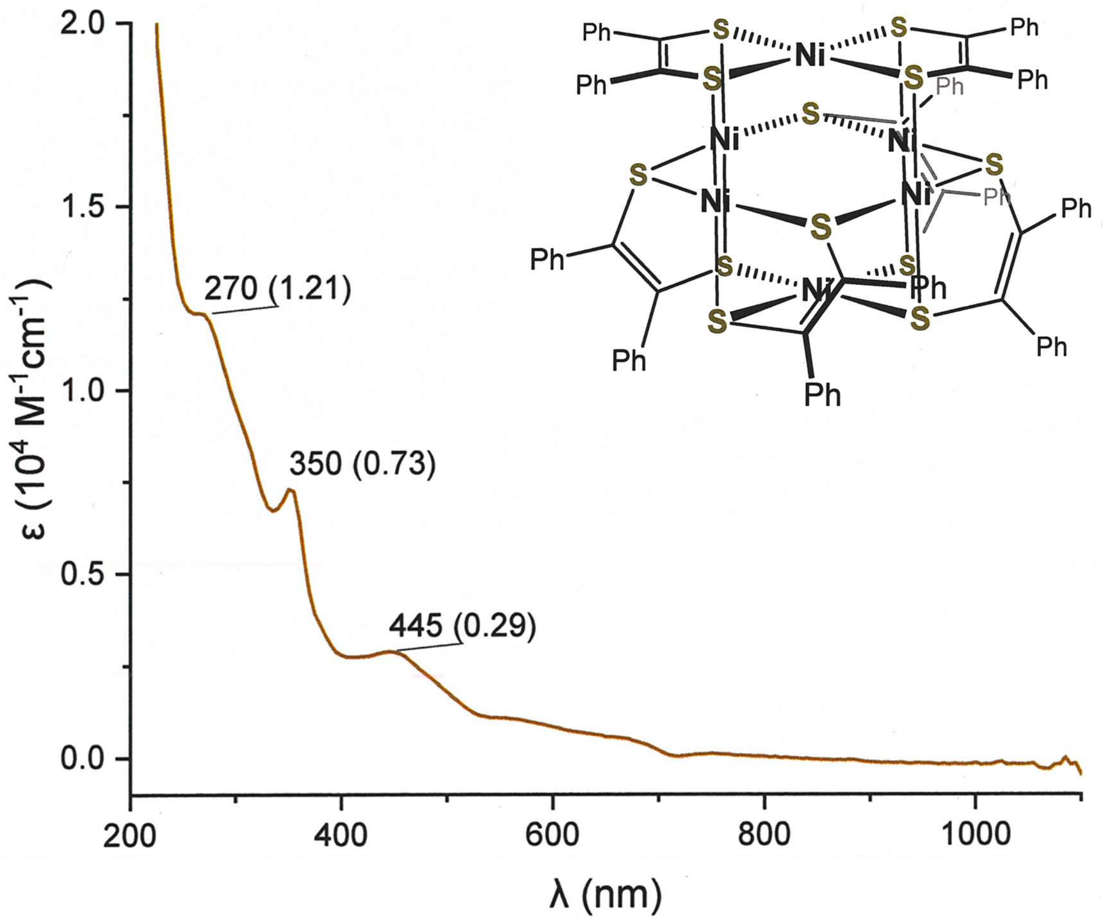

Figure S29. UV-vis spectrum of $[Ni_6(S_2C_2Ph_2)_6]$ in CH_2Cl_2 at $1.27x10^{-4}$ M concentration and 0.10 cm pathlength.

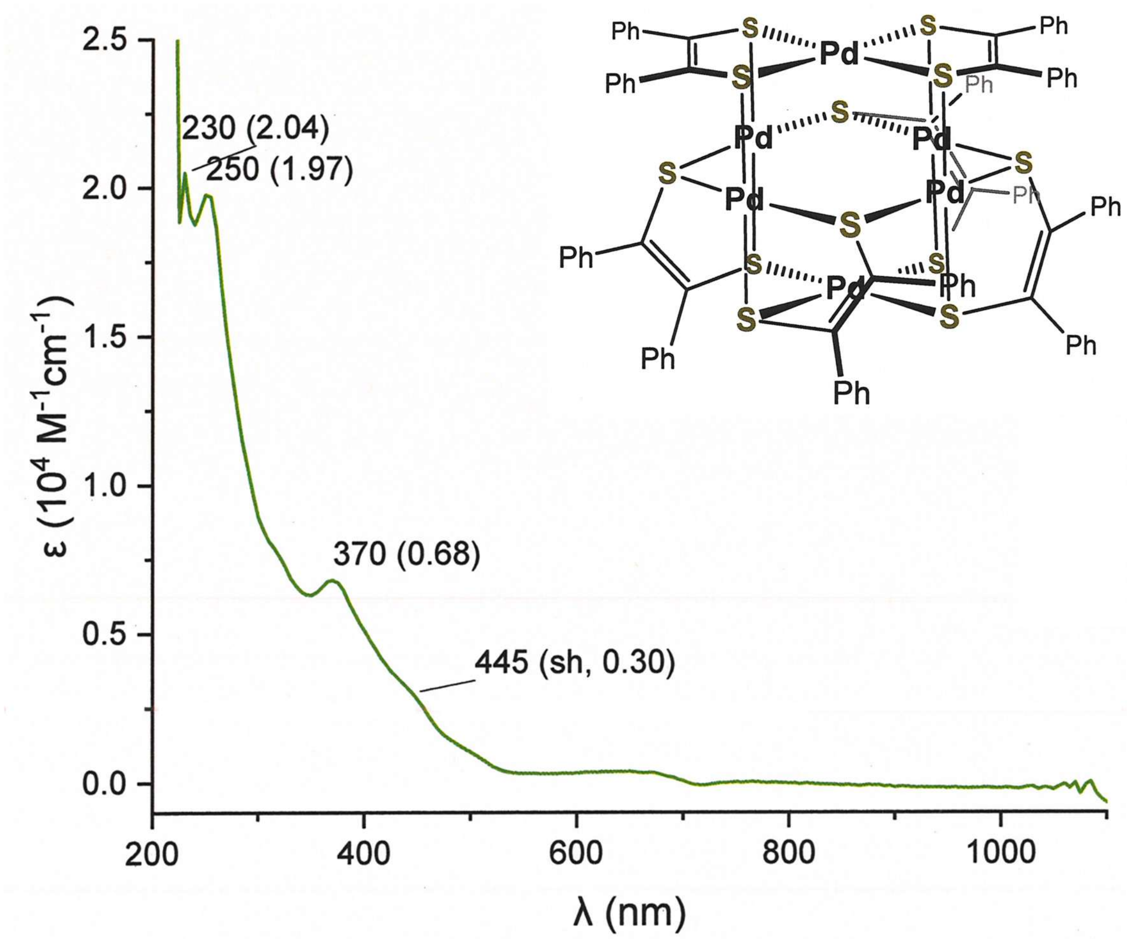

Figure S30. UV-vis spectrum of $[Pd_6(S_2C_2Ph_2)_6]$ in CH_2Cl_2 at $1.48x10^{-4}$ M concentration and 0.10 cm pathlength.

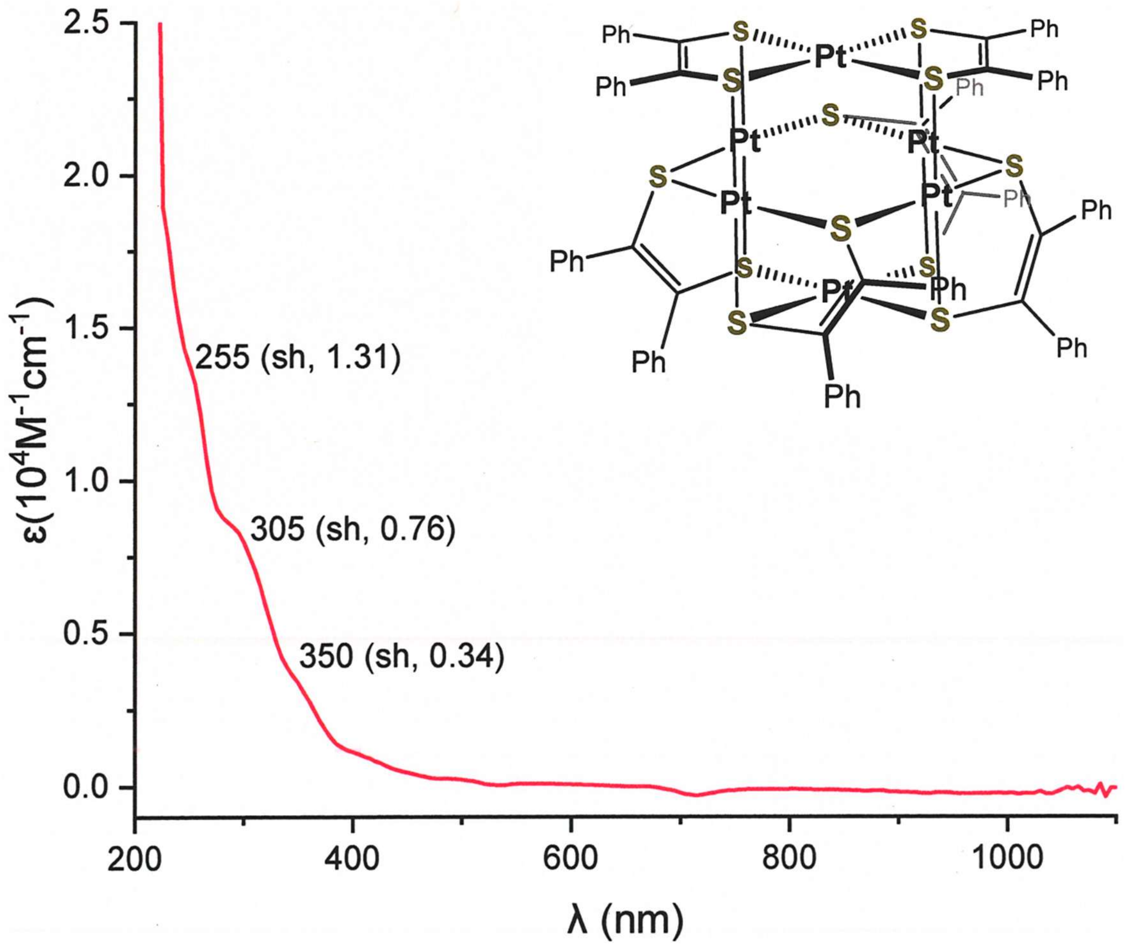

Figure S31. UV-vis spectrum of $[Pt_6(S_2C_2Ph_2)_6]$ in CH_2Cl_2 at $1.18x10^{-4}$ M concentration and 0.10 cm pathlength.

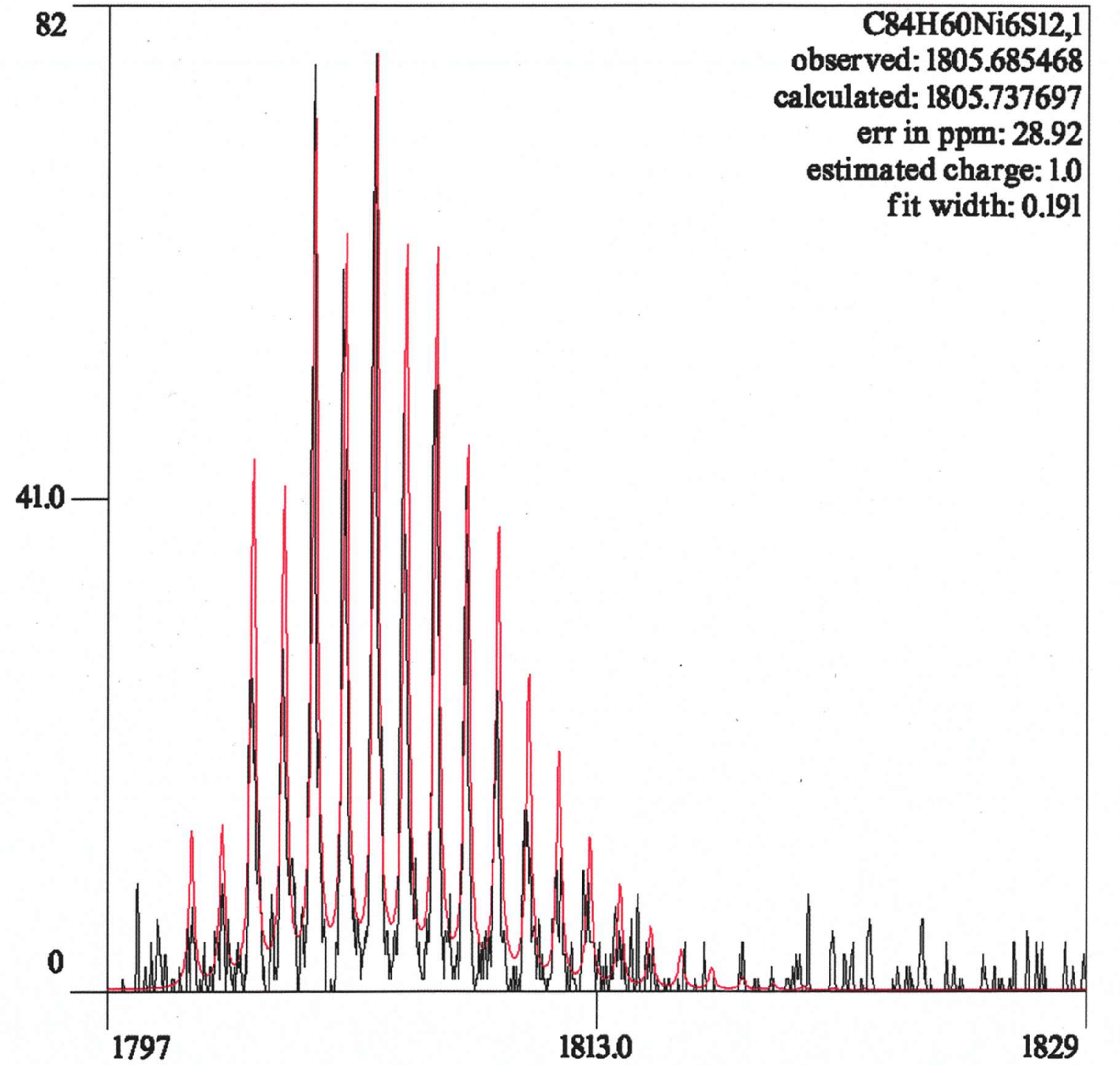

Figure S32. ESI mass spectrum of $[Ni_6(S_2C_2Ph_2)_6]$.

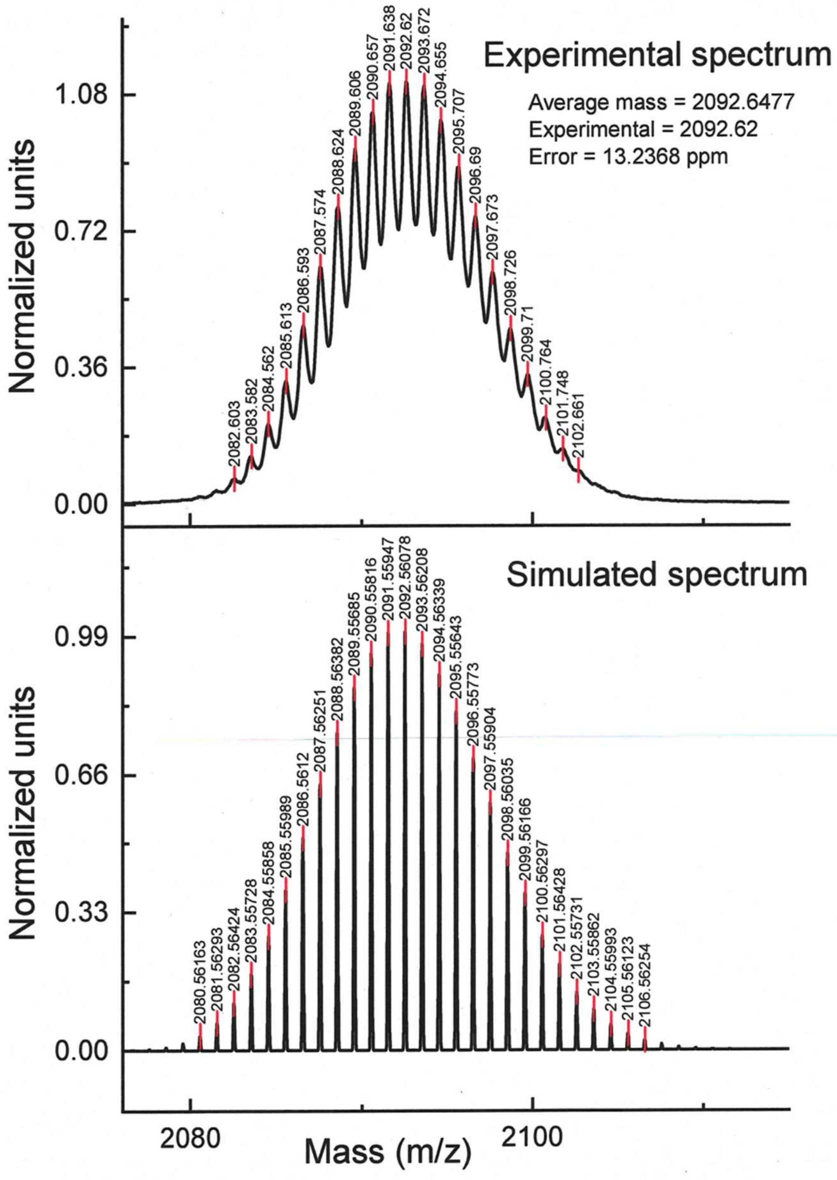

Figure S33. MALDI mass spectrum of $[Pd_6(S_2C_2Ph_2)_6]$ (top) with simulated spectrum shown at bottom.

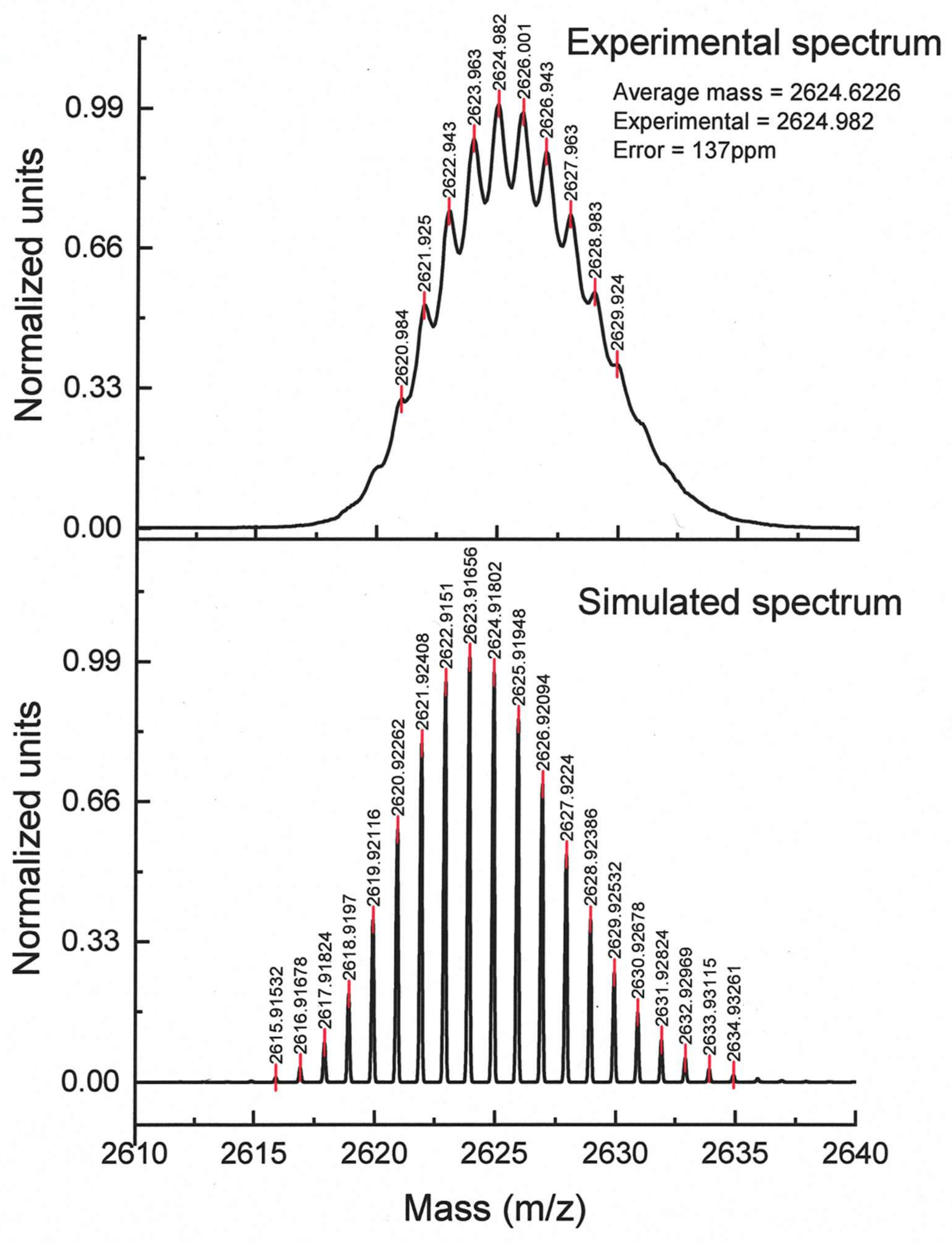

Figure S34. MALDI mass spectrum of $[Pt_6(S_2C_2Ph_2)_6]$ (top) with simulated spectrum shown at bottom.

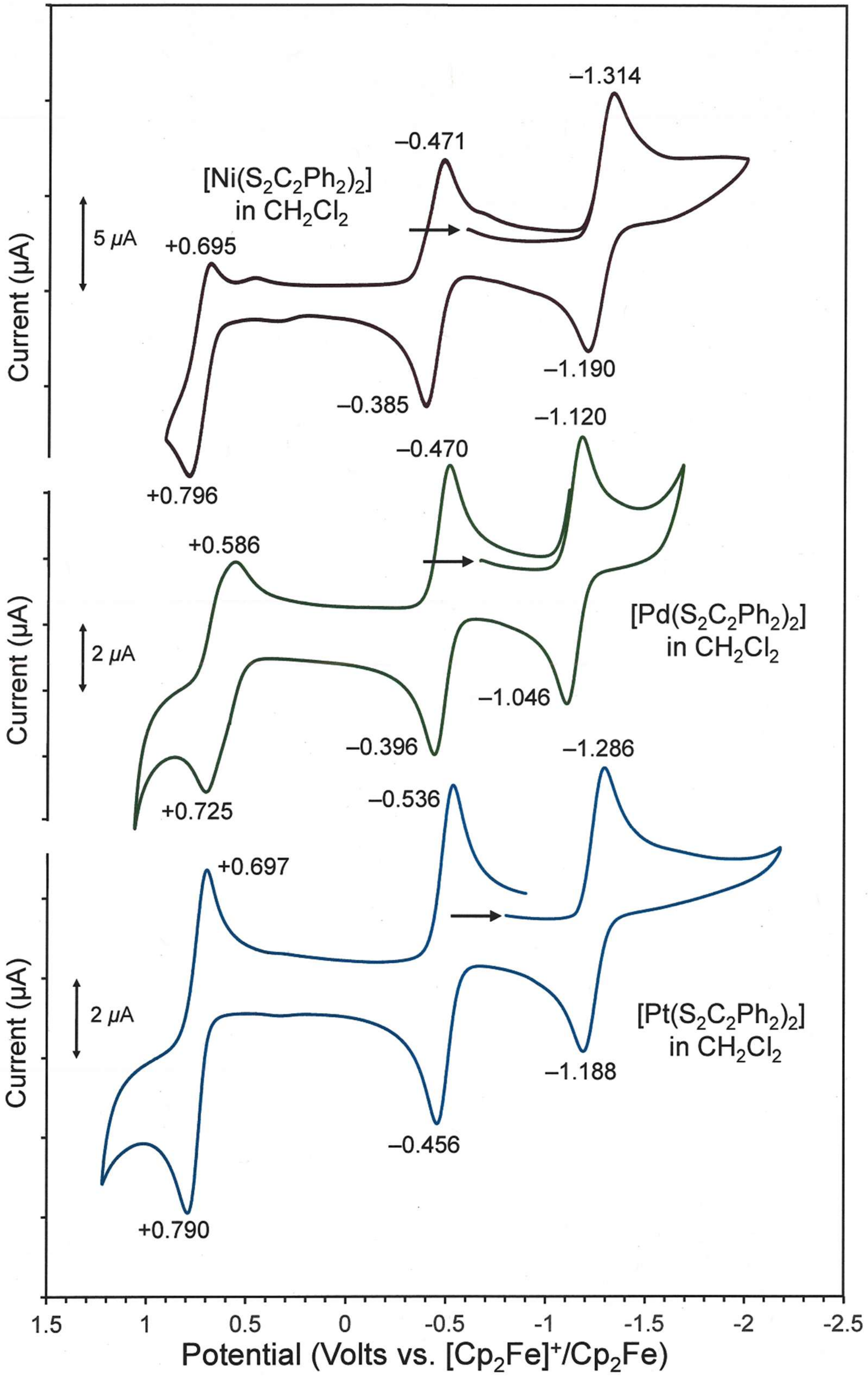

Figure S35. Cyclic voltammograms in CH_2Cl_2 for $[Ni(S_2C_2Ph_2)_2]$ (top), $[Pd(S_2C_2Ph_2)_2]$ (middle), and $[Pt(S_2C_2Ph_2)_2]$ (bottom) with $[^nBu_4N][PF_6]$ supporting electrolyte and Pt disk working electrode. The scan rate was 100 mV/s, Initial scan potentials are marked by the arrows.

Analysis Form

Address

Mikroanalytisches Laboratorium Kolbe
c/o Fraunhofer-Insitut UMSICHT
Building G - Osterfelderstr, 3
D-46047 Oberhausen

MIKROLAB
Mikroanalytisches Laboratorium Kolbe

Tel. +49 - (0)208 - 32502 www.mikro-lab.de
Fax +49 - (0)208 - 382314 info@mikro-lab.de

Order Order number: JPD218

Name: James P. Donahue

Address: Department of Chemistry, Tulane University
6400 Freret Street, Stern Hall Room 2015
New Orleans, Louisiana 70118-5698, USA

E-Mail-Address: donahue@tulane.edu

Sample name: JPD218

Elements to be determined: C, H, S

Other elements contained: Ni

Single determination [X] Double determination in case of deviation [ ] %

Double determination [ ] Absolute deviation for a double determination (Std. 1%)

Sample data

The sample is under: Argon [ ] Nitrogen [ ] Air [X]
Vacuum [ ] Other ______

	Yes	No		Yes	No
Moisture sensitive	[ ]	[X]	Explosive	[ ]	[X]
Hygroscopic	[ ]	[X]	Sublimated	[ ]	[X]
Inhomogeneous	[ ]	[X]	Volatile	[ ]	[X]

Molecular formula $C_{84}H_{60}S_{12}Ni_{6}$

Expected values in % wt

C: 55.85%
H: 3.35%
S: 21.30%
Ni: 19.50%

Molecular structure

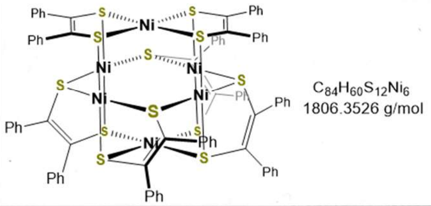

Special requests

	Yes	No		Yes	No
Handling under inert gas (Argon)	[ ]	[X]	Sample return	[ ]	[X]
Express treatment (max. 3 working days)	[ ]	[X]			
CHN surcharge	[ ]	[ ]			

(A combustion surcharge is strongly recommended when metals, silicon, fluorine or nitrogen containing ring compounds are present to avoid minor measurements)

Drying before analysis desired Yes [ ] No [X]

Conditions for drying ______ mbar ______ °C ______ Std.

Other wishes ______

Date 7/28/2023 Signature ______

Version 01/2020

Figure S36. Elemental analysis request form for $[Ni_6(S_2C_2Ph_2)_6]$ from the Kolbe Microanalytical Laboratory of Oberhausen, Germany.

MIKROLAB
Mikroanalytisches Laboratorium Kolbe

Professor James P. Donahue
Department of Chemistry
Tulane University
6400 Freret St.
New Orleans, Louisiana 70118-5698, USA

Address : Osterfelder Str. 3
D-46047 Oberhausen
Phone : +49 - (0)208 - 32502
Fax : +49 - (0)208 - 382314
Email : info@mikro-lab.de
Website : www.mikro-lab.de

Date : 16.08.2023

Sample Name	% C	% H	% N	% Cl	% S	% P						V2O5
JPD218	55,61	3,41			21,21							x

Kind regards

Patrick Springer

P.S.

Figure S37. Elemental analysis results for $[Ni_6(S_2C_2Ph_2)_6]$ from the Kolbe Microanalytical Laboratory of Oberhausen, Germany.

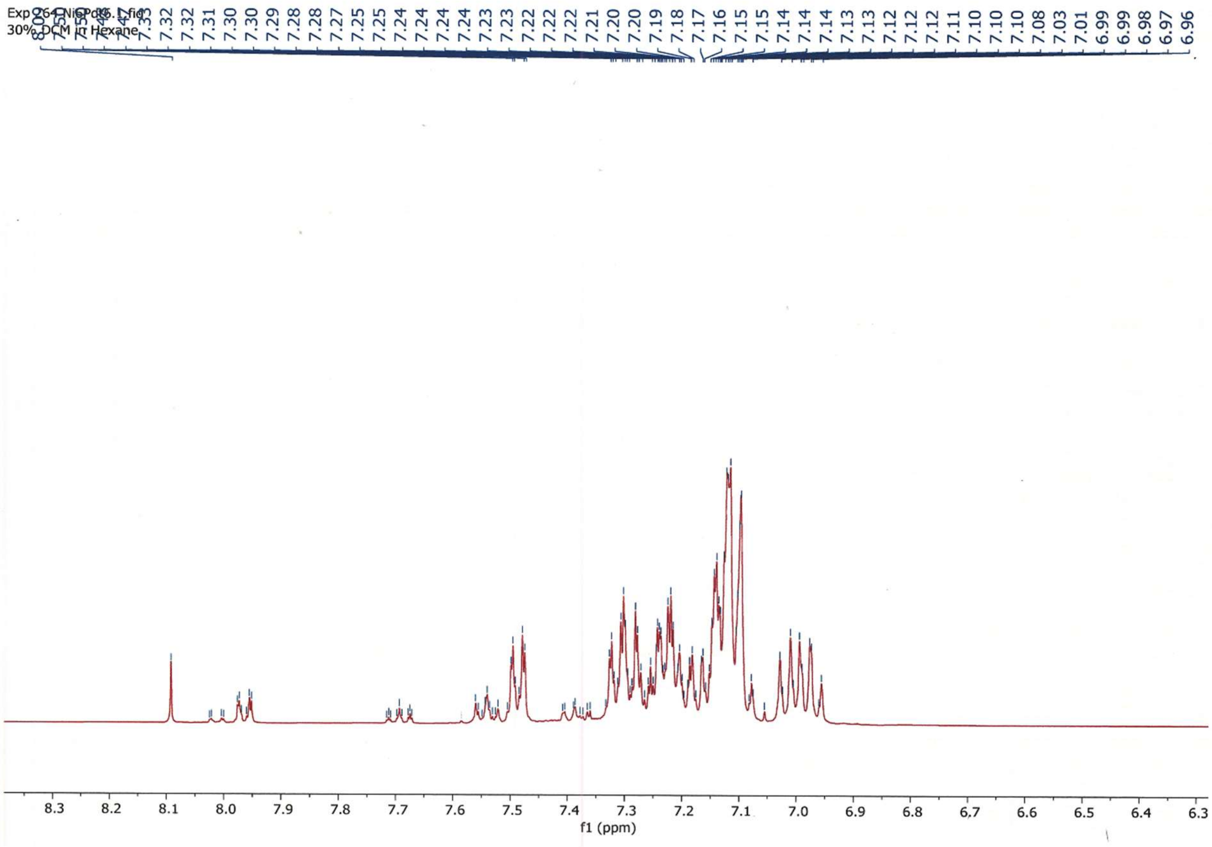

Figure S38. ^{1}H NMR spectrum (CD_2Cl_2) of column chromatographed [$Ni_6(S_2C_2Ph_2)_6$] without crystallization.

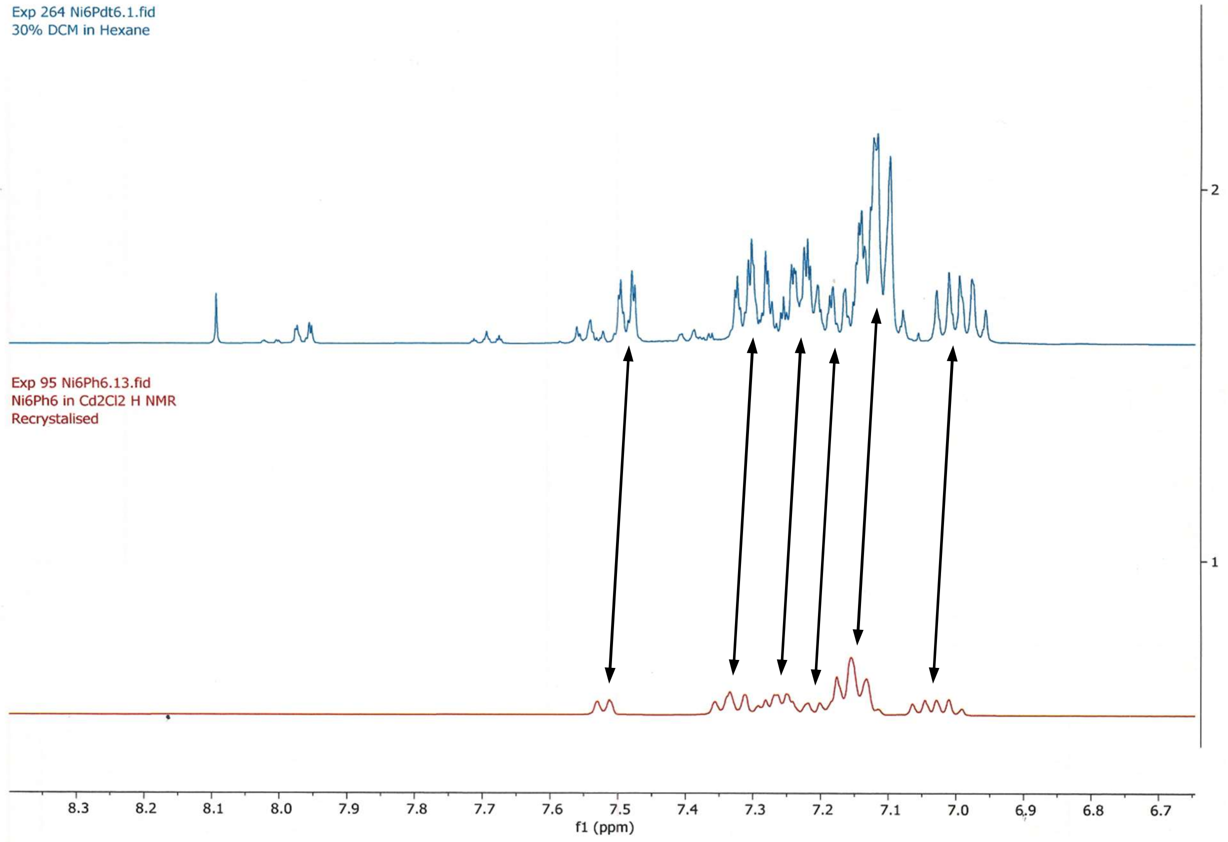

Figure S39. Comparison of the ^{1}H NMR spectra of column chromatographed $[Ni_6(S_2C_2Ph_2)_6]$ without crystallization (top) and single crystals of C_2-symmetric $[Ni_6(S_2C_2Ph_2)_6]$ (bottom). The arrows correlate signals belonging to the C_2-isomer in the two samples. Additional minor signals occur in the top spectrum at ~7.4 ppm and between 7.5-8.0 ppm, which are likely due to the S_6 isomer.

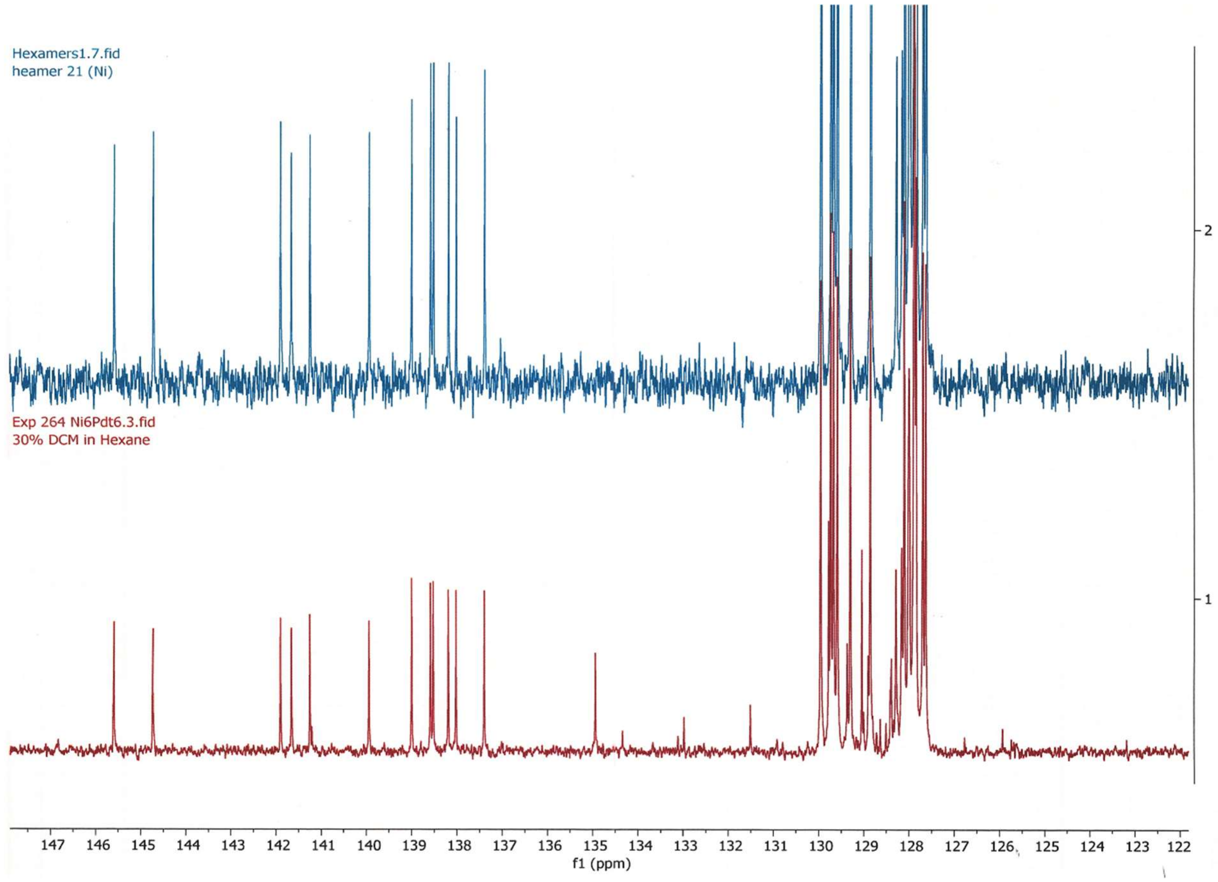

Figure S40. Comparison of the ^{13}C NMR spectra of column chromatographed $[Ni_6(S_2C_2Ph_2)_6]$ without crystallization (bottom) and single crystals of C_2-symmetric $[Ni_6(S_2C_2Ph_2)_6]$ (top) in the 122-148 ppm region. Additional minor signals occur in the bottom spectrum between 131-135 ppm, which are likely due to the S_6 isomer.

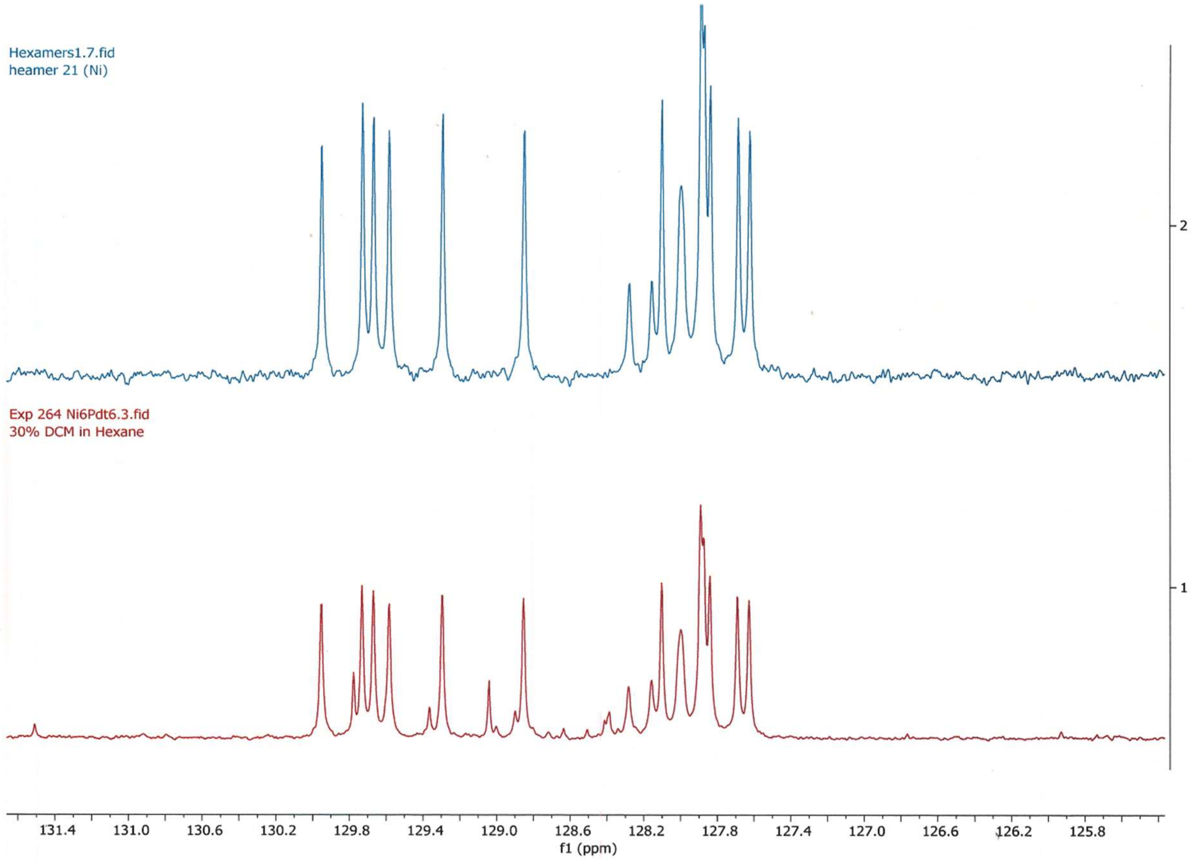

Figure S41. Comparison of the ^{13}C NMR spectra of column chromatographed $[Ni_6(S_2C_2Ph_2)_6]$ without crystallization (bottom) and single crystals of C_2-symmetric $[Ni_6(S_2C_2Ph_2)_6]$ (top) in the 125.4-131.5 ppm region. Additional minor signals occur in the bottom spectrum, which are likely due to the S_6 isomer.

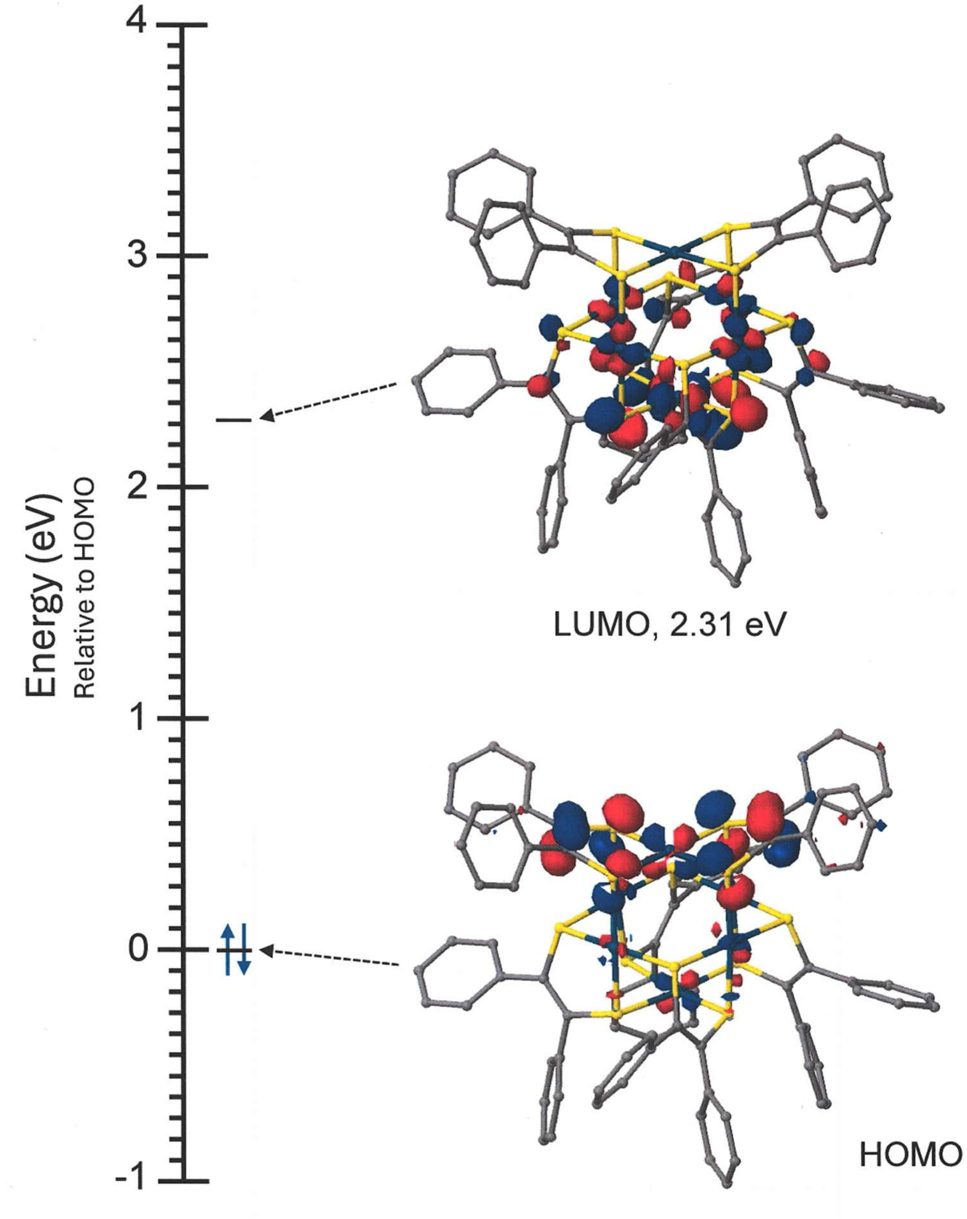

Figure S42. MO energy level diagram for [$Pd_6(S_2C_2Ph_2)_6$]. The orbital images are rendered at the 0.05 contour level.

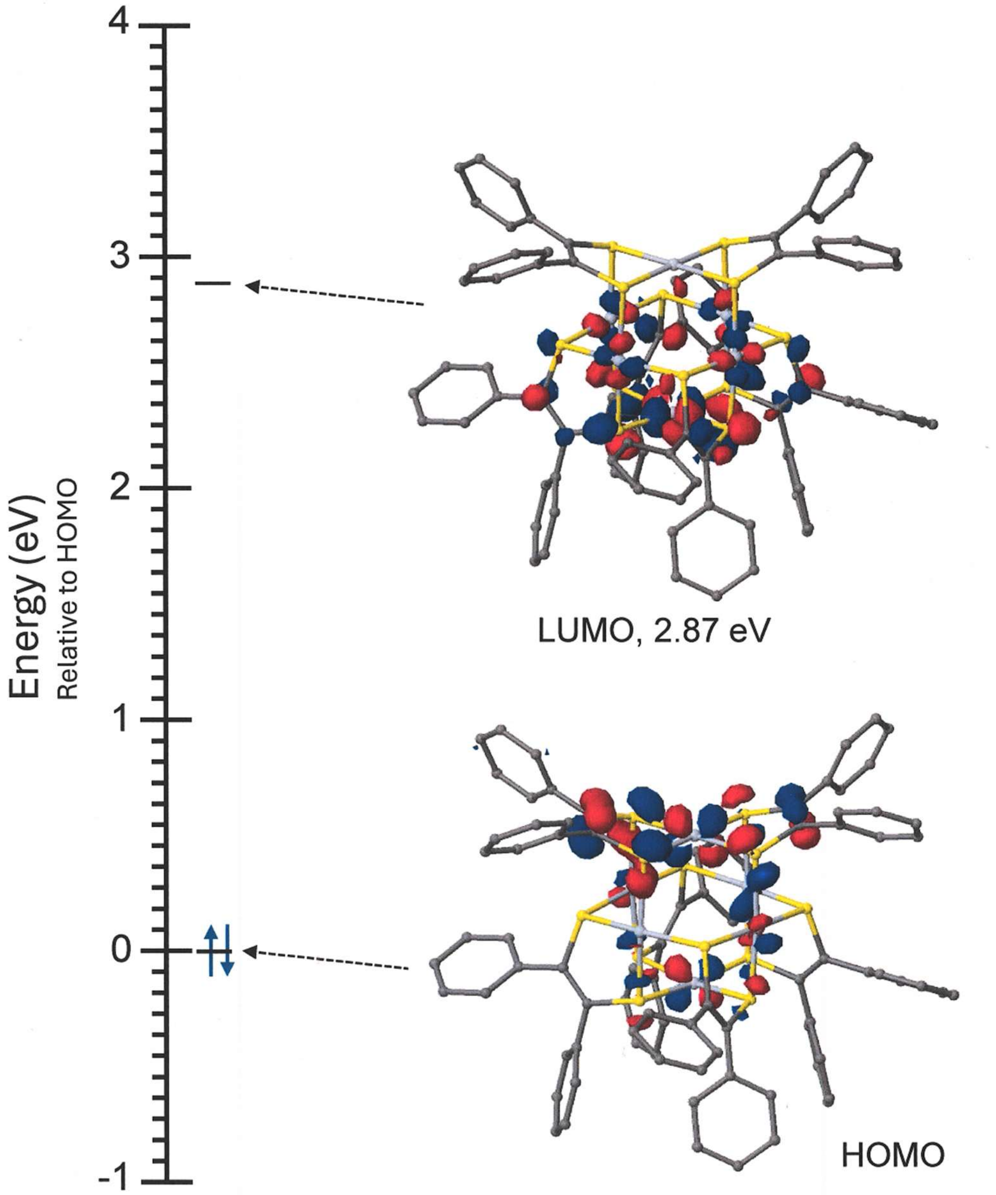

Figure S43. MO energy level diagram for $[Pt_6(S_2C_2Ph_2)_6]$. The orbital images are rendered at the 0.05 contour level.

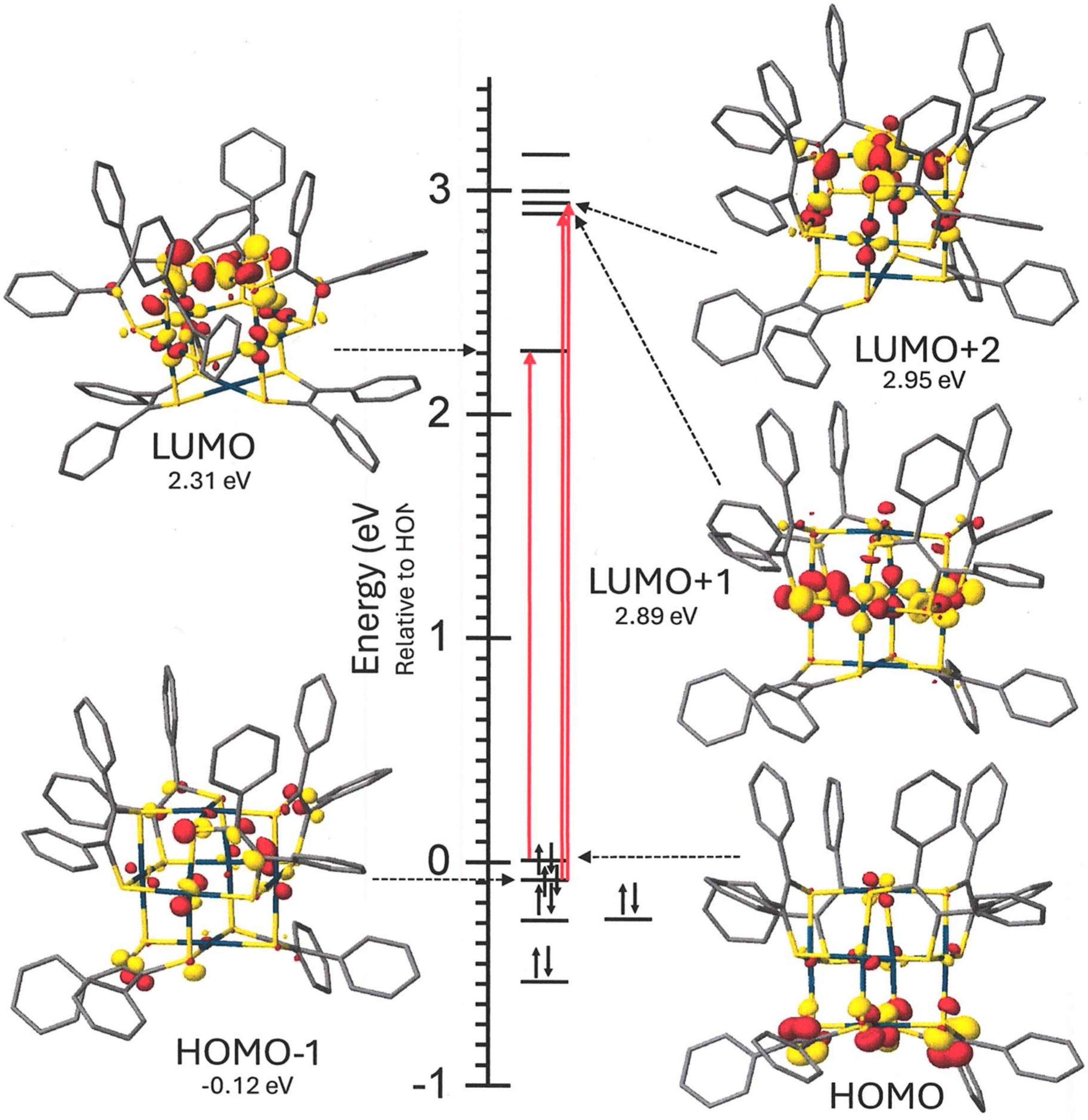

Figure S44. MO energy level diagram for $[Pd_6(S_2C_2Ph_2)_6]$ illustrating the **3** most important electronic excitations on the basis of computed oscillator strength. Compound **2** is reoriented top-for-bottom in this figure compared to this **Figure S42**.

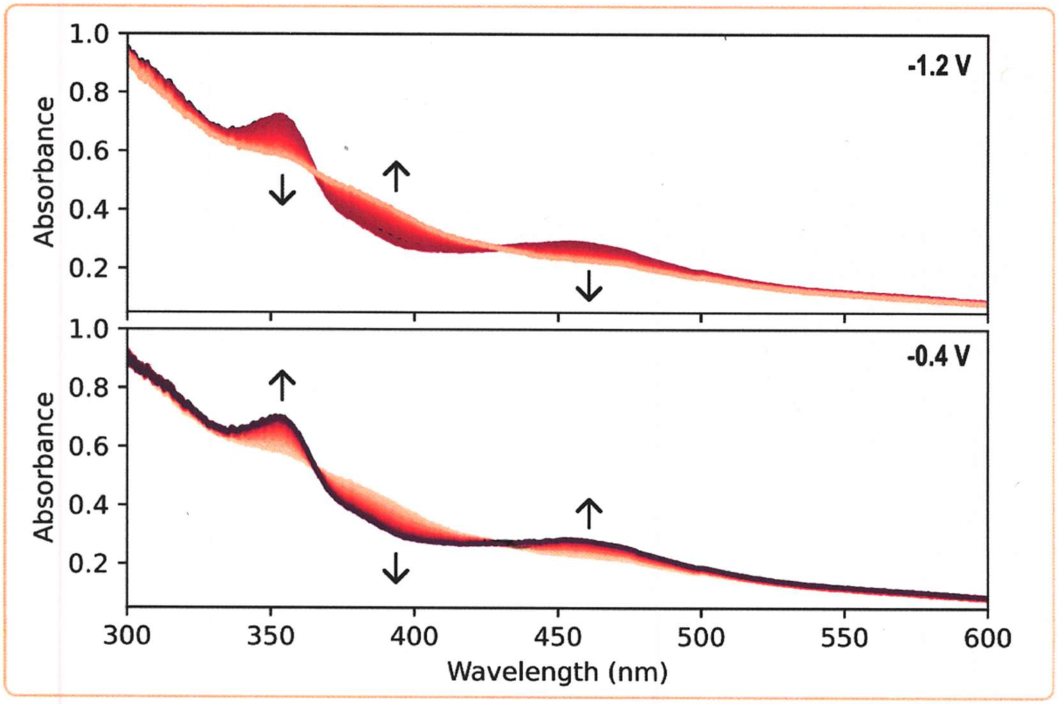

Figure S45. Spectroscopic characterization of $[Ni_6(S_2C_2Ph_2)_6]^{1-}$ upon generation at a poised potential of –1.2 V vs. Ag^+/Ag and reoxidation at –0.4 V. The absorption maxima for $[Ni_6(S_2C_2Ph_2)_6]^0$ are smoothed out to a near-featureless taper.

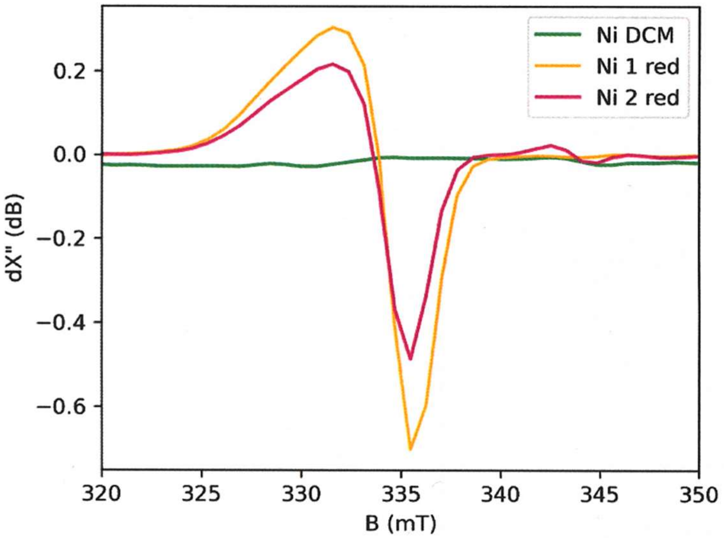

Figure S46. X-band EPR spectra (CH_2Cl_2, 20 K) of $[Ni_6(S_2C_2Ph_2)_6]^0$ (▬) and $[Ni_6(S_2C_2Ph_2)_6]^{1-}$ (▬). The spectrum in red is a mixture of $[Ni_6(S_2C_2Ph_2)_6]^{1-}$ and $[Ni_6(S_2C_2Ph_2)_6]^{2-}$ due to unsuccessful separation of anolyte and catholyte by a porous glass frit.

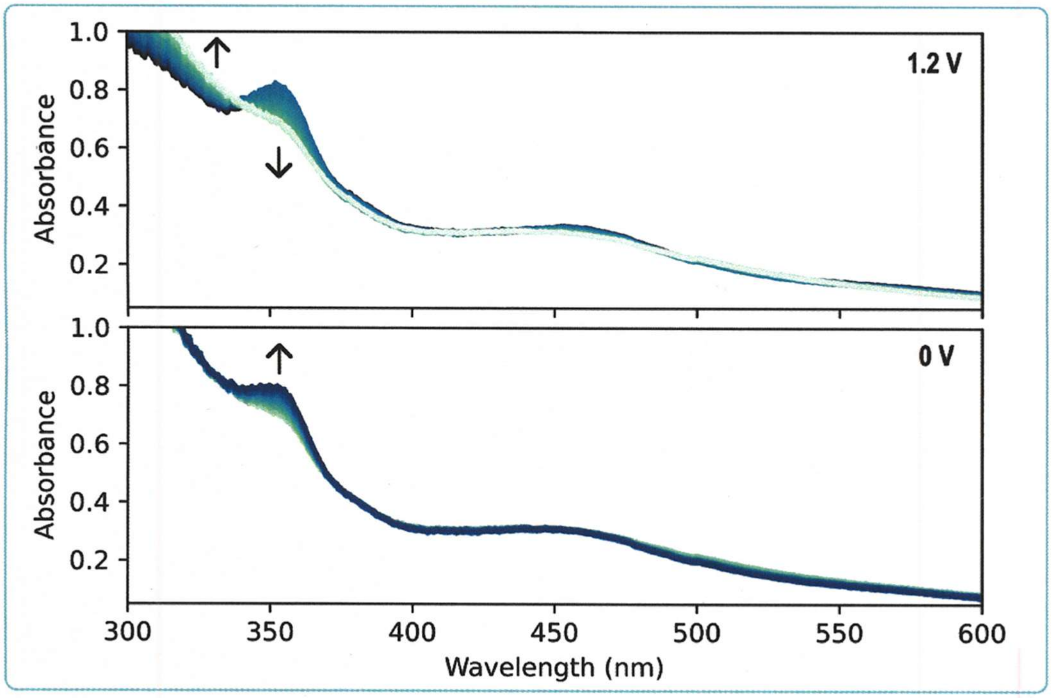

Figure S47. Spectroscopic characterization of $[Ni_6(S_2C_2Ph_2)_6]^{1+}$ upon generation at a poised potential of +1.2 V vs. Ag^+/Ag and re-reduction at 0 V. The absorption maximum at ~350 nm decreases reversibly.

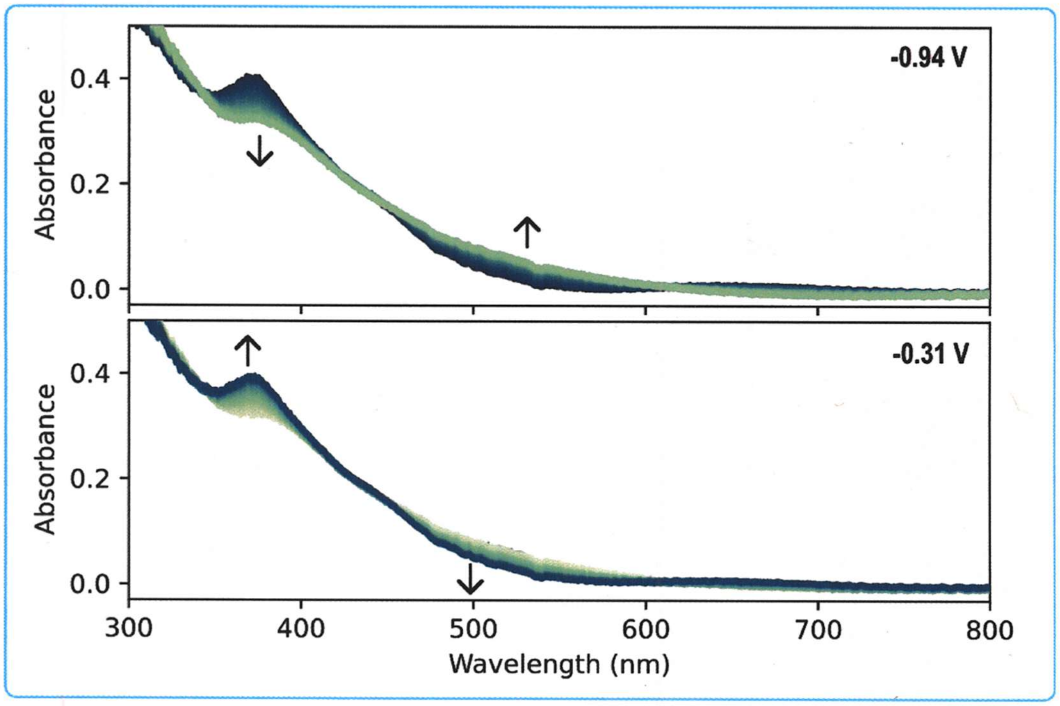

Figure S48. Spectroscopic characterization of $[Pd_6(S_2C_2Ph_2)_6]^{1-}$ upon reduction at a poised potential of –0.94 V vs. Ag^+/Ag and reoxidation at –0.31 V.

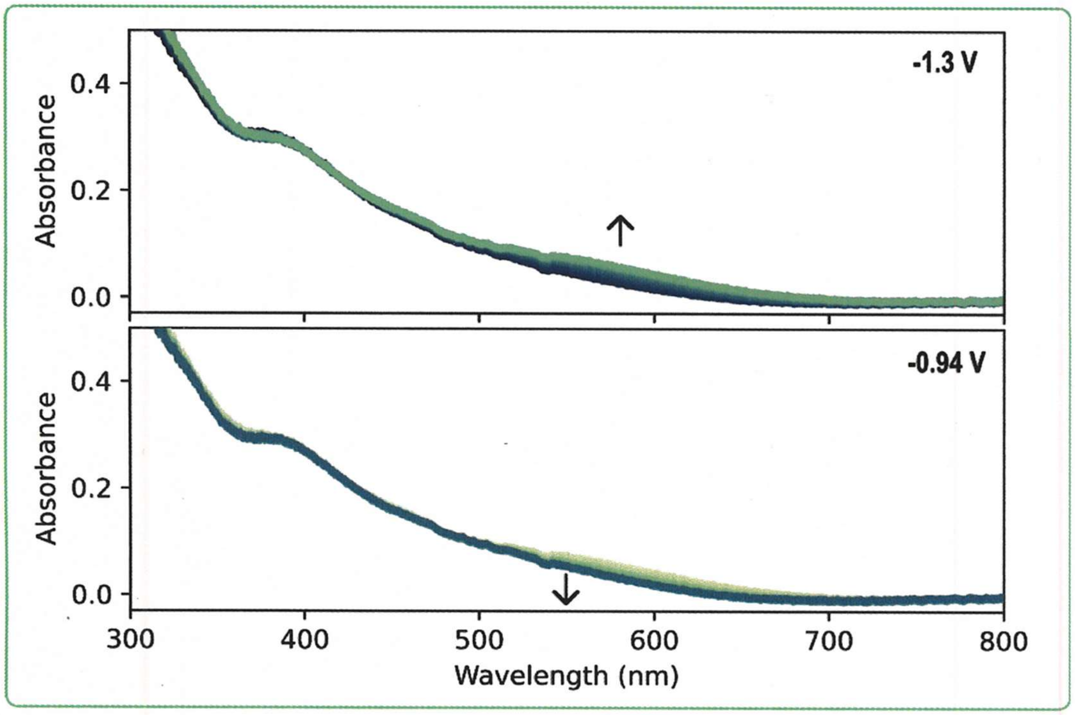

Figure S49. Spectroscopic characterization of $[Pd_6(S_2C_2Ph_2)_6]^{2-}$ upon reduction at a poised potential of –1.31 V vs. Ag^+/Ag and reoxidation at –0.94 V.

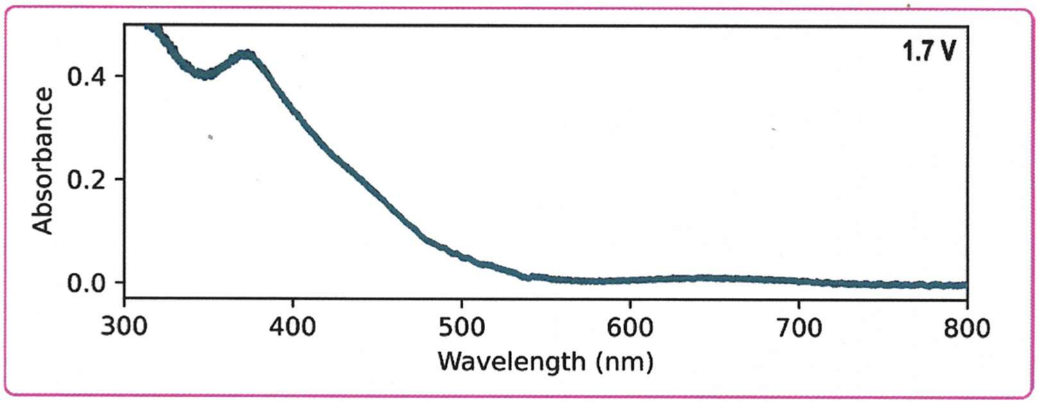

Figure S50. Spectroscopic characterization of $[Pd_6(S_2C_2Ph_2)_6]^{1+}$ upon generation at a poised potential of +1.7 V vs. Ag^+/Ag.

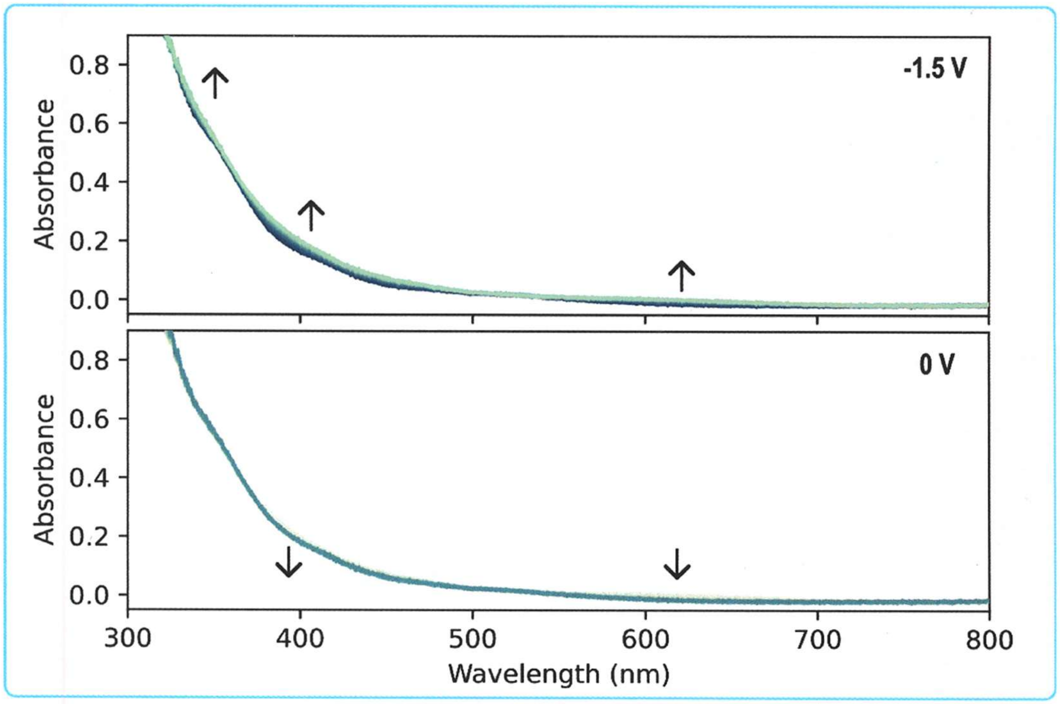

Figure S51. Spectroscopic characterization of $[Pt_6(S_2C_2Ph_2)_6]^{1-}$ upon reduction at a poised potential of –1.5 V vs. Ag^+/Ag and reoxidation at 0.0 V.

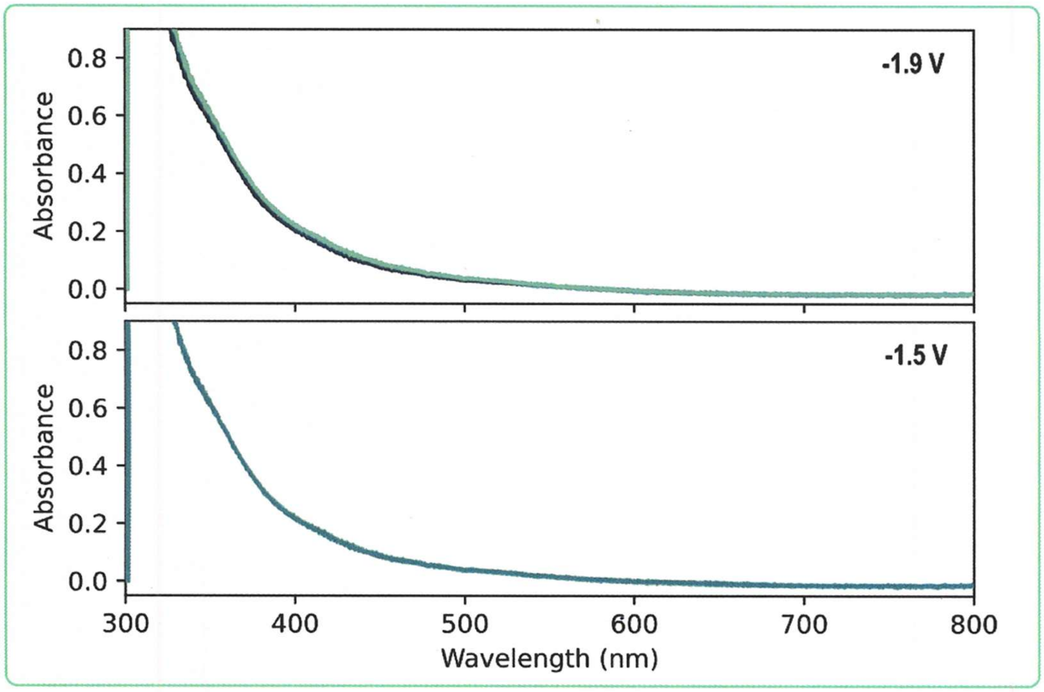

Figure S52. Spectroscopic characterization of $[Pt_6(S_2C_2Ph_2)_6]^{2-}$ upon reduction at a poised potential of –1.9 V vs. Ag^+/Ag and reoxidation at –1.5 V.

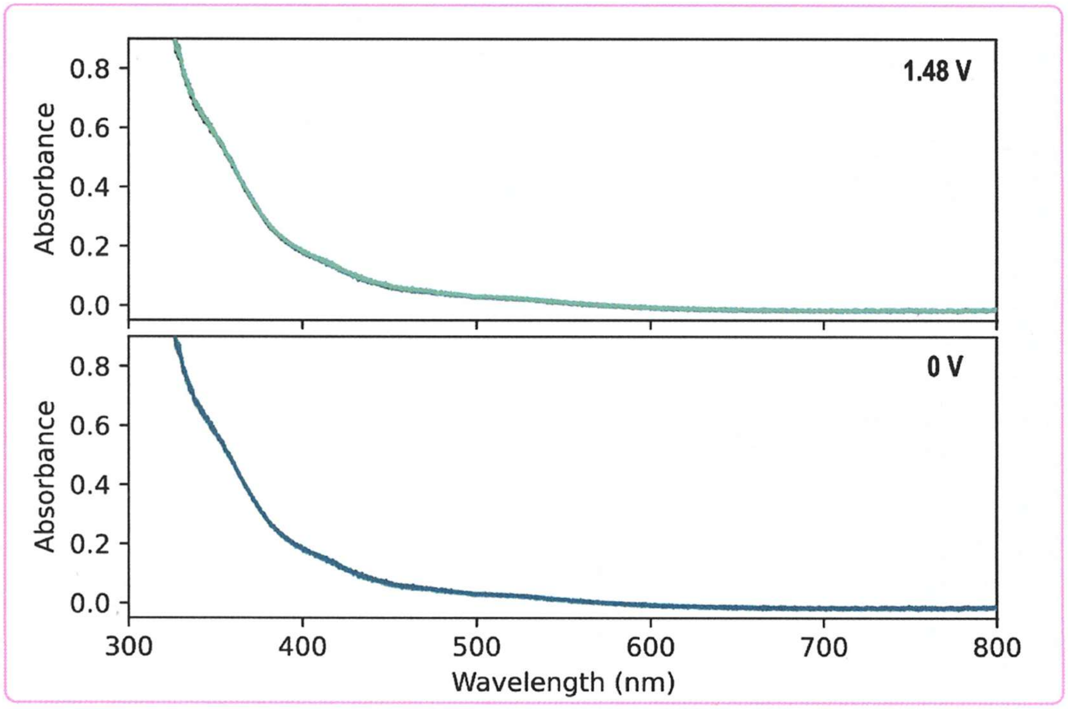

Figure S53. Spectroscopic characterization of $[Pt_6(S_2C_2Ph_2)_6]^{1+}$ upon oxidation at a poised potential of +1.48 V vs. Ag^+/Ag and re-reduction at 0.0 V.

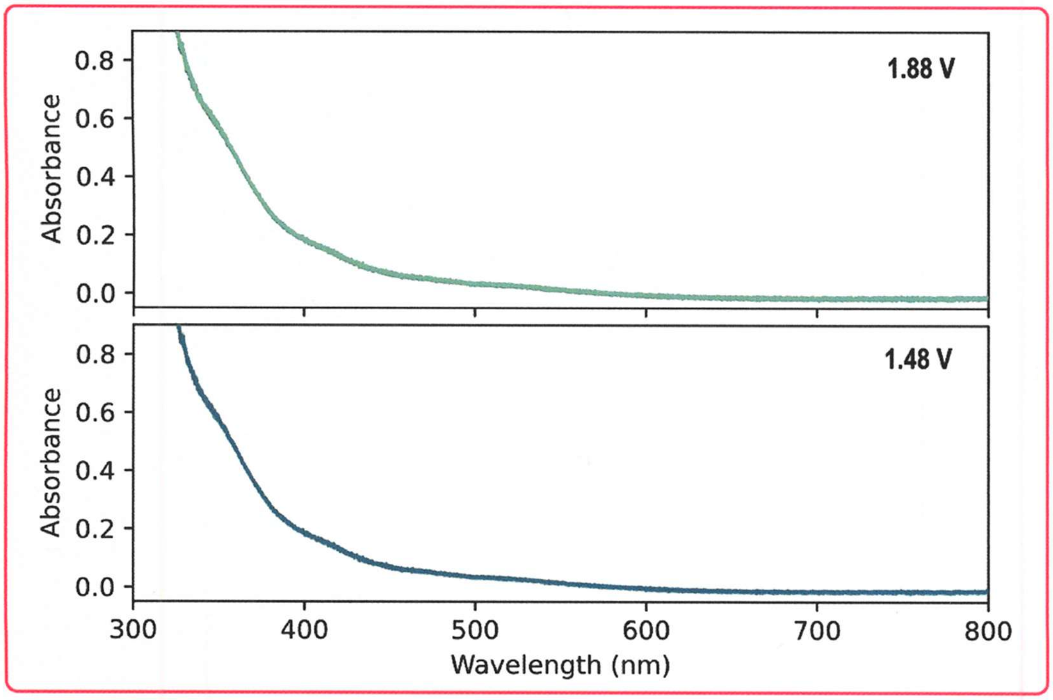

Figure S54. Spectroscopic characterization of $[Pt_6(S_2C_2Ph_2)_6]^{2+}$ upon oxidation at a poised potential of +1.88 V vs. Ag^+/Ag and re-reduction at +1.48 V.

Table S3. Final Atomic Coordinates for Optimized C_2 $[Ni_6(C_2S_2Ph_2)_6]$.

Atomic Number	Coordinates (Angstroms) X	Y	Z
28	0.014268	-2.883704	0.027819
28	-0.001843	1.668718	-0.024447
28	1.648196	-0.662778	-1.549598
28	-1.647142	-0.666620	-1.548815
28	1.664103	-0.589366	1.562896
28	-1.631086	-0.626796	1.570978
16	-1.701539	1.570176	-1.574759
16	-3.274728	-0.587307	-0.030516
16	-1.595903	-2.935472	1.578975
16	-1.570309	-2.972244	-1.549713
16	-1.544407	1.599871	1.701548
16	-0.023910	-0.692100	3.134144
16	3.292876	-0.558372	0.039729
16	1.707780	1.647804	1.524209
16	1.537403	1.557460	-1.748561
16	0.049135	-0.789557	-3.113675
16	1.594706	-2.891569	1.613595
16	1.633650	-2.975916	-1.513899
6	-3.247114	2.029472	-0.788484
6	-3.944737	1.079889	-0.124392
6	-3.039573	-3.535499	0.689599
6	-3.015172	-3.580619	-0.663826
6	-0.763018	1.927706	3.286324
6	-0.115780	0.924180	3.922585
6	3.247962	2.082840	0.710563
6	3.949209	1.114761	0.078524
6	0.759755	1.828164	-3.346367
6	0.089330	0.809829	-3.937351
6	3.058609	-3.501221	0.764191
6	3.056830	-3.575476	-0.588609
6	-3.606170	3.465646	-0.917924
6	-3.880557	4.250469	0.214478
1	-3.829462	3.796519	1.202350
6	-4.195939	5.601392	0.081244
1	-4.397362	6.196949	0.973719
6	-4.245401	6.193400	-1.182143
1	-4.493276	7.251794	-1.283613
6	-3.971516	5.424867	-2.314592
1	-4.008242	5.877947	-3.307195
6	-3.647890	4.075037	-2.184219
1	-3.435571	3.478270	-3.072096
6	-5.266760	1.259095	0.526272
6	-6.332183	1.864383	-0.161385
1	-6.180929	2.214806	-1.182252
6	-7.577561	2.010787	0.446962

Table S3, Continued. Final Atomic Coordinates for Optimized C_2 [$Ni_6(C_2S_2Ph_2)_6$].

Atomic Number	Coordinates (Angstroms) X	Y	Z
1	-8.394446	2.479706	-0.105193
6	-7.783522	1.554831	1.750063
1	-8.760147	1.669710	2.224340
6	-6.734624	0.944683	2.440272
1	-6.885287	0.581174	3.458588
6	-5.489038	0.791421	1.833497
1	-4.673743	0.312050	2.376644
6	-4.189087	-3.898779	1.558688
6	-5.478645	-3.401765	1.299881
1	-5.636063	-2.742013	0.448202
6	-6.546686	-3.734746	2.130687
1	-7.539131	-3.333703	1.916088
6	-6.349711	-4.567336	3.234190
1	-7.188366	-4.827113	3.883306
6	-5.072832	-5.060716	3.505845
1	-4.907443	-5.710895	4.367113
6	-4.000349	-4.724663	2.681041
1	-3.004450	-5.110326	2.901508
6	-4.083079	-4.132736	-1.536193
6	-4.554486	-3.405863	-2.643153
1	-4.145636	-2.415567	-2.847366
6	-5.542783	-3.934943	-3.471142
1	-5.902149	-3.351325	-4.320967
6	-6.068323	-5.203289	-3.218624
1	-6.838491	-5.619037	-3.871262
6	-5.597012	-5.939640	-2.130676
1	-5.993324	-6.937184	-1.930855
6	-4.613082	-5.411655	-1.296746
1	-4.244491	-5.995016	-0.452962
6	-0.900676	3.328878	3.765030
6	-2.173060	3.917779	3.870857
1	-3.057131	3.330820	3.617571
6	-2.315270	5.232425	4.312747
1	-3.312699	5.667959	4.399760
6	-1.188680	5.987518	4.642644
1	-1.298962	7.019107	4.982522
6	0.079920	5.415506	4.534031
1	0.967232	6.000463	4.783797
6	0.224850	4.098276	4.101546
1	1.216344	3.657843	4.013197
6	0.522275	1.002180	5.260838
6	-0.168953	1.542562	6.358141
1	-1.183058	1.917479	6.221648
6	0.427580	1.594416	7.616685
1	-0.127601	2.013074	8.458420

Table S3, Continued. Final Atomic Coordinates for Optimized C_2 $[Ni_6(C_2S_2Ph_2)_6]$.

Atomic Number	Coordinates (Angstroms) X	Y	Z
6	1.722212	1.107894	7.803631
1	2.187280	1.148342	8.790523
6	2.415319	0.560825	6.722276
1	3.426835	0.173192	6.857993
6	1.820012	0.500717	5.463688
1	2.364188	0.067078	4.623675
6	3.609238	3.523159	0.772590
6	3.695178	4.181965	2.010922
1	3.513528	3.620582	2.928406
6	4.028842	5.533939	2.076361
1	4.102517	6.026136	3.048158
6	4.268604	6.254564	0.905201
1	4.525007	7.314606	0.955929
6	4.176870	5.612466	-0.331117
1	4.354973	6.169163	-1.253145
6	3.851706	4.258591	-0.399897
1	3.771703	3.763349	-1.366619
6	5.270226	1.286550	-0.577352
6	6.342148	1.876152	0.113031
1	6.195158	2.218997	1.137193
6	7.587211	2.017994	-0.497205
1	8.409533	2.475064	0.056787
6	7.785155	1.574001	-1.805451
1	8.760879	1.686142	-2.282113
6	6.728486	0.981752	-2.499398
1	6.872672	0.633552	-3.524069
6	5.483721	0.832474	-1.890250
1	4.659413	0.373054	-2.437087
6	0.990029	3.179930	-3.917396
6	1.507622	3.328902	-5.215405
1	1.719144	2.441477	-5.812321
6	1.756105	4.596000	-5.739708
1	2.161251	4.691104	-6.749118
6	1.497009	5.736848	-4.977909
1	1.693160	6.728663	-5.389958
6	0.991913	5.600705	-3.683596
1	0.787107	6.485384	-3.077685
6	0.746185	4.334687	-3.153672
1	0.352883	4.235328	-2.141336
6	-0.695320	0.879875	-5.195048
6	-0.618897	-0.154124	-6.145371
1	0.038847	-1.004044	-5.962131
6	-1.369168	-0.099596	-7.319244
1	-1.288774	-0.908765	-8.047504
6	-2.218889	0.980232	-7.561370

Table S3, Continued. Final Atomic Coordinates for Optimized C_2 $[Ni_6(C_2S_2Ph_2)_6]$.

Atomic Number	Coordinates (Angstroms) X	Y	Z
1	-2.809735	1.019893	-8.478533
6	-2.312753	2.007734	-6.619851
1	-2.981814	2.852319	-6.796221
6	-1.560965	1.959637	-5.448349
1	-1.643946	2.759441	-4.714012
6	4.194915	-3.836898	1.660506
6	3.991161	-4.624421	2.807499
1	2.992449	-5.002771	3.027813
6	5.052727	-4.932470	3.657169
1	4.875767	-5.553504	4.537439
6	6.333273	-4.448839	3.385638
1	7.163366	-4.686689	4.053834
6	6.545102	-3.654032	2.257131
1	7.541143	-3.262267	2.041794
6	5.488200	-3.348743	1.402082
1	5.656686	-2.718282	0.530224
6	4.130781	-4.165019	-1.428212
6	4.613686	-3.488792	-2.561659
1	4.210037	-2.506718	-2.810063
6	5.604654	-4.056666	-3.360145
1	5.972387	-3.511849	-4.231910
6	6.122048	-5.315663	-3.051111
1	6.894138	-5.762357	-3.680554
6	5.639024	-6.003345	-1.936720
1	6.028036	-6.993936	-1.692931
6	4.652249	-5.436075	-1.132756
1	4.274114	-5.982425	-0.268805

Table S4. Final Atomic Coordinates for Optimized C_2 $[Pd_6(C_2S_2Ph_2)_6]$.

Atomic Numbers	Coordinates (Angstroms) X	Y	Z
46	0.002640	-3.016871	0.009817
46	0.007985	1.875396	-0.021261
46	1.747985	-0.634476	-1.680492
46	-1.793723	-0.629096	-1.641757
46	1.805762	-0.567514	1.650027
46	-1.732978	-0.594948	1.699763
16	-1.835927	1.730926	-1.595420
16	-3.470098	-0.487556	0.001605
16	-1.724028	-3.033617	1.622819
16	-1.728324	-3.067297	-1.603133
16	-1.552517	1.746501	1.853505
16	0.007161	-0.642442	3.303508
16	3.487166	-0.480333	0.003313
16	1.861963	1.790866	1.553794
16	1.563132	1.702806	-1.898124
16	0.013316	-0.728064	-3.283367
16	1.717249	-3.000571	1.639099
16	1.750214	-3.078962	-1.584733
6	-3.346237	2.166148	-0.724560
6	-4.052230	1.218130	-0.062446
6	-3.179601	-3.558616	0.699182
6	-3.167791	-3.605053	-0.656197
6	-0.683720	2.025919	3.404865
6	-0.042379	1.009325	4.030821
6	3.366866	2.193548	0.656970
6	4.065807	1.226052	0.016474
6	0.699243	1.933079	-3.460239
6	0.017563	0.910899	-4.036349
6	3.178599	-3.547701	0.736807
6	3.174888	-3.617620	-0.617961
6	-3.683662	3.613397	-0.796452
6	-3.900630	4.369377	0.367731
1	-3.820815	3.885101	1.339289
6	-4.196118	5.728636	0.285398
1	-4.352525	6.301164	1.201601
6	-4.281610	6.358634	-0.957682
1	-4.512944	7.423828	-1.018892
6	-4.064926	5.619304	-2.121332
1	-4.130400	6.101810	-3.098439
6	-3.762568	4.260618	-2.042166
1	-3.597226	3.686761	-2.954875
6	-5.360387	1.435190	0.610461
6	-6.425522	2.039352	-0.078698
1	-6.281006	2.364378	-1.108951
6	-7.661454	2.217798	0.540122

Table S4, Continued. Final Atomic Coordinates for Optimized C_2 $[Pd_6(C_2S_2Ph_2)_6]$.

Atomic Numbers	Coordinates (Angstroms) X	Y	Z
1	-8.477899	2.686152	-0.013145
6	-7.858280	1.794460	1.855461
1	-8.827367	1.934393	2.338373
6	-6.809772	1.185849	2.547389
1	-6.953228	0.849072	3.575867
6	-5.573110	1.001647	1.930797
1	-4.758767	0.524679	2.477531
6	-4.345059	-3.892806	1.562846
6	-5.614232	-3.339885	1.317266
1	-5.746702	-2.657104	0.479718
6	-6.694359	-3.649420	2.141294
1	-7.670200	-3.204665	1.936923
6	-6.530626	-4.514558	3.224981
1	-7.378742	-4.755392	3.869077
6	-5.274764	-5.065486	3.482542
1	-5.135149	-5.742681	4.327398
6	-4.190058	-4.753190	2.664346
1	-3.211428	-5.186346	2.873395
6	-4.270108	-4.138432	-1.501899
6	-4.783686	-3.393648	-2.577527
1	-4.388872	-2.396709	-2.777323
6	-5.797204	-3.913644	-3.380681
1	-6.189280	-3.316442	-4.206267
6	-6.307680	-5.189116	-3.133560
1	-7.098453	-5.596825	-3.766339
6	-5.796074	-5.942164	-2.075803
1	-6.181324	-6.944778	-1.879727
6	-4.786044	-5.423920	-1.267360
1	-4.386094	-6.019847	-0.446950
6	-0.751756	3.432882	3.886929
6	-1.996168	4.072754	4.025930
1	-2.909472	3.522933	3.794474
6	-2.074737	5.390665	4.473745
1	-3.051513	5.865374	4.586162
6	-0.911197	6.098582	4.777999
1	-0.971259	7.132645	5.122879
6	0.330275	5.476072	4.636938
1	1.246213	6.023990	4.866532
6	0.411748	4.156094	4.197378
1	1.382562	3.676832	4.084495
6	0.620940	1.101969	5.358992
6	-0.077652	1.605373	6.469068
1	-1.105753	1.945616	6.345795
6	0.529832	1.666392	7.721919
1	-0.030728	2.056698	8.573642

Table S4, Continued. Final Atomic Coordinates for Optimized C_2 [$Pd_6(C_2S_2Ph_2)_6$].

Atomic Numbers	Coordinates (Angstroms) X	Y	Z
6	1.843066	1.225060	7.889849
1	2.317220	1.272406	8.872105
6	2.544095	0.715896	6.795330
1	3.570901	0.365409	6.916353
6	1.938681	0.647974	5.541709
1	2.491046	0.245641	4.691398
6	3.715728	3.640303	0.673946
6	3.842566	4.324785	1.895033
1	3.702096	3.781221	2.830256
6	4.162588	5.681397	1.921178
1	4.267501	6.193370	2.879767
6	4.348241	6.381309	0.728085
1	4.593676	7.444947	0.748188
6	4.215365	5.713865	-0.491021
1	4.349745	6.254222	-1.429979
6	3.903206	4.355750	-0.520868
1	3.790038	3.841835	-1.474222
6	5.368901	1.429387	-0.671667
6	6.454224	1.996388	0.016634
1	6.327751	2.302338	1.055223
6	7.685560	2.164229	-0.614473
1	8.518443	2.604088	-0.062346
6	7.855996	1.768089	-1.941746
1	8.820881	1.900468	-2.434998
6	6.785626	1.199179	-2.634047
1	6.908075	0.889816	-3.673880
6	5.553889	1.025093	-2.004939
1	4.718976	0.585778	-2.552350
6	0.880728	3.288595	-4.046664
6	1.373577	3.435927	-5.354474
1	1.596636	2.547332	-5.945329
6	1.582320	4.702512	-5.896856
1	1.967952	4.795817	-6.914039
6	1.308322	5.845153	-5.143182
1	1.473163	6.836797	-5.569145
6	0.827045	5.711091	-3.839763
1	0.609192	6.597404	-3.240797
6	0.619399	4.445627	-3.292234
1	0.241707	4.350394	-2.273478
6	-0.810542	1.006522	-5.266867
6	-0.714546	0.025175	-6.269705
1	-0.012177	-0.799819	-6.148333
6	-1.500590	0.101641	-7.418724
1	-1.404995	-0.666167	-8.188703
6	-2.405120	1.150940	-7.582720

Table S4, Continued. Final Atomic Coordinates for Optimized C_2 $[Pd_6(C_2S_2Ph_2)_6]$.

Atomic Numbers	X	Y	Z
1	-3.024303	1.207607	-8.480083
6	-2.517166	2.126341	-6.589081
1	-3.228438	2.946489	-6.705222
6	-1.729969	2.056571	-5.442348
1	-1.827212	2.814140	-4.666129
6	4.337063	-3.867560	1.614341
6	4.172020	-4.696100	2.738529
1	3.189772	-5.116925	2.955629
6	5.251511	-4.992916	3.569405
1	5.104053	-5.645681	4.431967
6	6.512117	-4.458175	3.301469
1	7.356277	-4.687089	3.955000
6	6.685904	-3.624488	2.194796
1	7.666248	-3.193852	1.981652
6	5.611035	-3.329965	1.358735
1	5.750934	-2.671348	0.503001
6	4.272397	-4.188836	-1.444756
6	4.792204	-3.488121	-2.546343
1	4.406343	-2.495283	-2.780059
6	5.798557	-4.046094	-3.332748
1	6.195411	-3.482536	-4.179467
6	6.295813	-5.317623	-3.042679
1	7.080753	-5.755518	-3.662364
6	5.777530	-6.028215	-1.959071
1	6.151555	-7.027969	-1.729605
6	4.774657	-5.471526	-1.167657
1	4.369173	-6.035084	-0.327423

Table S5. Final Atomic Coordinates for Optimized S_6 $[Pd_6(C_2S_2Ph_2)_6]$. E(RB3LYP)= -8776.48244772 A.U.

Center Number	Atomic Number	Atomic Type	Coordinates (Angstroms) X	Y	Z
1	46	0	1.815967	-0.900265	-1.351221
2	46	0	0.106808	-2.011833	1.345780
3	46	0	-1.695704	-1.130931	-1.367079
4	16	0	1.682316	1.030316	-2.707822
5	16	0	3.425720	0.289691	-0.130304
6	16	0	1.697888	-0.918936	2.711324
7	16	0	1.958433	-2.817277	0.152359
8	16	0	0.065481	-1.958099	-2.702681
9	16	0	-1.466394	-3.122591	-0.146731
10	6	0	3.195750	1.935113	-2.324258
11	6	0	3.914182	1.645867	-1.213318
12	6	0	3.252993	-1.751059	2.340740
13	6	0	3.354564	-2.571493	1.267164
14	6	0	0.136786	-3.715047	-2.311133
15	6	0	-0.535461	-4.210343	-1.244446
16	46	0	-1.815924	0.900313	1.351185
17	46	0	-0.106788	2.011873	-1.345797
18	46	0	1.695735	1.130990	1.367074
19	16	0	-1.682296	-1.030294	2.707768
20	16	0	-3.425686	-0.289633	0.130262
21	16	0	-1.697897	0.919002	-2.711317
22	16	0	-1.958376	2.817363	-0.152335
23	16	0	-0.065431	1.958147	2.702649
24	16	0	1.466411	3.122586	0.146680
25	6	0	-3.195677	-1.935136	2.324147
26	6	0	-3.914126	-1.645853	1.213232
27	6	0	-3.252988	1.751141	-2.340696
28	6	0	-3.354513	2.571583	-1.267122
29	6	0	-0.136692	3.715095	2.311121
30	6	0	0.535472	4.210391	1.244335
31	6	0	-4.353605	1.406608	-3.278745
32	6	0	-4.145910	1.456612	-4.668797
33	6	0	-5.611426	0.997173	-2.802554
34	6	0	-5.170614	1.125196	-5.554025
35	6	0	-6.630274	0.659875	-3.690253
36	6	0	-6.415991	0.724841	-5.068602
37	1	0	-3.174542	1.766179	-5.055610
38	1	0	-5.780659	0.934133	-1.729021
39	1	0	-4.991937	1.178587	-6.629640
40	1	0	-7.597195	0.335414	-3.300983
41	1	0	-7.216232	0.458872	-5.761998
42	6	0	-4.545022	3.393644	-0.920598
43	6	0	-5.116133	4.255849	-1.871779
44	6	0	-5.092553	3.362780	0.373775
45	6	0	-6.211659	5.051610	-1.541724

Table S5, Continued. Final Atomic Coordinates for Optimized S_6 $[Pd_6(C_2S_2Ph_2)_6]$.

Center Number	Atomic Number	Atomic Type	Coordinates (Angstroms) X	Y	Z
46	6	0	-6.190576	4.157982	0.699010
47	6	0	-6.754726	5.004742	-0.256625
48	1	0	-4.692622	4.301037	-2.875262
49	1	0	-4.657028	2.702265	1.124428
50	1	0	-6.640188	5.716952	-2.293915
51	1	0	-6.606959	4.113798	1.707273
52	1	0	-7.612731	5.629115	0.000170
53	6	0	0.648143	5.652200	0.896466
54	6	0	1.116559	6.575564	1.846102
55	6	0	0.334949	6.113953	-0.393561
56	6	0	1.256100	7.922825	1.517768
57	6	0	0.472670	7.462972	-0.717055
58	6	0	0.933917	8.372349	0.236243
59	1	0	1.375574	6.228394	2.846475
60	1	0	-0.033165	5.408940	-1.139873
61	1	0	1.624299	8.624895	2.268512
62	1	0	0.214573	7.805333	-1.721131
63	1	0	1.043848	9.427974	-0.019562
64	6	0	-1.008629	4.496937	3.227051
65	6	0	-2.001206	5.357565	2.725537
66	6	0	-0.876107	4.363463	4.620818
67	6	0	-2.826725	6.068631	3.593801
68	6	0	-1.699940	5.082001	5.485923
69	6	0	-2.678755	5.935918	4.976140
70	1	0	-0.114532	3.696506	5.026078
71	1	0	-3.596567	6.725972	3.185122
72	1	0	-1.575895	4.971119	6.564840
73	1	0	-3.327649	6.493962	5.654091
74	6	0	3.567852	2.945484	-3.350561
75	6	0	4.851746	2.937672	-3.921460
76	6	0	2.635388	3.892080	-3.808763
77	6	0	5.196369	3.859248	-4.908292
78	6	0	2.985180	4.814779	-4.793120
79	6	0	4.266401	4.802985	-5.346733
80	1	0	5.580705	2.198372	-3.590105
81	1	0	1.631042	3.905425	-3.383621
82	1	0	6.198162	3.834278	-5.341713
83	1	0	2.249059	5.547188	-5.130240
84	1	0	4.537472	5.523449	-6.120872
85	6	0	5.124545	2.375613	-0.748018
86	6	0	6.302197	1.673105	-0.436757
87	6	0	5.113039	3.771964	-0.587379
88	6	0	7.438914	2.348497	0.005524
89	6	0	6.248970	4.442448	-0.138091
90	6	0	7.415845	3.735361	0.158491

Table S5, Continued. Final Atomic Coordinates for Optimized S_6 $[Pd_6(C_2S_2Ph_2)_6]$.

Center Number	Atomic Number	Atomic Type	Coordinates (Angstroms) X	Y	Z
91	1	0	6.327680	0.589282	-0.557717
92	1	0	4.202271	4.326979	-0.807032
93	1	0	8.347265	1.786418	0.231469
94	1	0	6.219111	5.526652	-0.014100
95	1	0	8.303962	4.263870	0.510468
96	6	0	4.353543	-1.406590	3.278891
97	6	0	4.145745	-1.456701	4.668923
98	6	0	5.611396	-0.997122	2.802818
99	6	0	5.170385	-1.125351	5.554249
100	6	0	6.630181	-0.659890	3.690617
101	6	0	6.415797	-0.724960	5.068946
102	1	0	3.174346	-1.766292	5.055639
103	1	0	5.780701	-0.934006	1.729302
104	1	0	4.991631	-1.178821	6.629848
105	1	0	7.597131	-0.335401	3.301442
106	1	0	7.215988	-0.459044	5.762420
107	6	0	4.545067	-3.393582	0.920694
108	6	0	5.116295	-4.255598	1.871978
109	6	0	5.092485	-3.362939	-0.373735
110	6	0	6.211820	-5.051380	1.541968
111	6	0	6.190505	-4.158163	-0.698925
112	6	0	6.754772	-5.004728	0.256814
113	1	0	4.692879	-4.300627	2.875508
114	1	0	4.656871	-2.702582	-1.124474
115	1	0	6.640438	-5.716569	2.294244
116	1	0	6.606789	-4.114149	-1.707235
117	1	0	7.612776	-5.629119	0.000057
118	6	0	1.008873	-4.496935	-3.226850
119	6	0	2.001443	-5.357332	-2.724949
120	6	0	0.876642	-4.363702	-4.620658
121	6	0	2.827260	-6.068460	-3.592888
122	6	0	1.700792	-5.082262	-5.485439
123	6	0	2.679600	-5.935973	-4.975282
124	1	0	0.115061	-3.696922	-5.026202
125	1	0	3.597028	-6.725703	-3.183918
126	1	0	1.577015	-4.971553	-6.564406
127	6	0	-0.648506	-5.652185	-0.896725
128	6	0	-1.119155	-6.574927	-1.845831
129	6	0	-0.333576	-6.114444	0.392684
130	6	0	-1.259506	-7.922114	-1.517464
131	6	0	-0.472252	-7.463338	0.716263
132	6	0	-0.935935	-8.372101	-0.236454
133	1	0	-1.379276	-6.227325	-2.845775
134	1	0	-1.629455	-8.623744	-2.267759
135	1	0	-0.212829	-7.806109	1.719857

Table S5, Continued. Final Atomic Coordinates for Optimized S_6 $[Pd_6(C_2S_2Ph_2)_6]$.

Center Number	Atomic Number	Atomic Type	Coordinates (Angstroms) X	Y	Z
136	1	0	-1.046588	-9.427643	0.019383
137	6	0	-3.567673	-2.945665	3.350337
138	6	0	-4.851464	-2.937862	3.921462
139	6	0	-2.635190	-3.892405	3.808199
140	6	0	-5.195976	-3.859592	4.908191
141	6	0	-2.984871	-4.815255	4.792454
142	6	0	-4.265994	-4.803470	5.346297
143	1	0	-5.580432	-2.198455	3.590363
144	1	0	-1.630924	-3.905743	3.382867
145	1	0	-6.197691	-3.834634	5.341793
146	1	0	-2.248744	-5.547780	5.129307
147	1	0	-4.536977	-5.524054	6.120355
148	6	0	-5.124447	-2.375639	0.747882
149	6	0	-6.302150	-1.673175	0.436717
150	6	0	-5.112845	-3.771971	0.587096
151	6	0	-7.438834	-2.348599	-0.005600
152	6	0	-6.248744	-4.442485	0.137771
153	6	0	-7.415675	-3.735445	-0.158704
154	1	0	-6.327695	-0.589365	0.557779
155	1	0	-4.202031	-4.326944	0.806668
156	1	0	-8.347227	-1.786558	-0.231467
157	1	0	-6.218818	-5.526674	0.013670
158	1	0	-8.303766	-4.263981	-0.510710
159	1	0	2.130099	-5.454627	-1.648177
160	1	0	0.036697	-5.409843	1.138325
161	1	0	-2.130066	5.455127	1.648819
162	1	0	3.328726	-6.494029	-5.653001

Table S6. Final Atomic Coordinates for Optimized C_2 [$Pt_6(C_2S_2Ph_2)_6$].

Atomic Number	Coordinates (Angstroms) X	Y	Z
78	0.000920	-2.959820	-0.030101
78	-1.761804	-0.548946	1.711312
78	-1.830562	-0.469650	-1.664064
78	1.752414	-0.489580	-1.729850
78	1.824268	-0.535100	1.650868
78	-0.005726	1.981629	0.031026
16	1.725148	-2.913540	-1.635691
16	1.723089	-2.960064	1.582457
16	-1.733075	-2.975822	1.568322
16	-1.713981	-2.891389	-1.647778
16	0.009236	-0.650162	3.260843
16	-1.548192	1.782102	1.887590
16	-3.473402	-0.404440	0.012561
16	-1.846941	1.877675	-1.528330
16	-0.020968	-0.543850	-3.282545
16	1.538659	1.844139	-1.832045
16	1.828451	1.815603	1.588921
16	3.462830	-0.401463	-0.021278
6	3.345423	2.255141	0.712947
6	4.049190	1.311509	0.049769
6	3.188900	-3.451394	-0.713355
6	3.175552	-3.502932	0.638125
6	0.656992	2.129650	-3.386583
6	0.025905	1.114830	-4.017822
6	-3.362139	2.276635	-0.628622
6	-4.058886	1.308715	0.004860
6	-0.673636	2.012081	3.457469
6	-0.002554	0.988718	4.034108
6	-3.181880	-3.448416	-0.742406
6	-3.174704	-3.518407	0.608762
6	3.679145	3.702593	0.791032
6	3.906480	4.457903	-0.371394
1	3.837900	3.972916	-1.343334
6	4.200832	5.817182	-0.287135
1	4.364932	6.389380	-1.202187
6	4.276045	6.447395	0.956355
1	4.506500	7.512668	1.019274
6	4.049990	5.708272	2.118293
1	4.107228	6.190961	3.095798
6	3.747524	4.349681	2.037218
1	3.574119	3.776811	2.948956
6	5.356100	1.523349	-0.625873
6	6.423311	2.128007	0.059637
1	6.281491	2.455753	1.089363
6	7.657676	2.303581	-0.562752

Table S6, Continued. Final Atomic Coordinates for Optimized C_2 $[Pt_6(C_2S_2Ph_2)_6]$.

Atomic Number	X	Y	Z
1	8.476161	2.772130	-0.012686
6	7.850465	1.877105	-1.877843
1	8.818504	2.014903	-2.363482
6	6.799789	1.267673	-2.565490
1	6.940367	0.927647	-3.593250
6	5.564255	1.085583	-1.945656
1	4.748275	0.606426	-2.488682
6	4.352281	-3.775687	-1.582227
6	5.618998	-3.218813	-1.334002
1	5.749344	-2.541481	-0.491685
6	6.699032	-3.517185	-2.162211
1	7.673058	-3.069125	-1.956588
6	6.537245	-4.375482	-3.251516
1	7.385277	-4.607731	-3.898835
6	5.283523	-4.930468	-3.511065
1	5.145680	-5.602318	-4.360430
6	4.198534	-4.628735	-2.689334
1	3.221540	-5.064649	-2.900321
6	4.272751	-4.033167	1.490171
6	4.768044	-3.291991	2.577002
1	4.360032	-2.301277	2.782679
6	5.780047	-3.809155	3.384114
1	6.158043	-3.215568	4.218761
6	6.305818	-5.076982	3.130384
1	7.094897	-5.482214	3.766870
6	5.811267	-5.826337	2.061802
1	6.208137	-6.823452	1.860998
6	4.803393	-5.311445	1.248839
1	4.416239	-5.904135	0.420085
6	0.715369	3.540966	-3.855218
6	1.954397	4.190531	-3.994968
1	2.873056	3.646540	-3.771194
6	2.020238	5.511541	-4.435539
1	2.992590	5.994808	-4.549629
6	0.849772	6.212092	-4.730496
1	0.900635	7.248565	-5.069549
6	-0.386301	5.579796	-4.587036
1	-1.307538	6.122161	-4.808176
6	-0.455182	4.256550	-4.155211
1	-1.421020	3.768142	-4.039697
6	-0.640371	1.202594	-5.343713
6	0.047443	1.724174	-6.452165
1	1.070521	2.079332	-6.329947
6	-0.564195	1.782610	-7.702957
1	-0.012108	2.186994	-8.553654

Table S6, Continued. Final Atomic Coordinates for Optimized C_2 [$Pt_6(C_2S_2Ph_2)_6$].

Atomic Number	Coordinates (Angstroms) X	Y	Z
6	-1.870573	1.320891	-7.870472
1	-2.347696	1.366597	-8.851372
6	-2.560208	0.793415	-6.777573
1	-3.581146	0.426176	-6.898119
6	-1.950987	0.727443	-5.525504
1	-2.494491	0.309812	-4.676399
6	-3.711990	3.722647	-0.646871
6	-3.837407	4.408373	-1.867184
1	-3.691908	3.867710	-2.803279
6	-4.163913	5.763453	-1.891485
1	-4.268047	6.276927	-2.849350
6	-4.356863	6.460054	-0.697635
1	-4.606670	7.522684	-0.716595
6	-4.225814	5.790910	0.520499
1	-4.366884	6.328635	1.459939
6	-3.907673	4.434149	0.548601
1	-3.799230	3.918067	1.501236
6	-5.361592	1.501562	0.695369
6	-6.450148	2.068124	0.011990
1	-6.327367	2.379524	-1.025367
6	-7.680299	2.228606	0.646997
1	-8.516101	2.668225	0.099104
6	-7.845941	1.825613	1.972940
1	-8.810102	1.952351	2.469090
6	-6.772148	1.256772	2.659689
1	-6.891141	0.941110	3.697997
6	-5.541101	1.089528	2.027049
1	-4.703475	0.648219	2.569243
6	-0.850928	3.370026	4.037323
6	-1.351991	3.523766	5.341284
1	-1.586524	2.638196	5.932239
6	-1.553777	4.793370	5.879029
1	-1.946488	4.892571	6.892940
6	-1.263315	5.931792	5.125010
1	-1.422623	6.925742	5.547728
6	-0.773113	5.790811	3.825766
1	-0.542725	6.673774	3.226644
6	-0.572842	4.522498	3.281786
1	-0.188818	4.421681	2.265783
6	0.821591	1.071026	5.267457
6	0.721415	0.081854	6.261792
1	0.019963	-0.742510	6.131493
6	1.502898	0.149783	7.414421
1	1.404262	-0.623956	8.178018
6	2.406947	1.197701	7.590028

Table S6, Continued. Final Atomic Coordinates for Optimized C_2 $[Pt_6(C_2S_2Ph_2)_6]$.

Atomic Number	Coordinates (Angstroms) X	Y	Z
1	3.022701	1.247144	8.490157
6	2.523262	2.180318	6.604427
1	3.234398	2.999252	6.729325
6	1.740182	2.119574	5.454306
1	1.838593	2.885079	4.686139
6	-4.339729	-3.759029	-1.622650
6	-4.177353	-4.578688	-2.753481
1	-3.196878	-5.001453	-2.974631
6	-5.258281	-4.864203	-3.586422
1	-5.113551	-5.510271	-4.454433
6	-6.516566	-4.326182	-3.314035
1	-7.361483	-4.545724	-3.969767
6	-6.687076	-3.500957	-2.200636
1	-7.665487	-3.067615	-1.984264
6	-5.611240	-3.218418	-1.361778
1	-5.748368	-2.566977	-0.500142
6	-4.266616	-4.080710	1.446572
6	-4.757349	-3.382137	2.563035
1	-4.350445	-2.398696	2.801889
6	-5.761461	-3.931688	3.358399
1	-6.135525	-3.370529	4.216950
6	-6.284393	-5.191567	3.063197
1	-7.067191	-5.622615	3.690345
6	-5.793871	-5.900123	1.965298
1	-6.187367	-6.891496	1.732140
6	-4.793676	-5.352438	1.164598
1	-4.409087	-5.914707	0.313867

Table S7. Final Atomic Coordinates for Optimized C_2 [$Pd_6(C_2S_2(COOH)_2)_6$] E(RB3LYP) = -8266.69688075.

Center Number	Atomic Number	Coordinates (Angstroms) X	Y	Z
1	46	-0.052193	2.806852	0.008910
2	46	0.010562	-2.089132	-0.002454
3	46	1.702504	0.415741	1.726338
4	46	-1.839744	0.359513	1.608637
5	46	1.805031	0.394387	-1.637965
6	46	-1.714189	0.363683	-1.764665
7	16	-1.855460	-1.997991	1.569921
8	16	-3.460244	0.227567	-0.099191
9	16	-1.742901	2.807480	-1.647582
10	16	-1.791316	2.809071	1.614549
11	16	-1.504487	-1.977589	-1.906481
12	16	0.038832	0.481210	-3.345271
13	16	3.455508	0.329174	0.031515
14	16	1.869201	-1.965254	-1.556668
15	16	1.574098	-1.929290	1.880938
16	16	-0.070416	0.462913	3.294107
17	16	1.684768	2.829150	-1.601421
18	16	1.643256	2.867970	1.663673
19	6	-3.318556	-2.408348	0.651508
20	6	-3.985051	-1.484041	-0.070801
21	6	-3.204470	3.248754	-0.711400
22	6	-3.206660	3.272027	0.639342
23	6	-0.524441	-2.214336	-3.385265
24	6	0.081553	-1.176130	-4.003381
25	6	3.332543	-2.339115	-0.611826
26	6	4.021494	-1.366995	0.023499
27	6	0.653690	-2.185000	3.380467
28	6	-0.056986	-1.197349	3.966316
29	6	3.103607	3.330963	-0.629708
30	6	3.068111	3.365167	0.720775
31	6	-3.763462	-3.841600	0.843936
32	8	-3.246859	-4.656682	-0.086686
33	8	-4.429858	-4.188757	1.788115
34	6	-5.182774	-1.873223	-0.871401
35	8	-5.684314	-2.977069	-0.849417
36	1	-6.432445	-1.224704	-2.112648
37	8	-5.652456	-0.862577	-1.633466
38	6	-4.461834	3.535073	-1.466616
39	8	-5.531386	3.773577	-0.945551
40	1	-5.173516	3.669069	-3.196748
41	8	-4.288543	3.491497	-2.803974
42	6	-4.407331	3.609640	1.492144
43	8	-5.039785	2.785865	2.109159
44	1	-5.394200	5.070235	2.134436
45	8	-4.599814	4.938010	1.565827

Table S7, Continued. Final Atomic Coordinates for Optimized C_2 $[Pd_6(C_2S_2(COOH)_2)_6]$

Center Number	Atomic Number	Coordinates (Angstroms) X	Y	Z
46	6	-0.396254	-3.653917	-3.770697
47	8	-0.980377	-4.551961	-3.205650
48	8	0.465364	-3.856552	-4.789151
49	6	0.932689	-1.254571	-5.247089
50	8	0.183607	-1.439812	-6.348859
51	1	0.811396	-1.456528	-7.109129
52	8	2.128253	-1.081330	-5.250812
53	6	3.667160	-3.801534	-0.603563
54	8	3.372426	-4.558283	-1.501666
55	1	4.485067	-5.148218	0.418191
56	8	4.277792	-4.191866	0.532548
57	6	5.350271	-1.577799	0.698746
58	8	6.295700	-2.118745	0.175954
59	1	6.287059	-1.127739	2.271050
60	8	5.368442	-1.030717	1.928616
61	6	0.713030	-3.615971	3.856437
62	8	1.459613	-3.738704	4.967087
63	1	1.431686	-4.692555	5.214539
64	8	0.232018	-4.540342	3.243992
65	6	-0.930968	-1.485735	5.146442
66	8	-1.503036	-0.373944	5.649565
67	1	-2.087265	-0.673166	6.383533
68	8	-1.120767	-2.596167	5.594582
69	6	4.372114	3.649188	-1.354220
70	8	4.230625	3.607134	-2.694224
71	1	5.120836	3.797518	-3.069078
72	8	5.423376	3.906559	-0.805209
73	6	4.232483	3.761011	1.598819
74	8	4.875923	2.974194	2.251801
75	1	5.150032	5.269704	2.233802
76	8	4.377107	5.096641	1.646934
77	1	-3.555818	-5.567729	0.128299
78	1	0.508861	-4.831181	-4.921431

Table S8. Final Atomic Coordinates for Optimized S_6 $[Pd_6(C_2S_2(COOH)_2)_6]$ E(RB3LYP) = -8266.69946847.

Center Number	Atomic Number	Coordinates (Angstroms) X	Y	Z
1	46	1.394894	1.442201	1.400889
2	46	-0.570375	1.961554	-1.338305
3	46	-1.991641	0.431567	1.348805
4	16	1.906024	-0.451545	2.730046
5	16	3.336070	0.945115	0.174897
6	16	1.305946	1.504263	-2.705298
7	16	0.867063	3.314344	-0.056710
8	16	-0.631513	1.790249	2.724049
9	16	-2.465030	2.382026	0.134471
10	8	5.513156	-2.113495	2.893715
11	8	3.587050	-2.397288	4.031710
12	8	5.889747	-1.204452	-0.143302
13	8	6.495099	0.301468	1.434420
14	8	4.534360	2.074296	-3.186983
15	8	3.495158	3.892821	-4.045114
16	8	4.342764	4.738689	-1.355028
17	8	3.026138	5.156927	0.426598
18	8	-0.764805	5.752606	2.838583
19	8	0.046054	4.160308	4.213810
20	8	-2.119138	5.567002	-0.233209
21	8	-3.387515	5.512114	1.639662
22	6	3.607244	-0.797141	2.287296
23	6	4.198472	-0.197935	1.231972
24	6	4.350820	-1.820702	3.082226
25	6	5.613489	-0.466826	0.771428
26	1	4.155853	-3.070687	4.470094
27	1	7.383232	0.082123	1.066581
28	6	2.449205	2.786636	-2.241239
29	6	2.288077	3.529095	-1.125380
30	6	3.634126	2.879548	-3.174202
31	6	3.327945	4.523338	-0.725933
32	1	4.301837	3.883503	-4.611879
33	1	3.774079	5.772364	0.602488
34	6	-1.159704	3.449289	2.305121
35	6	-1.951569	3.682048	1.237379
36	6	-0.626979	4.581993	3.123081
37	6	-2.452984	5.036903	0.800771
38	1	0.377775	4.972882	4.659672
39	1	-3.659272	6.390489	1.284175
40	46	-1.394893	-1.442201	-1.400889
41	46	0.570375	-1.961555	1.338305
42	46	1.991642	-0.431567	-1.348806
43	16	-1.906024	0.451545	-2.730046
44	16	-3.336069	-0.945115	-0.174897
45	16	-1.305945	-1.504263	2.705298

Table S8, Continued. Final Atomic Coordinates for Optimized S_6 $[Pd_6(C_2S_2(COOH)_2)_6]$.

Center Number	Atomic Number	Coordinates (Angstroms) X	Y	Z
46	16	-0.867063	-3.314344	0.056710
47	16	0.631514	-1.790249	-2.724049
48	16	2.465030	-2.382026	-0.134471
49	8	-5.513156	2.113497	-2.893714
50	8	-3.587051	2.397286	-4.031711
51	8	-5.889746	1.204451	0.143304
52	8	-6.495100	-0.301466	-1.434421
53	8	-4.534359	-2.074293	3.186982
54	8	-3.495160	-3.892820	4.045113
55	8	-4.342765	-4.738687	1.355027
56	8	-3.026138	-5.156927	-0.426597
57	8	0.764802	-5.752607	-2.838581
58	8	-0.046053	-4.160308	-4.213811
59	8	2.119138	-5.567000	0.233212
60	8	3.387513	-5.512116	-1.639661
61	6	-3.607244	0.797141	-2.287296
62	6	-4.198472	0.197935	-1.231972
63	6	-4.350820	1.820702	-3.082226
64	6	-5.613489	0.466826	-0.771428
65	1	-4.155853	3.070686	-4.470095
66	1	-7.383232	-0.082120	-1.066582
67	6	-2.449205	-2.786636	2.241239
68	6	-2.288077	-3.529095	1.125381
69	6	-3.634127	-2.879546	3.174201
70	6	-3.327945	-4.523338	0.725933
71	1	-4.301840	-3.883501	4.611878
72	1	-3.774080	-5.772363	-0.602488
73	6	1.159705	-3.449290	-2.305120
74	6	1.951569	-3.682049	-1.237378
75	6	0.626979	-4.581993	-3.123081
76	6	2.452983	-5.036903	-0.800769
77	1	-0.377775	-4.972882	-4.659672
78	1	3.659269	-6.390491	-1.284173

Table S9. Calculated Electronic Transitions for $[Ni_6(S_2C_2Ph_2)_6]$.

Index	Energy (cm^{-1})	Wavelength	Oscillator Strength	Contributions
0	10356.159	965.609	0.000e+00	32.4% H-7 => LUMO,<br>15.6% H-7 => L+2,<br>7.83% H-3 => LUMO,<br>7.28% H-9 => LUMO,<br>6.16% H-2 => LUMO,<br>3.99% H-9 => L+2,<br>3.20% H-10 => LUMO,<br>2.30% H-3 => L+2
1	11757.950	850.488	6.000e-04	17.6% HOMO => LUMO,<br>16.9% H-8 => LUMO,<br>6.24% H-12 => LUMO,<br>4.98% H-13 => LUMO,<br>3.85% H-8 => L+2,<br>2.89% HOMO => L+2,<br>2.73% H-33 => LUMO,<br>2.27% H-33 => L+2,<br>2.18% H-13 => L+2
2	11895.065	840.685	1.000e-04	30.6% H-6 => LUMO,<br>7.95% H-11 => LUMO,<br>5.24% H-8 => L+3,<br>4.47% H-10 => L+1,<br>4.18% H-5 => L+1,<br>2.98% H-2 => LUMO,<br>2.95% H-11 => L+2,<br>2.61% H-9 => L+1
3	12328.184	811.149	1.800e-03	19.9% H-6 => L+1,<br>11.6% H-5 => LUMO,<br>9.58% H-10 => LUMO,<br>6.71% H-8 => L+5,<br>5.20% H-9 => LUMO,<br>3.44% H-41 => L+3,<br>2.62% H-12 => LUMO,<br>2.49% H-9 => L+3,<br>2.20% H-9 => L+2

Table S9, Continued. Calculated Electronic Transitions for $[Ni_6(S_2C_2Ph_2)_6]$.

Index	Energy (cm^{-1})	Wavelength	Oscillator Strength	Contributions
4	12538.695	797.531	2.000e-04	9.29% H-11 => LUMO,<br>7.99% H-6 => L+2,<br>5.14% H-10 => L+1,<br>4.43% H-8 => L+3,<br>3.62% H-8 => LUMO,<br>3.56% H-11 => L+2,<br>3.35% H-6 => L+3,<br>3.34% H-15 => LUMO,<br>2.64% H-5 => L+1,<br>2.55% H-10 => L+5,<br>2.19% H-6 => L+4,<br>2.13% H-8 => L+1
5	12574.990	795.229	5.000e-04	10.6% H-6 => L+3,<br>7.42% H-8 => LUMO,<br>6.21% H-9 => L+5,<br>5.69% H-8 => L+2,<br>5.33% H-9 => L+1,<br>3.68% H-11 => LUMO,<br>3.65% HOMO => LUMO,<br>3.61% H-6 => L+2,<br>3.14% H-5 => L+5,<br>2.23% H-41 => L+1
6	12682.262	788.503	1.000e-04	11.4% H-8 => L+1,<br>9.01% H-6 => L+5,<br>6.15% H-5 => L+3,<br>5.68% H-10 => L+3,<br>4.38% H-3 => LUMO,<br>3.83% H-9 => L+3,<br>2.95% H-10 => L+1,<br>2.87% H-2 => LUMO,<br>2.32% H-3 => L+1,<br>2.24% H-11 => LUMO,<br>2.23% H-41 => LUMO
7	13307.342	751.465	2.000e-04	12.1% H-2 => LUMO,<br>5.89% H-10 => L+3,<br>4.28% H-9 => LUMO,<br>4.05% H-8 => L+1,<br>3.26% H-2 => L+1,<br>3.01% H-9 => L+3,<br>2.50% H-13 => LUMO,<br>2.46% H-8 => L+5,<br>2.37% H-6 => L+5,<br>2.18% H-8 => LUMO,<br>2.04% H-16 => LUMO

Table S9, Continued. Calculated Electronic Transitions for $[Ni_6(S_2C_2Ph_2)_6]$.

Index	Energy (cm^{-1})	Wavelength	Oscillator Strength	Contributions
8	13413.000	745.545	5.000e-04	7.97% H-3 => LUMO,<br>5.35% H-1 => L+3,<br>3.84% H-14 => L+3,<br>3.63% H-3 => L+1,<br>3.50% H-16 => LUMO,<br>3.40% H-9 => LUMO,<br>3.23% H-1 => LUMO,<br>3.16% HOMO => LUMO,<br>2.86% H-9 => L+3,<br>2.44% HOMO => L+2,<br>2.28% H-7 => L+1,<br>2.12% H-9 => L+1
9	13483.170	741.665	1.300e-03	16.1% H-1 => LUMO,<br>3.99% H-16 => L+3,<br>3.49% H-14 => LUMO,<br>2.83% H-11 => L+2,<br>2.66% H-11 => LUMO,<br>2.22% H-2 => L+3,<br>2.18% H-3 => L+3,<br>2.16% H-7 => L+5,<br>2.13% HOMO => L+3
10	13838.861	722.603	3.000e-04	5.20% H-14 => LUMO,<br>5.20% H-2 => L+3,<br>5.15% H-3 => L+3,<br>4.77% H-1 => L+1,<br>2.66% H-10 => LUMO,<br>2.63% H-13 => L+3,<br>2.22% H-6 => L+5
11	14018.723	713.332	6.000e-04	6.28% H-14 => L+1,<br>6.20% H-8 => L+3,<br>4.93% H-7 => L+1,<br>4.21% H-3 => L+1,<br>3.44% H-14 => L+5,<br>2.86% H-26 => LUMO,<br>2.78% H-6 => LUMO,<br>2.62% H-2 => L+1,<br>2.61% H-13 => L+1,<br>2.29% H-6 => L+3

Table S9, Continued. Calculated Electronic Transitions for [$Ni_6(S_2C_2Ph_2)_6$].

Index	Energy (cm^{-1})	Wavelength	Oscillator Strength	Contributions
12	14059.050	711.286	4.000e-04	7.78% H-7 => LUMO,<br>4.69% HOMO => L+1,<br>4.12% H-7 => L+2,<br>3.84% H-12 => L+1,<br>3.77% H-16 => L+1,<br>2.74% H-11 => L+5,<br>2.02% H-8 => L+5
13	14102.604	709.089	3.000e-04	17.0% H-9 => L+4,<br>14.4% H-7 => L+4,<br>11.0% H-10 => L+4,<br>8.47% H-4 => L+4,<br>5.62% H-8 => L+4,<br>4.77% H-11 => L+4,<br>2.85% H-42 => L+4,<br>2.60% H-7 => L+2
14	14276.820	700.436	1.000e-04	6.16% H-7 => L+3,<br>4.04% H-1 => L+1,<br>3.80% H-11 => L+1,<br>2.83% H-12 => L+5,<br>2.61% H-14 => L+1,<br>2.22% H-6 => L+5,<br>2.11% H-13 => L+3
15	14304.243	699.093	1.000e-04	7.73% HOMO => L+4,<br>3.39% H-7 => L+3,<br>3.31% H-13 => L+5,<br>3.29% H-6 => L+3,<br>3.19% H-8 => LUMO,<br>2.96% H-10 => L+1,<br>2.30% H-2 => L+5,<br>2.01% H-13 => L+4
16	14477.652	690.720	3.000e-04	26.5% HOMO => L+4,<br>4.13% H-12 => L+4,<br>3.63% HOMO => L+2,<br>3.10% H-8 => L+4,<br>2.81% H-49 => L+4,<br>2.73% H-13 => L+5,<br>2.20% H-1 => L+4

Table S9, Continued. Calculated Electronic Transitions for $[Ni_6(S_2C_2Ph_2)_6]$.

Index	Energy (cm^{-1})	Wavelength	Oscillator Strength	Contributions
17	15805.241	632.702	4.000e-04	20.8% H-11 => L+4,<br>6.59% H-6 => L+4,<br>6.46% H-22 => L+4,<br>5.56% H-46 => L+4,<br>4.81% H-15 => L+4,<br>3.53% H-16 => L+4,<br>2.64% H-26 => L+4,<br>2.43% H-11 => L+2,<br>2.40% H-35 => L+4,<br>2.29% H-1 => L+4,<br>2.22% H-27 => L+4
18	17215.098	580.885	1.750e-02	47.2% HOMO => LUMO,<br>7.45% H-2 => LUMO,<br>3.93% H-1 => LUMO,<br>3.34% HOMO => L+4,<br>2.80% H-8 => L+2
19	17318.337	577.423	1.100e-03	13.4% H-58 => LUMO,<br>10.6% H-58 => L+2,<br>6.96% H-52 => LUMO,<br>5.10% H-45 => LUMO,<br>5.07% H-52 => L+2,<br>3.54% H-45 => L+2,<br>2.98% H-57 => LUMO,<br>2.91% H-35 => LUMO,<br>2.83% HOMO => LUMO,<br>2.51% H-57 => L+2

Table S10. Calculated Electronic Transitions for $[Pd_6(S_2C_2Ph_2)_6]$.

Index	Energy (cm^{-1})	Wavelength	Oscillator Strength	Contributions
0	13971.136	715.761	1.300e-03	45.1% H-2 => LUMO, 23.7% HOMO => LUMO, 12.6% H-1 => LUMO, 7.63% H-3 => LUMO, 2.28% H-7 => LUMO
1	14209.069	703.776	1.500e-02	64.9% HOMO => LUMO, 17.5% H-1 => LUMO, 6.78% H-2 => LUMO
2	14490.557	690.105	5.600e-03	59.4% H-1 => LUMO, 25.1% H-2 => LUMO, 5.34% HOMO => LUMO, 2.11% H-3 => LUMO
3	14762.366	677.398	9.700e-03	70.8% H-3 => LUMO, 11.6% H-2 => LUMO, 5.17% H-6 => LUMO
4	16683.578	599.392	6.300e-03	57.2% H-4 => LUMO, 9.59% H-5 => LUMO, 7.68% H-6 => LUMO, 7.22% H-8 => LUMO, 3.66% H-1 => LUMO
5	16742.457	597.284	8.400e-03	58.0% H-5 => LUMO, 6.93% H-4 => LUMO, 5.88% H-2 => L+1, 4.99% HOMO => L+1, 2.73% H-14 => LUMO, 2.13% H-1 => L+1
6	17164.285	582.605	4.300e-03	42.4% H-6 => LUMO, 20.7% H-8 => LUMO, 4.32% H-3 => L+1, 3.34% H-3 => LUMO, 3.23% H-1 => L+3
7	17261.071	579.338	8.000e-04	19.8% H-7 => LUMO, 16.9% H-8 => LUMO, 13.6% H-6 => LUMO, 10.3% H-4 => LUMO, 5.13% HOMO => L+1, 3.54% H-11 => LUMO, 3.02% H-12 => LUMO, 2.79% H-10 => LUMO

Table S10, Continued. Calculated Electronic Transitions for $[Pd_6(S_2C_2Ph_2)_6]$.

Index	Energy (cm^{-1})	Wavelength	Oscillator Strength	Contributions
8	17432.061	573.656	5.300e-03	33.0% H-7 => LUMO,<br>20.2% H-8 => LUMO,<br>4.72% HOMO => L+2,<br>3.84% H-6 => LUMO,<br>3.80% H-4 => LUMO,<br>3.17% H-1 => L+1,<br>2.62% H-14 => LUMO,<br>2.60% H-3 => LUMO,<br>2.08% H-8 => L+1
9	18163.606	550.551	3.000e-03	25.0% HOMO => L+1,<br>11.8% H-9 => LUMO,<br>7.16% H-4 => LUMO,<br>7.07% H-1 => L+1,<br>6.45% HOMO => L+2,<br>4.94% H-1 => L+3,<br>4.87% H-10 => LUMO,<br>3.55% H-6 => LUMO,<br>2.26% H-5 => LUMO
10	18289.428	546.764	1.430e-02	24.8% H-1 => L+1,<br>14.2% H-7 => LUMO,<br>13.0% HOMO => L+1,<br>6.72% H-3 => L+1,<br>5.16% H-2 => L+2,<br>3.56% H-2 => L+1,<br>3.36% H-3 => L+2,<br>2.87% H-2 => L+3,<br>2.70% H-3 => L+3
11	18466.064	541.534	5.100e-03	24.6% H-2 => L+1,<br>7.49% H-3 => L+1,<br>7.17% H-2 => L+3,<br>6.64% HOMO => L+2,<br>5.38% H-1 => L+1,<br>5.29% H-5 => LUMO,<br>4.98% HOMO => L+1,<br>4.29% H-6 => LUMO,<br>3.24% H-2 => L+2,<br>2.98% H-1 => L+3,<br>2.83% H-3 => LUMO,<br>2.47% H-4 => LUMO,<br>2.06% H-1 => L+2

Table S10, Continued. Calculated Electronic Transitions for $[Pd_6(S_2C_2Ph_2)_6]$.

Index	Energy (cm^{-1})	Wavelength	Oscillator Strength	Contributions
12	18674.961	535.476	6.000e-04	25.0% H-9 => LUMO,<br>17.1% H-11 => LUMO,<br>8.78% H-2 => L+2,<br>3.40% H-1 => L+3,<br>3.15% HOMO => L+1,<br>3.01% H-2 => L+3,<br>2.54% H-2 => L+1,<br>2.47% H-2 => L+4,<br>2.08% H-9 => L+2
13	18736.259	533.724	1.360e-02	25.6% H-9 => LUMO,<br>14.2% H-11 => LUMO,<br>6.08% H-1 => L+3,<br>5.42% HOMO => L+3,<br>5.23% H-1 => L+1,<br>4.01% H-6 => L+2,<br>2.66% H-8 => LUMO,<br>2.37% H-3 => L+1,<br>2.31% H-8 => L+3,<br>2.04% HOMO => L+1
14	18845.951	530.618	3.400e-03	12.2% HOMO => L+1,<br>8.14% H-3 => L+2,<br>7.40% H-3 => L+3,<br>7.27% H-3 => L+1,<br>4.90% H-11 => LUMO,<br>4.79% H-1 => L+1,<br>3.94% H-2 => L+1,<br>3.79% H-2 => L+2,<br>3.62% H-7 => LUMO,<br>3.33% HOMO => L+5,<br>3.32% H-3 => L+4,<br>3.21% H-5 => L+3,<br>2.98% H-1 => L+2
15	18887.892	529.440	3.000e-03	48.1% HOMO => L+5,<br>26.2% HOMO => L+4,<br>2.56% H-1 => L+5,<br>2.39% H-1 => L+4

Table S10, Continued. Calculated Electronic Transitions for $[Pd_6(S_2C_2Ph_2)_6]$.

Index	Energy (cm^{-1})	Wavelength	Oscillator Strength	Contributions
16	19102.435	523.493	1.790e-02	16.3% H-1 => L+2,<br>15.1% HOMO => L+3,<br>13.3% H-2 => L+3,<br>6.32% H-2 => L+2,<br>5.04% H-10 => LUMO,<br>5.03% H-9 => LUMO,<br>4.65% H-3 => L+3,<br>3.46% H-11 => LUMO,<br>3.04% H-2 => L+4,<br>2.94% H-5 => L+1
17	19287.136	518.480	1.400e-03	46.1% HOMO => L+2,<br>9.98% H-11 => LUMO,<br>9.15% HOMO => L+3,<br>7.80% H-8 => LUMO,<br>4.10% H-3 => L+1,<br>3.76% H-2 => L+2,<br>2.59% H-2 => L+1,<br>2.28% H-3 => L+3
18	19452.480	514.073	1.400e-03	38.7% H-10 => LUMO,<br>13.6% H-12 => LUMO,<br>6.88% HOMO => L+1,<br>6.45% H-7 => LUMO,<br>4.48% H-9 => LUMO,<br>3.20% H-3 => L+1,<br>2.72% H-2 => L+1
19	19665.410	508.507	4.000e-03	24.1% HOMO => L+3,<br>9.66% H-2 => L+3,<br>9.04% HOMO => L+2,<br>6.10% H-1 => L+2,<br>5.15% H-1 => L+1,<br>4.83% H-6 => L+3,<br>4.34% H-8 => LUMO,<br>3.69% H-11 => LUMO,<br>3.41% H-3 => L+3,<br>2.24% H-4 => L+1

Table S10, Continued. Calculated Electronic Transitions for $[Pd_6(S_2C_2Ph_2)_6]$.

Index	Energy (cm^{-1})	Wavelength	Oscillator Strength	Contributions
20	19983.193	500.421	5.200e-03	24.2% H-2 => L+1,<br>15.3% H-3 => L+1,<br>7.72% H-2 => L+2,<br>6.05% H-1 => L+3,<br>5.56% HOMO => L+1,<br>4.45% H-10 => LUMO,<br>2.52% H-3 => L+3,<br>2.51% H-3 => L+2,<br>2.15% H-8 => L+1,<br>2.08% H-9 => LUMO
21	20050.943	498.730	6.200e-03	13.6% H-1 => L+3,<br>11.1% HOMO => L+3,<br>9.81% H-1 => L+1,<br>6.68% HOMO => L+2,<br>6.62% H-2 => L+3,<br>6.55% H-12 => LUMO,<br>5.99% H-3 => L+3,<br>3.40% H-9 => LUMO,<br>3.35% H-10 => LUMO,<br>2.56% H-2 => L+2,<br>2.13% H-2 => L+1,<br>2.06% H-3 => L+1
22	20180.798	495.521	5.210e-02	15.1% H-1 => L+2,<br>8.98% H-3 => L+3,<br>6.77% H-12 => LUMO,<br>6.68% H-8 => L+2,<br>5.95% H-6 => L+3,<br>5.30% H-1 => L+1,<br>4.34% H-7 => L+1,<br>4.18% H-10 => LUMO,<br>2.70% H-2 => L+2,<br>2.38% H-13 => LUMO,<br>2.13% HOMO => L+3
23	20406.634	490.037	1.060e-02	30.7% H-12 => LUMO,<br>11.4% HOMO => L+3,<br>6.96% H-10 => LUMO,<br>6.73% H-1 => L+1,<br>6.34% H-3 => L+3,<br>5.78% H-1 => L+3,<br>5.15% H-2 => L+2,<br>4.60% H-1 => L+2,<br>3.23% H-2 => L+3,<br>2.13% H-2 => L+1

Table S10, Continued. Calculated Electronic Transitions for $[Pd_6(S_2C_2Ph_2)_6]$.

Index	Energy (cm^{-1})	Wavelength	Oscillator Strength	Contributions
24	20524.391	487.225	1.170e-02	23.1% H-1 => L+3,<br>18.9% H-3 => L+1,<br>8.96% H-2 => L+2,<br>8.43% H-2 => L+3,<br>8.25% H-11 => LUMO,<br>2.16% H-6 => LUMO,<br>2.10% H-2 => L+4
25	20609.079	485.223	1.230e-02	13.1% H-6 => L+1,<br>7.49% H-8 => L+1,<br>5.97% H-2 => L+1,<br>5.77% H-9 => L+3,<br>5.68% H-2 => L+3,<br>5.51% H-1 => L+2,<br>4.83% H-14 => LUMO,<br>3.50% H-7 => L+2,<br>3.46% H-15 => LUMO,<br>3.39% H-5 => L+1,<br>3.13% H-2 => L+2,<br>2.46% H-6 => L+3,<br>2.31% H-16 => LUMO
26	20805.071	480.652	1.210e-02	23.4% H-3 => L+2,<br>4.10% H-11 => LUMO,<br>4.09% H-5 => L+1,<br>3.52% H-7 => L+4,<br>3.43% H-9 => L+1,<br>3.28% H-12 => LUMO,<br>2.91% H-8 => L+2,<br>2.90% H-1 => L+5,<br>2.78% H-4 => L+5,<br>2.65% H-6 => L+2,<br>2.37% HOMO => L+3,<br>2.36% H-3 => L+1,<br>2.07% H-7 => L+3,<br>2.05% H-15 => LUMO

Table S10, Continued. Calculated Electronic Transitions for $[Pd_6(S_2C_2Ph_2)_6]$.

Index	Energy (cm^{-1})	Wavelength	Oscillator Strength	Contributions
27	20813.137	480.466	9.200e-03	10.6% H-4 => L+5,<br>7.30% H-7 => L+5,<br>6.19% H-1 => L+5,<br>5.99% H-15 => LUMO,<br>5.85% H-4 => L+1,<br>4.87% H-4 => L+4,<br>4.03% H-7 => L+4,<br>3.99% HOMO => L+5,<br>3.88% HOMO => L+4,<br>3.00% HOMO => L+1,<br>2.36% H-6 => L+1,<br>2.28% H-10 => L+5,<br>2.10% H-9 => L+1
28	21134.952	473.150	1.500e-03	9.72% H-16 => LUMO,<br>9.38% H-1 => L+4,<br>8.40% H-8 => L+2,<br>7.88% H-7 => L+1,<br>5.29% H-2 => L+2,<br>4.91% H-1 => L+5,<br>4.17% H-8 => L+3,<br>3.03% H-8 => L+1,<br>2.97% H-9 => L+1,<br>2.68% HOMO => L+3,<br>2.68% H-5 => L+3,<br>2.20% H-11 => L+1
29	21295.457	469.584	4.600e-02	22.0% H-1 => L+2,<br>14.6% H-13 => LUMO,<br>5.90% H-8 => L+2,<br>5.27% H-16 => LUMO,<br>3.97% HOMO => L+3,<br>3.59% H-2 => L+4,<br>2.17% H-1 => L+3,<br>2.06% H-5 => L+1,<br>2.05% H-7 => L+1

Table S11. Calculated Electronic Transitions for $[Pt_6(S_2C_2Ph_2)_6]$.

Index	Energy (cm^{-1})	Wavelength	Oscillator Strength	Contributions
0	17379.635	575.386	2.000e-04	38.8% H-1 => LUMO,<br>28.1% H-3 => LUMO,<br>8.58% H-2 => LUMO,<br>4.28% H-7 => LUMO,<br>3.69% H-6 => LUMO,<br>3.09% H-10 => LUMO,<br>2.82% H-5 => LUMO
1	17893.410	558.865	1.320e-02	52.4% HOMO => LUMO,<br>15.9% H-2 => LUMO,<br>14.6% H-3 => LUMO,<br>4.01% H-5 => LUMO
2	18024.072	554.814	4.900e-03	37.0% H-2 => LUMO,<br>32.7% HOMO => LUMO,<br>14.6% H-3 => LUMO,<br>3.27% H-8 => LUMO
3	18765.295	532.899	6.700e-03	39.2% H-1 => LUMO,<br>24.9% H-3 => LUMO,<br>20.6% H-2 => LUMO,<br>3.23% HOMO => LUMO,<br>2.49% H-5 => LUMO
4	19268.585	518.979	1.270e-02	37.4% H-4 => LUMO,<br>26.8% H-6 => LUMO,<br>7.47% H-7 => LUMO,<br>3.82% H-2 => L+1,<br>2.42% H-1 => LUMO,<br>2.10% 5=> LUMO
5	19579.109	510.748	9.500e-03	38.3% H-8 => LUMO,<br>28.0% H-5 => LUMO,<br>7.25% H-1 => LUMO,<br>3.55% HOMO => LUMO,<br>3.54% H-4 => LUMO,<br>3.08% H-7 => LUMO,<br>2.83% H-6 => LUMO
6	19825.108	504.411	1.900e-03	33.6% H-4 => LUMO,<br>28.4% H-7 => LUMO,<br>14.9% H-6 => LUMO,<br>4.49% H-3 => LUMO

Table S11, Continued. Calculated Electronic Transitions for $[Pt_6(S_2C_2Ph_2)_6]$.

Index	Energy (cm^{-1})	Wavelength	Oscillator Strength	Contributions
7	19962.222	500.946	5.300e-03	29.0% H-8 => LUMO,<br>19.8% H-5 => LUMO,<br>15.0% H-6 => LUMO,<br>11.2% H-7 => LUMO,<br>6.24% H-2 => LUMO
8	20497.774	487.858	1.200e-03	24.3% H-9 => LUMO,<br>14.1% H-5 => LUMO,<br>13.3% H-6 => LUMO,<br>11.4% H-10 => LUMO,<br>6.84% H-11 => LUMO,<br>5.95% H-7 => LUMO,<br>5.10% H-4 => LUMO,<br>3.55% H-8 => LUMO,<br>2.10% HOMO => L+1
9	20745.386	482.035	3.400e-03	28.6% H-11 => LUMO,<br>25.6% H-9 => LUMO,<br>11.5% H-10 => LUMO,<br>7.01% H-5 => LUMO,<br>4.07% H-7 => LUMO,<br>2.66% H-8 => LUMO,<br>2.19% H-6 => LUMO
10	20937.346	477.615	4.800e-03	32.4% H-9 => LUMO,<br>29.1% H-10 => LUMO,<br>9.48% H-11 => LUMO,<br>4.01% H-6 => LUMO,<br>2.20% H-8 => LUMO
11	22056.844	453.374	8.100e-03	16.1% H-7 => LUMO,<br>11.4% H-11 => LUMO,<br>10.4% H-1 => L+2,<br>9.53% H-10 => LUMO,<br>5.27% H-3 => L+2,<br>3.34% H-5 => LUMO,<br>3.31% H-7 => L+2,<br>3.18% H-8 => L+1,<br>2.88% H-3 => L+1,<br>2.65% H-2 => L+2,<br>2.14% H-9 => L+1

Table S11, Continued. Calculated Electronic Transitions for $[Pt_6(S_2C_2Ph_2)_6]$.

Index	Energy (cm^{-1})	Wavelength	Oscillator Strength	Contributions
12	22412.534	446.179	2.700e-03	10.9% H-10 => LUMO,<br>10.6% H-11 => LUMO,<br>8.15% H-8 => L+1,<br>6.10% H-3 => L+2,<br>4.92% H-5 => LUMO,<br>4.51% H-12 => LUMO,<br>4.22% H-6 => L+3,<br>3.63% H-1 => L+2,<br>3.44% H-1 => L+1,<br>2.66% H-4 => L+3,<br>2.51% H-8 => L+3,<br>2.36% H-6 => LUMO,<br>2.24% H-9 => L+3,<br>2.22% H-4 => LUMO,<br>2.08% H-3 => L+1
13	22697.248	440.582	7.500e-03	24.8% HOMO => L+1,<br>11.5% H-2 => L+1,<br>9.43% H-1 => L+1,<br>6.88% H-2 => L+3,<br>3.97% H-3 => L+3,<br>3.85% HOMO => L+2,<br>2.70% H-1 => L+3
14	22712.573	440.285	1.700e-03	34.3% HOMO => L+1,<br>13.5% H-3 => L+1,<br>9.16% HOMO => L+2,<br>6.18% H-2 => L+3,<br>5.63% H-10 => LUMO,<br>3.80% H-1 => L+1,<br>2.71% H-8 => L+2
15	22816.618	438.277	5.300e-03	33.5% H-2 => L+1,<br>10.6% H-1 => L+1,<br>8.82% H-3 => L+3,<br>7.68% H-1 => L+3,<br>3.77% H-12 => LUMO,<br>3.58% H-4 => LUMO,<br>2.36% H-1 => L+2,<br>2.17% H-2 => L+4,<br>2.04% H-4 => L+2

Table S11, Continued. Calculated Electronic Transitions for [$Pt_6(S_2C_2Ph_2)_6$].

Index	Energy (cm^{-1})	Wavelength	Oscillator Strength	Contributions
16	23007.772	434.636	2.400e-03	13.7% H-3 => L+3,<br>6.80% H-2 => L+3,<br>6.05% H-1 => L+3,<br>5.83% H-3 => L+1,<br>4.59% H-5 => L+3,<br>4.50% H-6 => L+1,<br>3.99% H-4 => L+1,<br>3.56% H-8 => L+3,<br>3.27% H-1 => L+1,<br>2.49% H-7 => L+1,<br>2.44% H-1 => L+4,<br>2.22% H-11 => LUMO
17	23132.788	432.287	1.780e-02	10.7% H-12 => LUMO,<br>9.78% H-9 => L+1,<br>8.43% H-2 => L+2,<br>7.13% H-4 => L+1,<br>6.98% H-11 => LUMO,<br>5.40% H-1 => L+1,<br>4.76% H-8 => L+3,<br>4.15% HOMO => L+2,<br>3.69% HOMO => L+3,<br>3.31% H-3 => L+2,<br>2.56% H-7 => LUMO
18	23246.512	430.172	2.300e-03	12.6% H-3 => L+1,<br>9.75% H-1 => L+1,<br>8.53% HOMO => L+1,<br>7.26% H-5 => L+1,<br>6.98% HOMO => L+2,<br>6.65% H-6 => L+2,<br>6.18% H-2 => L+1,<br>5.41% H-12 => LUMO,<br>3.07% H-5 => L+3,<br>2.42% H-6 => L+1,<br>2.02% H-6 => LUMO
19	23302.971	429.130	1.520e-02	21.3% HOMO => L+2,<br>19.9% H-3 => L+1,<br>6.43% H-2 => L+2,<br>3.59% H-7 => L+2,<br>2.58% H-11 => LUMO,<br>2.43% H-12 => LUMO,<br>2.41% H-2 => L+5,<br>2.05% H-4 => L+1

Table S11, Continued. Calculated Electronic Transitions for $[Pt_6(S_2C_2Ph_2)_6]$.

Index	Energy (cm^{-1})	Wavelength	Oscillator Strength	Contributions
20	23410.242	427.163	2.000e-02	27.3% H-12 => LUMO,<br>17.9% HOMO => L+2,<br>7.96% H-2 => L+2,<br>6.99% H-1 => L+1,<br>3.17% H-8 => LUMO,<br>3.10% H-6 => L+3
21	23715.926	421.658	3.900e-03	18.0% H-6 => L+1,<br>12.9% H-7 => L+1,<br>5.52% H-2 => L+3,<br>5.14% H-3 => L+2,<br>5.01% H-1 => L+3,<br>4.97% H-16 => LUMO,<br>4.52% HOMO => L+5,<br>4.37% H-5 => L+2,<br>3.30% H-13 => LUMO,<br>2.85% H-3 => L+5,<br>2.23% H-4 => L+1,<br>2.17% H-3 => L+1
22	23990.961	416.824	6.800e-03	15.0% H-1 => L+2,<br>12.9% HOMO => L+5,<br>6.29% HOMO => L+4,<br>5.48% H-3 => L+3,<br>4.07% HOMO => L+3,<br>4.00% HOMO => L+2,<br>3.17% H-2 => L+3,<br>2.50% H-2 => L+2,<br>2.34% HOMO => L+1,<br>2.32% H-8 => L+2,<br>2.30% H-2 => L+4,<br>2.15% H-7 => L+3
23	24084.522	415.204	3.300e-03	33.1% HOMO => L+5,<br>7.06% HOMO => L+4,<br>4.23% H-1 => L+5,<br>4.04% H-13 => LUMO,<br>2.84% H-1 => L+4,<br>2.81% HOMO => L+3,<br>2.34% H-3 => L+3,<br>2.29% H-7 => L+1,<br>2.11% H-6 => L+3

Table S11, Continued. Calculated Electronic Transitions for $[Pt_6(S_2C_2Ph_2)_6]$.

Index	Energy (cm^{-1})	Wavelength	Oscillator Strength	Contributions
24	24260.351	412.195	9.500e-03	15.0% H-12 => LUMO,<br>9.63% HOMO => L+3,<br>6.64% H-4 => L+1,<br>5.65% H-1 => L+3,<br>5.48% H-9 => L+1,<br>5.35% H-7 => L+1,<br>5.28% HOMO => L+2,<br>4.44% H-8 => L+2,<br>3.19% H-3 => L+3,<br>2.69% H-6 => L+3,<br>2.65% H-3 => L+2,<br>2.23% H-7 => L+3
25	24389.399	410.014	3.400e-03	16.5% H-8 => L+1,<br>8.68% H-1 => L+2,<br>7.18% H-1 => L+3,<br>6.61% H-2 => L+1,<br>5.09% H-9 => L+3,<br>4.31% H-6 => L+5,<br>4.17% H-7 => L+3,<br>3.81% HOMO => L+1,<br>3.35% H-14 => LUMO,<br>2.83% H-9 => L+2,<br>2.50% H-1 => L+1,<br>2.34% H-3 => L+2,<br>2.24% H-9 => LUMO
26	24424.888	409.418	2.400e-03	15.0% HOMO => L+3,<br>5.87% H-5 => L+1,<br>5.52% H-2 => L+1,<br>5.40% H-14 => LUMO,<br>5.06% H-1 => L+1,<br>4.48% H-8 => L+2,<br>4.19% H-1 => L+2,<br>3.80% H-2 => L+2,<br>3.61% H-15 => LUMO,<br>3.37% H-12 => LUMO,<br>3.15% H-4 => L+1,<br>2.41% H-7 => L+2,<br>2.11% H-5 => L+2,<br>2.08% H-3 => L+2

Table S11, Continued. Calculated Electronic Transitions for $[Pt_6(S_2C_2Ph_2)_6]$.

Index	Energy (cm^{-1})	Wavelength	Oscillator Strength	Contributions
27	24523.287	407.776	8.600e-03	7.84% H-12 => LUMO,<br>7.62% H-4 => L+1,<br>7.61% HOMO => L+3,<br>7.56% H-14 => LUMO,<br>5.56% HOMO => L+1,<br>4.85% H-8 => L+2,<br>4.71% H-9 => L+1,<br>4.55% H-15 => LUMO,<br>4.32% H-1 => L+3,<br>2.61% H-13 => LUMO,<br>2.15% H-4 => L+3,<br>2.14% H-7 => L+2
28	24631.366	405.986	1.400e-03	9.62% H-6 => L+5,<br>8.25% HOMO => L+3,<br>5.39% H-2 => L+2,<br>5.00% H-1 => L+2,<br>4.69% H-8 => L+1,<br>3.79% H-5 => L+5,<br>3.71% H-1 => L+1,<br>3.48% H-4 => L+5,<br>3.38% H-5 => L+1,<br>3.31% H-4 => L+2,<br>3.13% H-10 => L+5,<br>3.09% H-4 => L+3,<br>2.63% H-1 => L+3,<br>2.15% H-6 => L+2
29	24770.093	403.713	4.600e-03	12.4% H-3 => L+2,<br>9.02% H-1 => L+1,<br>7.20% HOMO => L+3,<br>5.83% H-2 => L+1,<br>4.23% H-4 => L+1,<br>4.22% H-2 => L+3,<br>3.41% H-6 => L+2,<br>3.26% H-9 => L+1,<br>3.06% H-6 => L+5,<br>3.04% H-8 => L+3,<br>2.84% H-3 => L+1,<br>2.73% H-2 => L+2,<br>2.52% H-13 => LUMO,<br>2.52% H-8 => L+1,<br>2.46% H-4 => L+5,<br>2.05% H-10 => LUMO,<br>2.00% H-5 => L+1
